# Artificial Intelligence and Large Language Models: A Case-Based, Peer-Teaching Workshop for Preclinical Medical Students

**DOI:** 10.15766/mep_2374-8265.11621

**Published:** 2026-07-21

**Authors:** Brendon C. Choy, Hariharan Shanmugam, Hyae Won Redden, Edgardo Duran, Jeffrey Prince, Richard S. Lee, Jacqueline Chan, Daniela Limbania, Jessica Berwick, Adam Rodman

**Affiliations:** 1 Fourth-Year Medical Student, Harvard Medical School; 2 Second-Year Medical Student, Harvard Medical School; 3 Assistant Professor of Medicine, Department of Medicine, Massachusetts General Hospital; Co-Director of PDW2, Harvard Medical School; 4 Assistant Professor of Medicine, Department of Medicine, Beth Israel Deaconess Medical Center; HMS Director of AI Programs, Harvard Medical School

**Keywords:** Artificial Intelligence, Large Language Models, Case-Based Learning

## Abstract

**Introduction:**

Artificial intelligence tools have rapidly become integrated in health care settings and are quickly affecting medical student education. However, there remains limited formal teaching on such tools in student curricula. This project sought to introduce preclerkship medical students to the basics of large language models and allow them to practice ways to best use these tools to supplement their learning.

**Methods:**

The authors designed, implemented, and evaluated a 60-minute lecture and 100-minute workshop for second-year medical students. The workshop included interactive cases covering various aspects of artificial intelligence use, and some groups were led entirely by student leaders, allowing for peer teaching.

**Results:**

One hundred sixty-eight students from Harvard Medical School and Harvard School of Dental Medicine were enrolled in this session. Anonymous pre- and postsession surveys (*N* = 124 and *N* = 62, respectively) were collected and compared via unpaired *t* test assuming unequal variance and showed statistically significant increase in the mean ratings of six 5-point Likert scale questions assessing artificial intelligence–related knowledge/self-efficacy (*P* < .01) and mixed changes to 5 questions relating to attitudes/behavioral intent.

**Discussion:**

Our artificial intelligence and large language model session provides a framework for teaching medical students, early in their educational journey, the basics of these tools. Our session provides interactive exercises to illustrate how best to leverage such tools while also discussing their potential risks. Such education will be important to incorporate into medical student curricula as artificial intelligence technologies grow increasingly common.

## Educational Objectives

By the end of this activity, learners will be able to:

1. Explain the potential risks of using artificial intelligence tools.
2. Summarize the differences in different types of large language models
3. Practice utilizing large language models to supplement studying, document summarization, evidence retrieval, and clinical decision-making.

## Introduction

Generative artificial intelligence (AI) technologies, such as large language models (LLMs), are among the fastest-adopted medical technologies in history.^1,2^ In a 2025 global survey of clinicians, nearly half have reported using AI for work purposes.^3^ Across health systems, different AI tools have been deployed in many aspects of patient care, from documentation assistance to clinical decision-making.^4,5^

LLMs also display remarkable abilities on cognitive tasks, passing USMLE exams and displaying immense performance in complex diagnostic reasoning tasks.^6,7^ Unsurprisingly, LLMs for decision support like OpenEvidence have become increasingly popular.^8^ They have also quickly been integrated into medical education.^9^ Given their ease of access, many students are using LLMs to supplement their studying.^9^ However, despite frequent student use of LLMs, there is worry about the potential risks such tools can have on the development of cognitive skills like critical thinking.^10,11^ Additionally, despite students’ general optimism about the incorporation of AI in health care, a majority have received little formal training on AI tools.^12,13^ As future leaders in health care, it is imperative that medical students receive education on these emerging tools, emphasizing both their efficient application and the careful consideration of associated risks.^14^

Growing consensus suggests that formal AI curricula are necessary in medical school.^14–16^ Medical educators and physicians are poised to play central roles in guiding appropriate AI use for their students.^16,17^ Currently, literature in this space has explored important competencies and frameworks for AI learning in a medical school curriculum or methods to embed AI into learning, but there is little published curricula aimed at teaching students effective methods of interacting with and using LLMs during their educational journeys.^15,18,19^

To address gaps in curricula and promote the development of student proficiency with AI tools amid their growing popularity, we engaged medical student educators and content experts to develop a didactic and workshop session for preclinical second-year medical students, soon to enter their clinical experiences at Harvard Medical School (HMS). The provided teaching material and interactive case-based, peer-led small group format are novel and build on existing primers, which provide only basic grounding on AI and its applications.^20^ Additionally, our materials are catered toward a medical school curriculum; however, the general structure of this session can be adapted with other domain-specific cases to teach health professions students of other fields similarly foundational skills.

## Methods

### Curriculum Development

In August 2025, all second-year medical students at HMS and dental students at the Harvard School of Dental Medicine (HSDM), who also complete the entire medical school preclerkship curriculum, participated in a 3-hour session dedicated to integrating AI curricula. Prior to this, we, a faculty member and 8 medical students, developed the curricula through multiple virtual meetings.^21^

We created 2 educational resources: first, a 60-minute lecture introducing AI tools, ethical considerations, and risks (Appendix A), and second, a 100-minute workshop (Appendix B). The aim of this workshop was to provide 5 case-based exercises, split across 3 parts, to explore important domains in which medical students could use AI tools. Case 1 covers using LLMs in studying, case 2 covers using LLMs to summarize a document, case 3 covers using LLMs to find evidence, and cases 4 and 5 cover using LLMs to aid in clinical reasoning. These cases also served as a starting point for discussions with peers and students about the strengths and limitations of LLM tools in later phases of training. AI, specifically GPT4.1, was used to create case 1 to demonstrate how AI can be used to create practice questions. We take responsibility for this AI-generated content.

Our educational session was informed by principles of adult learning theory and Kolb's experiential learning theory, using case-based learning and near-peer teaching.^22–24^ The workshop activities provided concrete experiences to apply LLMs in relevant studying and clinical scenarios. Additionally, learners engaged in guided reflection on their real-time outputs, discussed generalizable principles for safe and effective use, and actively experimented by refining their prompting strategies and tool selection throughout the session. Furthermore, we used near-peer teaching to facilitate appropriately contextualized discussions of LLM use for students.^25^ This design ultimately was intended to produce measurable changes in learners’ self-reported LLM knowledge and confidence in applying these AI tools to relevant domains.

### Facilitators

All 8 medical students and the faculty member who co-created this session also served as facilitators. All facilitators helped develop a teaching script to standardize every workshop session (Appendix C).

### Session Logistics and Implementation

All second-year medical and dental students at HMS/HSDM were expected to attend this session. The students did not have preparatory work but were told to bring their laptops. All students were provided with a supplemental case list (Appendix D) and an optional postsession editorial reading electronically.^26^

The session began with an in-person 60-minute lecture to all students. Learners were then randomly assigned to one of five 100-minute workshop sessions. Four of these workshop sessions had approximately 24 students and were facilitated by a paired fourth-year and second-year student. The fifth workshop group had approximately 72 students and was facilitated by a faculty member. The number of groups and students per group was set based on the number of teachers available and the room size capacity.

All workshop sessions were in different rooms but occurred concurrently. The slides provided in Appendix B were projected in each room. During the workshop, students were expected to use their laptops to complete exercises. They were free to use any LLM of their choosing, including web models or those provided in the Harvard University AI Sandbox, an institutional resource. At other institutions, any free or accessible LLM can be used.

As outlined previously, each case served to provide an interactive way for students to engage LLMs in some educational or clinically relevant domain. In case 1, students were provided with a question, similar to one they might encounter while studying. They then compared information collected between traditional resources (like UpToDate, Google) and LLMs. Students also modified their LLM prompts to provide more effective outputs, and takeaways on using LLMs to supplement studying were discussed.

The second part of the workshop involved reviewing document retrieval and use of AI tools to find evidence. In case 2, students uploaded a document and summarized it using an LLM. Students could use a review article electronically supplied by the instructors, and a cited cirrhosis review article was provided in the presenter guide and case list as an example.^27^ In case 3, students were first asked to collect evidence on a clinical question using traditional resources and then to use 2 different LLM models. They subsequently discussed the process of finding evidence using traditional methods vs LLMs and compared the evidence provided by the models.

The final part of the workshop covered the use of AI tools for clinical reasoning. Students were provided 2 clinical cases. In the first case (case 4), they had to think through the case without AI, then use AI to add to their initial thoughts. In the second case (case 5), students directly turned to AI, then added their own thoughts. Juxtaposing these 2 cases allowed for discussion and reflection on potential pitfalls of AI overreliance. This exercise also allowed for a transition into discussing risks of AI use and on the value of persistent knowledge and learning.

### Evaluation and Data Analysis

We accessed the session using pre- and postsession Qualtrics XM surveys to assess learner knowledge, attitudes, and experience (Appendix E). The pre- and postsession surveys were optional, anonymous, and provided via QR code at the start and end of the session. Surveys consisted of questions utilizing a 5-point Likert scale. Educational objective 1 relating to LLM risks was addressed in the session but not quantitatively assessed. To assess Kirkpatrick level 1 (perception of learning), both surveys contained identical questions assessing self-reported knowledge regarding differences among LLM model types and self-reported comfort and attitudes with respect to using LLMs for studying, document summarization, and evidence retrieval, as well as clinical decision support.^28^ We also included exploratory items on perceived impacts of LLM use on the development of clinical reasoning and education to examine whether attitudes toward LLM use shifted after the workshop's content on LLM capabilities and risks. The presession survey also collected baseline information on AI tool use, and the postsession survey assessed general reaction/satisfaction. For survey development, we performed member checking on 2 members of the facilitation team who had no part in developing the survey to ensure reliability. These members provided oral feedback, and the process was continued with iterative changes until we met saturation.

Because medical and dental students at our institution share the same curricula, students from both schools were grouped for analysis. For statistical analysis of these quantitative data, mean values to corresponding questions were compared using *t* tests assuming unequal variance in Microsoft Excel. To account for multiple comparisons across survey items, the Holm-Bonferroni correction was applied. Unadjusted *P* values were reported, and statistical significance after correction was assessed using a 2-sided α of 0.05.

Qualitatively, data was collected in a separate OASIS (Online Academic Student Information System) survey by course co-directors, which asked students to reflect on the didactic and workshop and respond to the 3 following prompts: (1) “One thing that I learned in this session that I will incorporate into my work …,” (2) “One thing I would like to see changed about this session for future iterations is …,” and (3) “Please share any additional thoughts about this session.” This information is routinely collected. Prompt 1 provides insight into elements of Kirkpatrick level 1 (reaction) outcomes by gathering statements of self-reported knowledge gained across the 3 educational objectives. In addition, we conducted sentiment analysis of prompt 2 with the application of inductive codes using a grounded constructivist approach. We developed themes from participants’ responses and grouped them into categories including those providing positive/neutral feedback, requesting additions to the session, or providing negative/constructive feedback.

This study was reviewed by the HMS Program in Medical Education Educational Scholarship Committee and deemed to be an Educational Quality Improvement project.

## Results

### Quantitative Results

One hundred sixty-eight second-year medical and dental students at HMS/HSDM participated in this session. The presession survey was completed by 73.8% of students (*N* = 124), and the postsession survey was completed by 36.9% of students (*N* = 62).

Based on data from the presession survey on average, students reported using AI tools for studying between “Sometimes” and “Often” and for clinical activities between “Rarely” and “Sometimes.” Three questions only asked on the postsurvey session show that on average, students reported that they “Agree” or “Strongly Agree” with feeling comfortable expressing their views in the workshop and feeling satisfied by the didactic and workshop portions (Table 1).

**Table 1.**
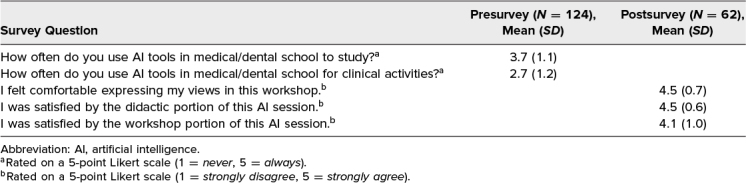
Presurvey or Postsurvey Only Questions

Eleven questions were asked in both the pre- and postsession survey, which can be categorized into 6 questions about knowledge/self-efficacy and 5 questions about attitude/behavioral intention. For all 6 knowledge/self-efficacy questions, there were statistically significant increases in mean Likert scale scores when comparing the 2 surveys after Holm-Bonferroni correction (Table 2). For attitude/behavioral intention questions, there was an increase in mean intent to use AI tools to help with creating a differential and in providing confidence to clinical management decisions on unadjusted analysis but no statistically significant difference in any item after correction (Table 3).

**Table 2.**
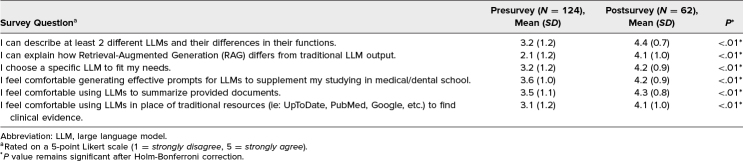
Knowledge/Self-Efficacy Quantitative Results

**Table 3.**
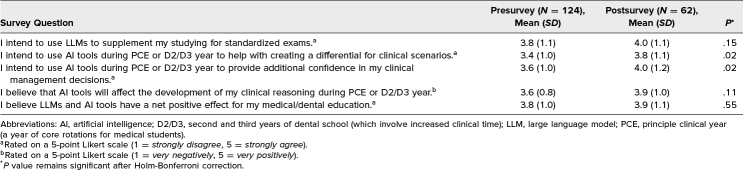
Attitude/Behavioral Intention Quantitative Results

### Qualitative Results

We collected 168 responses to our open-ended questions. When asked “One thing that I learned in this session that I will incorporate into my work…,” students reported that they would incorporate in their future work a variety of learning points including ethical considerations, risks, and use of AI tools in the domains taught. Using sentiment analysis of responses to the prompt “One thing I would like to see changed about this session for future iterations is…,” we categorized responses into 3 themes: (1) those reporting only positive feedback or no changes, (2) responses suggesting additions to the session, or (3) responses providing negative feedback or suggestions for changes. Nearly half (42.9%) of responses were exclusively positive or suggested no changes and included statements like “I thought this session was run great!” or “N/A.” Additionally, 27.4% suggested additional topics they felt warranted additional time like “a more comprehensive look at what health systems are currently using and trying” or “more small-group time to share thoughts and uses for AI.” Remaining responses suggested changes like making it “shorter” or “asynchronous,” or alluded to the workshop's limited utility due to prior exposure to AI tools or content.

## Discussion

We created a 2-part didactic and workshop session to teach and initiate discussions around AI for second-year preclinical medical and dental students. Our session provided practical knowledge about LLMs, skills-based exercises to demonstrate their use, and spaces for nuanced discussion surrounding both the benefits and risks of their implementation. Such knowledge can benefit students as they enter health care systems that will increasingly use AI tools.

Strengths of our session include the breadth of topics covered, including several aspects of AI tool use tailored for medical students (studying, document summarization, evidence retrieval, clinical decision-making), positive reception, and improvements to self-reported knowledge/self-efficacy. On average, students felt comfortable expressing themselves and satisfied with the session and had statistical improvements to all knowledge/self-efficacy–based questions, which indicated success in at least perceived learning.

Another strength is the incorporation of near-peer teaching. Student pairs led 4 of the 5 workshop groups. Student teachers allow for a more comfortable environment for other students to discuss use of AI tools, discuss views on them, and ask questions. Additionally, fourth-year students could directly provide their perspective from their experiences on the wards and provide guidance that may be more closely aligned with the current realities of clinical training. At the same time, second-year learners could engage in peer teaching and provide insight on the most relevant material for their own class. Such peer teaching is of recognized value in medical student development and may be especially helpful during periods of technological change and uncertainty.^29,30^

Notably, questions related to how student behaviors and attitudes were affected had mixed results. There was no significant change in students’ intent to use LLMs for studying. Many students already reported using AI tools for studying in the presurvey, so their study habits may already be relatively static at this stage. Additionally, in the unadjusted analysis, there was a trend toward increased mean intent to use AI tools for clinically relevant activities like creating a differential or providing confidence in clinical management decisions. This might reflect the students’ reduced reported presession usage of AI for clinical activities and the novel exposure provided by the workshop. Nonetheless, such differences were not statistically significant after correction. In addition, there was no significant change in attitudes on the effect of AI tools on the development of clinical reasoning or education. This workshop was intended to show both best practices using these tools and highlight the risks of using them. In alignment with these goals, review of arguments both in favor of and against the use of AI may have contributed to the limited overall change in intent to use AI tools clinically and attitudes with respect to their effect on educational development.

Regarding areas of improvement for future sessions, if more student teachers were available, we would have preferred smaller group sizes and more student-run workshops. Smaller group sizes can allow for more personalized discussions and interaction during workshop exercises. Additionally, students might benefit from having the studying part of the workshop earlier on in their medical education. For example, the workshop could be split into its 3 parts and delivered across multiple sessions over the course of a first-year longitudinal AI curriculum. In future iterations, additional topics, some suggested by our student feedback, could be added, like how health systems are using AI tools, or additional time could be given to comparing different model types and prompts.

There were several limitations to this study. First, only half of the respondents completed the postsurvey compared to the presurvey. More purposeful integration of the postsurvey at the end of the workshops might have increased yield. Second, there could have been differences among facilitators, particularly the workshop led by the faculty member compared to student leaders, leading to a more variable experience for individuals. Students may be less willing to share with a faculty member, and student leaders may tailor tips and discussion based on their current experiences with studying, student responsibilities, and entering clinical experiences. We would have liked to analyze how the faculty-led workshop session compared to student-run sessions or compare the sessions between students, but insufficient data were collected.

Third, we recognize potential variability in AI model access for educators and students. However, any accessible tool can be used with these cases, whether it be a free online model or institution-specific platform, and should not significantly affect facilitation or experience of the workshop. In fact, diversity in tools should be encouraged as students can compare and reflect on the different model outputs to specific prompts.

Finally, prior to this session, students have had varying exposure to these AI tools. Those with more experience may benefit less from this session compared to those with less experience, as reflected in some of the qualitative feedback. Thus, in the future, stratifying students by prior experience with AI tools might be useful in tailoring learning objectives, exercises, and discussions. As AI tools continue to develop, the wide fluctuation in baseline knowledge around this topic will likely present a challenge for educators seeking to provide appropriate guidance on AI.

In conclusion, we created a lecture and workshop aimed to incorporate education on AI tools into the medical school curriculum and used a case-based, peer-teaching model. We also allowed spaces for discussion regarding the nuanced use of these tools in clinical and educational contexts, and such discussions will likely continue to be important for students as they learn and grow in a world increasingly adopting AI tools. As these tools rapidly change and familiarity with them changes year-to-year, such teaching also will likely need to continually be updated. However, AI tools are likely to persist and institutional integration of this curricula would be valuable for any medical student in their initial phases of training. Additionally, these fundamental concepts and explorations of AI tools are important beyond medical students. These materials also provide a structured outline that can be adapted with content or cases from other fields to appropriately teach and engage students across many other health professions.

## Appendices


AI Didactic.pptxAI Workshop.pptxAI Workshop Presenter Guide.docxAI Workshop Case List.docxPre- and Postsurvey.docx
*All appendices are peer reviewed as integral parts of the Original Publication.*

## References

[1] Busch F, Hoffmann L, Rueger C, et al. Current applications and challenges in large language models for patient care: a systematic review. Commun Med (Lond). 2025;5(1):26. 10.1038/s43856-024-00717-2 39838160 PMC11751060

[2] Alowais SA, Alghamdi SS, Alsuhebany N, et al. Revolutionizing healthcare: the role of artificial intelligence in clinical practice. BMC Med Educ. 2023;23(1):689. 10.1186/s12909-023-04698-z 37740191 PMC10517477

[3] Goodchild L, Mulligan A, West C, Mansell N. Clinician of the Future 2025. Elsevier. 2025. Accessed September 1, 2025. https://assets.ctfassets.net/o78em1y1w4i4/T7F5sDDiUC8KJzLQXfJoy/004be7f43562d318115a294cf626be7f/ClinicianOfTheFuture_2025.pdf

[4] Tierney AA, Gayre G, Hoberman B, et al. Ambient artificial intelligence scribes: learnings after 1 year and over 2.5 million uses. NEJM Catal Innov Care Deliv. 2025;6:5. 10.1056/cat.25.0040

[5] Khosravi M, Zare Z, Mojtabaeian SM, Izadi R. Artificial intelligence and decision-making in healthcare: a thematic analysis of a systematic review of reviews. Health Serv Res Manag Epidemiol. 2024;11:23333928241234863. 10.1177/23333928241234863 38449840 PMC10916499

[6] Kung TH, Cheatham M, Medenilla A, et al. Performance of ChatGPT on USMLE: potential for AI-assisted medical education using large language models. PLOS Digit Health. 2023:2(2):e0000198. 10.1371/journal.pdig.0000198 36812645 PMC9931230

[7] Goh E, Gallo R, Hom J, et al. Large language model influence on diagnostic reasoning: a randomized clinical trial. JAMA Netw Open. 2024;7(10):e24440969. 10.1001/jamanetworkopen.2024.40969 PMC11519755 39466245

[8] OpenEvidence Creates the First AI in History to Score a Perfect 100% on the United States Medical Licensing Examination (USMLE). OpenEvidence. August 15, 2025. Accessed September 1, 2025. https://www.openevidence.com/announcements/openevidence-creates-the-first-ai-in-history-to-score-a-perfect-100percent-on-the-united-states-medical-licensing-examination-usmle

[9] Ganjavi C, Eppler M, O'Brien D, et al. ChatGPT and large language models (LLMs) awareness and use. A prospective cross-sectional survey of U.S. medical students. PLOS Digit Health. 2024;3(9):e0000596. 10.1371/journal.pdig.0000596 39236008 PMC11376538

[10] Abd-alrazaq A, AlSaad R, Alhuwail D, et al. Large language models in medical education: opportunities, challenges, and future directions. JMIR Med Educ. 2023;9:e48291. 10.2196/48291 37261894 PMC10273039

[11] Gerlich M. AI tools in society: impacts on cognitive offloading and the future of critical thinking. Societies. 2025;15(1):6. 10.3390/soc15010006

[12] Busch F, Hoffman L, Truhn D, et al. Global cross-sectional student survey on AI in medical, dental, and veterinary education and practice at 192 faculties. BMC Med Educ. 2024;24(1):1066. 10.1186/s12909-024-06035-4 39342231 PMC11439199

[13] Tan S, Xin X, Wu D. ChatGPT in medicine: prospects and challenges: a review article. Int J Surg. 2024;110(6):3701–3706. 10.1097/JS9.0000000000001312 38502861 PMC11175750

[14] Pupic N, Ghaffari-zadeh A, Hu R, et al. An evidence-based approach to artificial intelligence education for medical students: a systematic review. PLOS Digit Health. 2023;2(11):e0000255. 10.1371/journal.pdig.0000255 38011214 PMC10681314

[15] Tolentino R, Baradaran A, Gore G, Pluye P, Abbasgholizadeh-Rahimi S. Curriculum frameworks and educational programs in AI for medical students, residents, and practicing physicians: scoping review. JMIR Med Educ. 2024;10:e54793. 10.2196/54793 39023999 PMC11294785

[16] Abdulnour REE, Gin B, Boscardin CK. Educational strategies for clinical supervision of artificial intelligence use. N Engl J Med. 2025; 393(8):786–797. 10.1056/NEJMra2503232 40834302

[17] Li Z, Li F, Fu Q, et al. Large language models and medical education: a paradigm shift in educator roles. Smart Learn Environm. 2024;11:26. 10.1186/s40561-024-00313-w

[18] Singla R, Pupic R, Ghaffarizadeh S, et al. Developing a Canadian artificial intelligence medical curriculum using a Delphi study. NPJ Digit Med. 2024;7(1):323. 10.1038/s41746-024-01307-1 39557985 PMC11574260

[19] Brügge E, Ricchizzi S, Arenbeck M, et al. Large language models improve clinical decision making of medical students through patient simulation and structured feedback: a randomized controlled trial. BMC Med Educ. 2024;24(1):1391. 10.1186/s12909-024-06399-7 39609823 PMC11605890

[20] Agarwal G, Ramamoorthi L, Yuen T, et al. Exploring applications of artificial intelligence tools in clinical care and health professions education: an online module for students. MedEdPORTAL. 2025;21:11524. 10.15766/mep_2374-8265.11524 40313714 PMC12043951

[21] Schlegel EFM, Bird JB, Burns CM, et al. Curriculum design and scholarship for new educators: a professional development workshop for medical students. MedEdPORTAL. 2021;17:11130. 10.15766/mep_2374-8265.11130 33928186 PMC8071841

[22] Mukhalalati BA, Taylor A. Adult learning theories in context: a quick guide for healthcare professional educators. J Med Educ Curric Dev. 2019;6:2382120519840332. 10.1177/2382120519840332 31008257 PMC6458658

[23] Kolb D. Experiential Learning as the Science of Learning and Development. Prentice Hall; 1984.

[24] McLean SF. Case-based learning and its application in medical and health-care fields: a review of worldwide literature. J Med Educ Curric Dev. 2016;3:JMECD.S20377. 10.4137/JMECD.S20377 29349306 PMC5736264

[25] Kusnoor AV, Balchandani R, Pillow MT, Sherman S, Ismail N. Near-peers effectively teach clinical documentation skills to early medical students. BMC Med Educ. 2022;22(1):712. 10.1186/s12909-022-03790-0 36209076 PMC9548193

[26] Dhaliwal G. ‘This time is different’: physician knowledge in the age of artificial intelligence. BMJ Qual Saf. 2024;33(9):549–551. 10.1136/bmjqs-2024-017141 38702181

[27] Tapper EB, Parikh ND. Diagnosis and management of cirrhosis and its complications: a review. JAMA. 2023;329(18):1589–1602. 10.1001/jama.2023.5997 37159031 PMC10843851

[28] Rouse DN. Employing Kirkpatrick's evaluation framework to determine the effectiveness of health information management courses and programs. Perspect Health Inf Manag. 2011;8(spring):1c. PMC3070232 21464860

[29] Avonts M, Bombeke K, Michels NR, Vanderveken OM, De Winter BY. How can peer teaching influence the development of medical students? A descriptive, longitudinal interview study. BMC Med Educ. 2023;23(1):861. 10.1186/s12909-023-04801-4 37957668 PMC10644508

[30] Tanveer MA, Mildestvedt T, Skjærseth IG, et al. Peer teaching in undergraduate medical education: what are the learning outputs for the student-teachers? A systematic review. Adv Med Educ Pract. 2023;14:723–729. 10.2147/AMEP.S401766 37455859 PMC10349571

