# Supplementary material for: Artificial Intelligence and Large Language Models: A Case-Based, Peer-Teaching Workshop for Preclinical Medical Students
Source: MedEdPORTAL. 2026 Jul 21;22:11621. doi: 10.15766/mep_2374-8265.11621 (PMC13385069; doi:10.15766/mep_2374-8265.11621)
Supplement: Supplementary file 1 — AI Didactic.pptxAI Workshop.pptxAI Workshop Presenter Guide.docxAI Workshop Case List.docxPre- and Postsurvey.docx [file mep_2374-8265.11621-s001.zip › B. AI Workshop.pptx]

## Slide 1
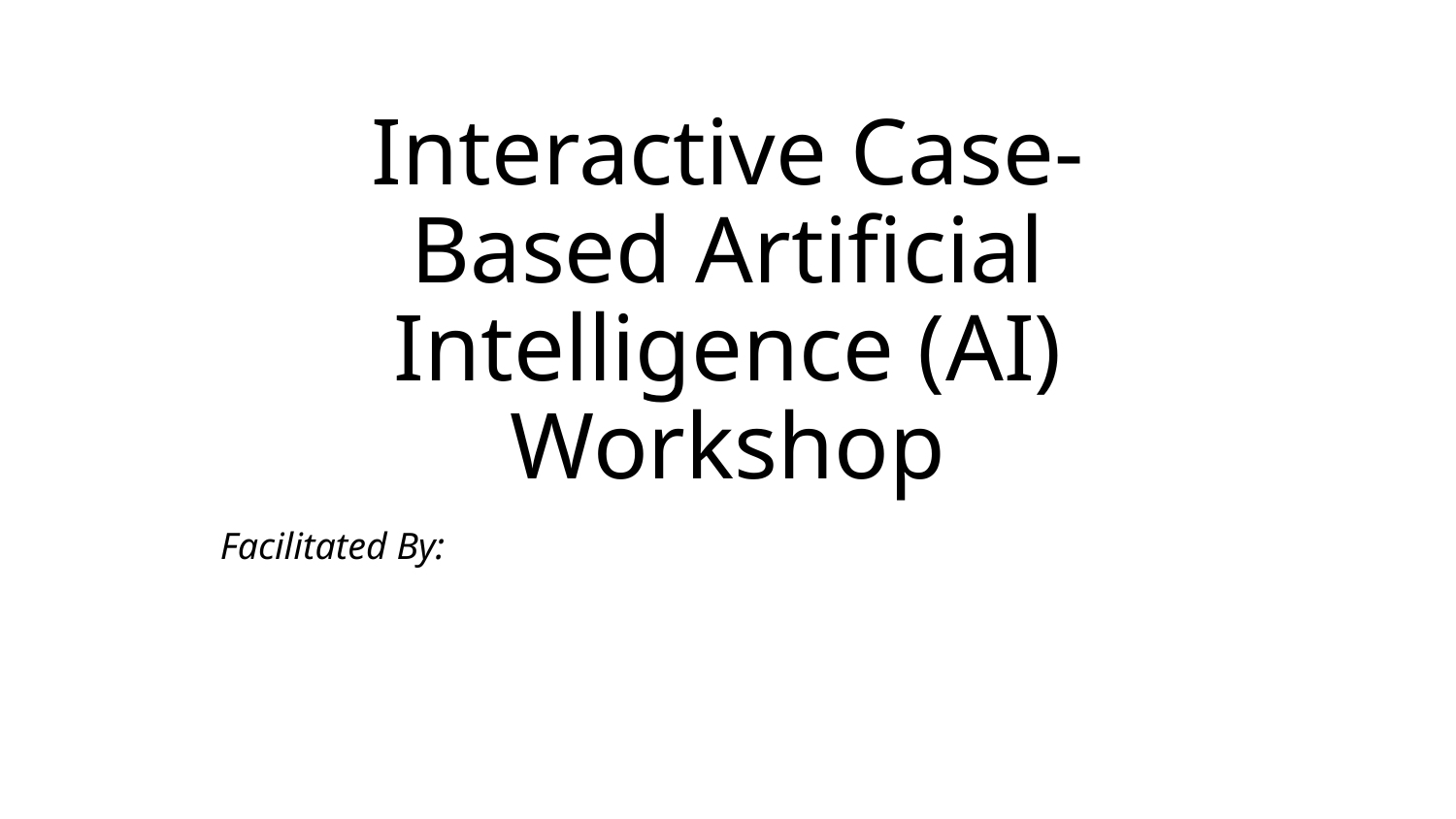

# Interactive Case-Based Artificial Intelligence (AI) Workshop
Facilitated By:

## Slide 2
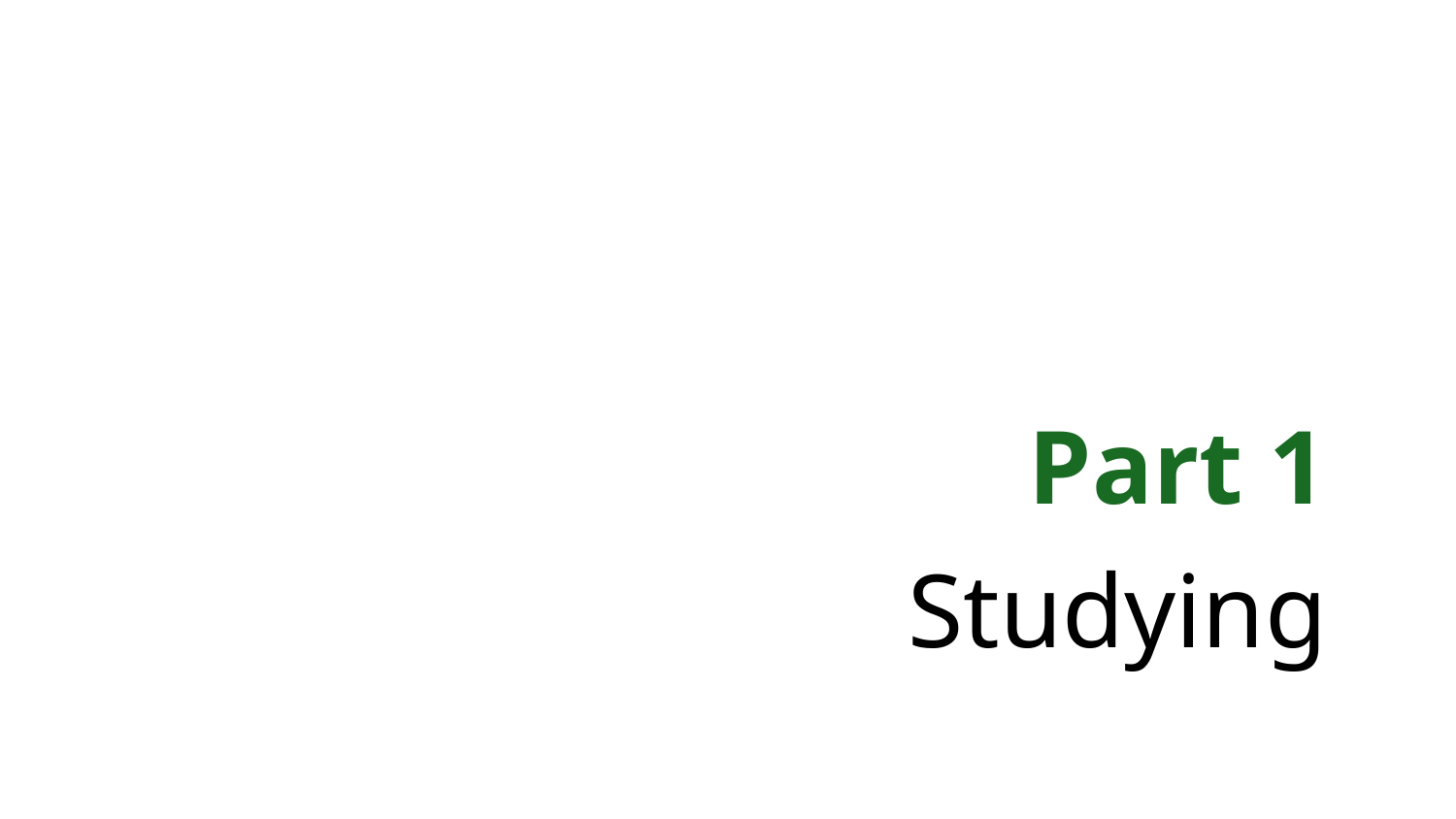

Part 1
# Studying

## Slide 3
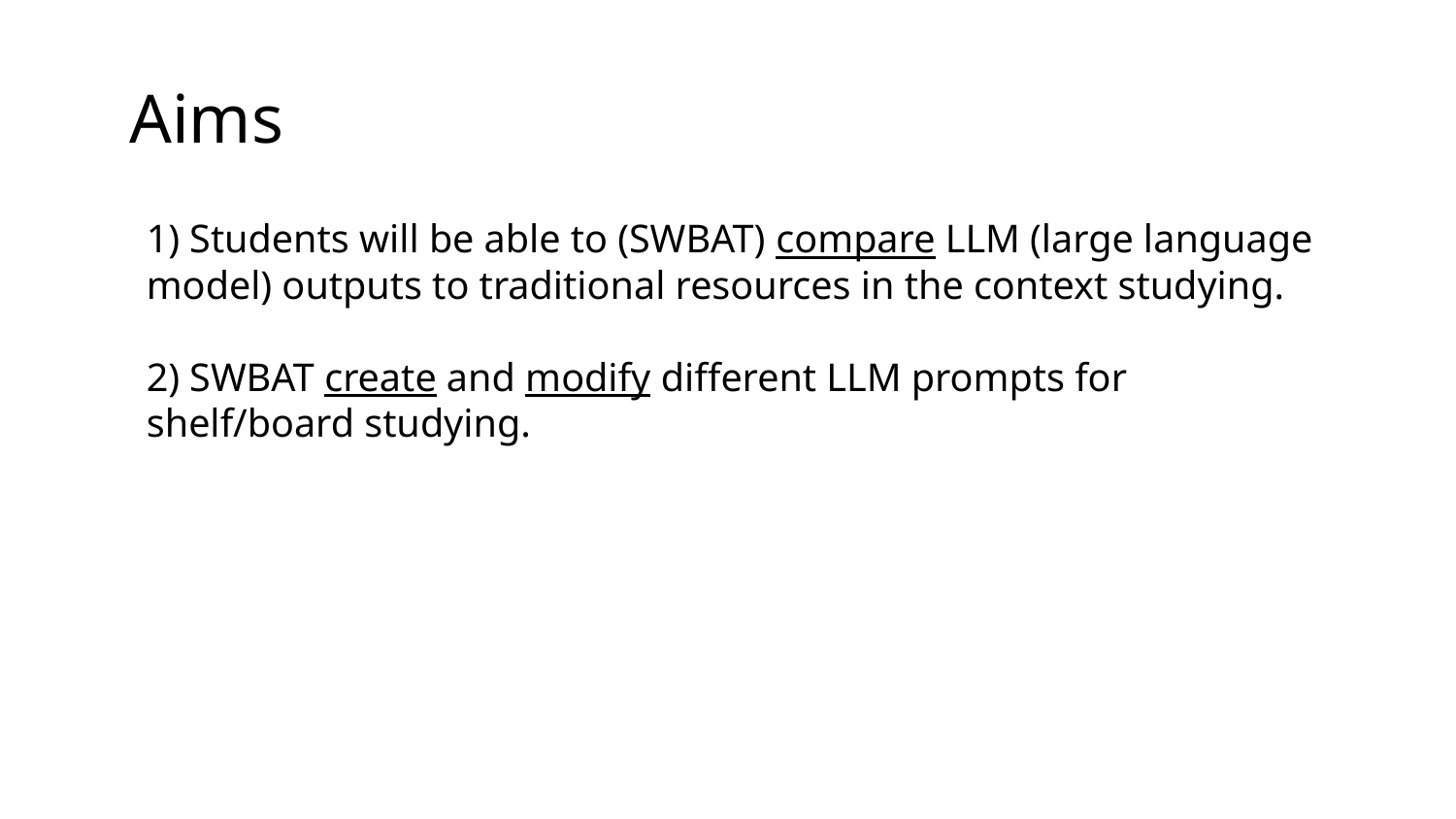

# Aims
1) Students will be able to (SWBAT) compare LLM (large language model) outputs to traditional resources in the context studying.
2) SWBAT create and modify different LLM prompts for shelf/board studying.

## Slide 4
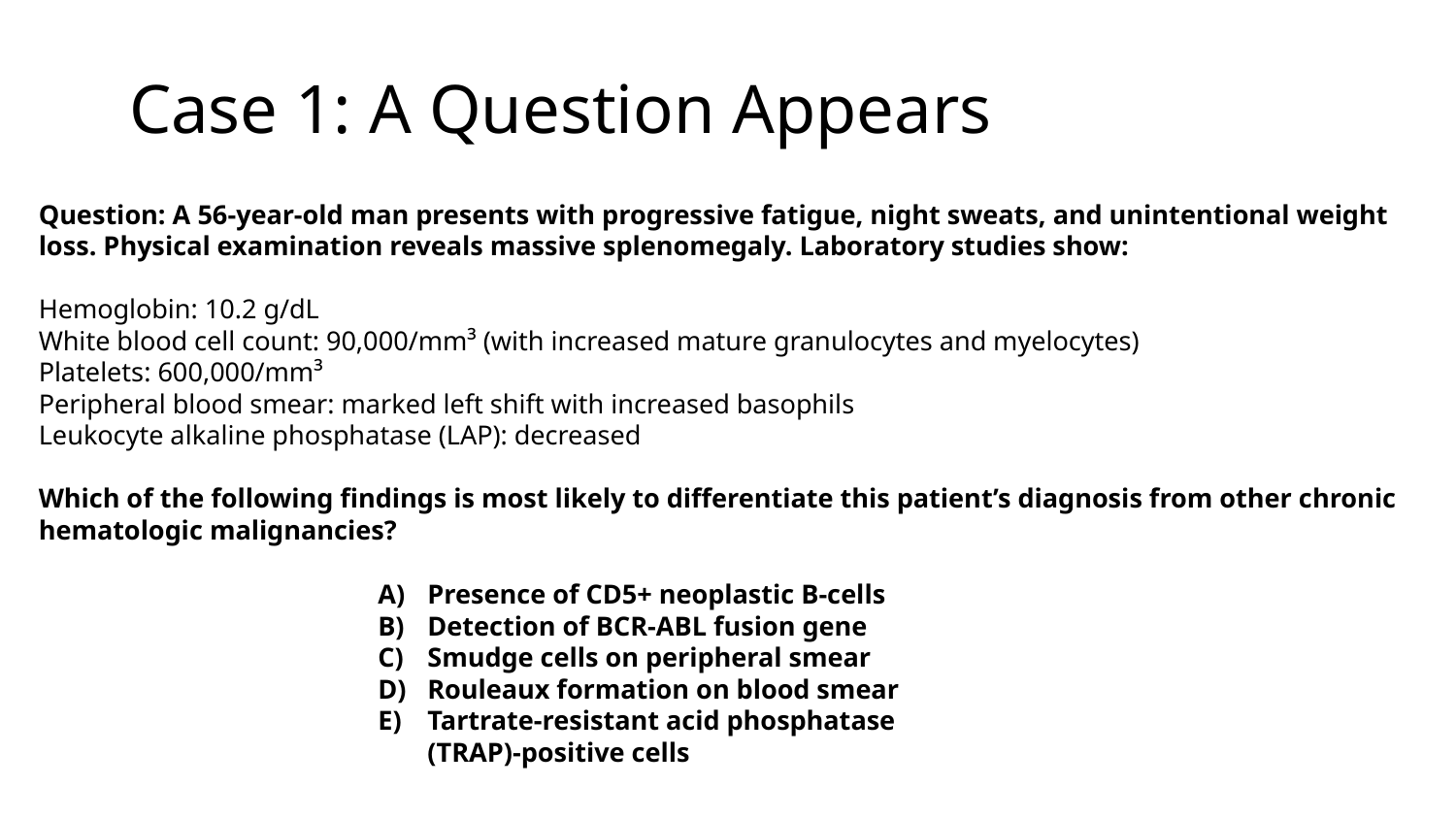

# Case 1: A Question Appears
Question: A 56-year-old man presents with progressive fatigue, night sweats, and unintentional weight loss. Physical examination reveals massive splenomegaly. Laboratory studies show:
Hemoglobin: 10.2 g/dL
White blood cell count: 90,000/mm³ (with increased mature granulocytes and myelocytes)
Platelets: 600,000/mm³
Peripheral blood smear: marked left shift with increased basophils
Leukocyte alkaline phosphatase (LAP): decreased
Which of the following findings is most likely to differentiate this patient’s diagnosis from other chronic hematologic malignancies?
Presence of CD5+ neoplastic B-cells
Detection of BCR-ABL fusion gene
Smudge cells on peripheral smear
Rouleaux formation on blood smear
Tartrate-resistant acid phosphatase (TRAP)-positive cells

## Slide 5
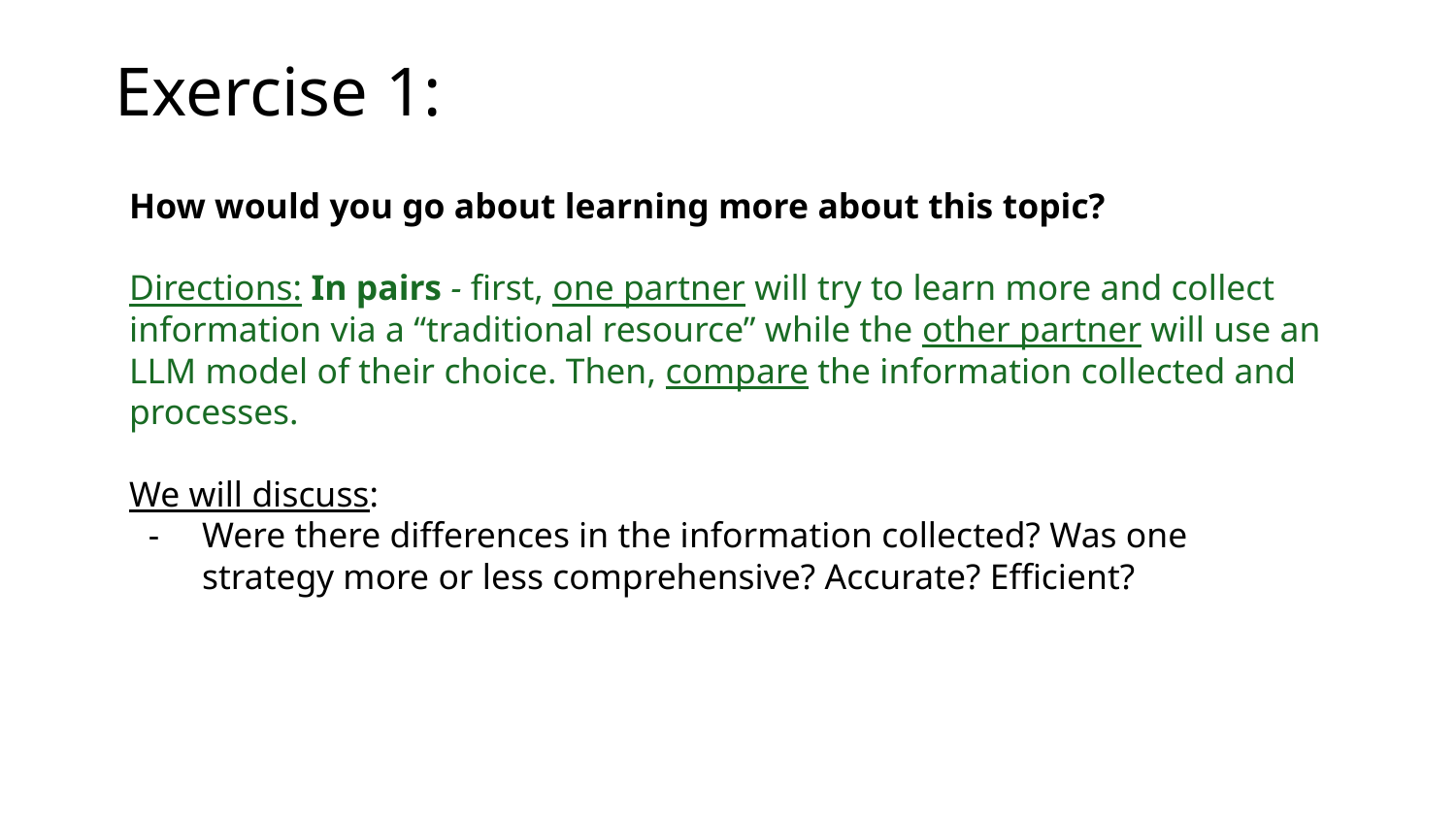

# Exercise 1:
How would you go about learning more about this topic?
Directions: In pairs - first, one partner will try to learn more and collect information via a “traditional resource” while the other partner will use an LLM model of their choice. Then, compare the information collected and processes.
We will discuss:
Were there differences in the information collected? Was one strategy more or less comprehensive? Accurate? Efficient?

## Slide 6
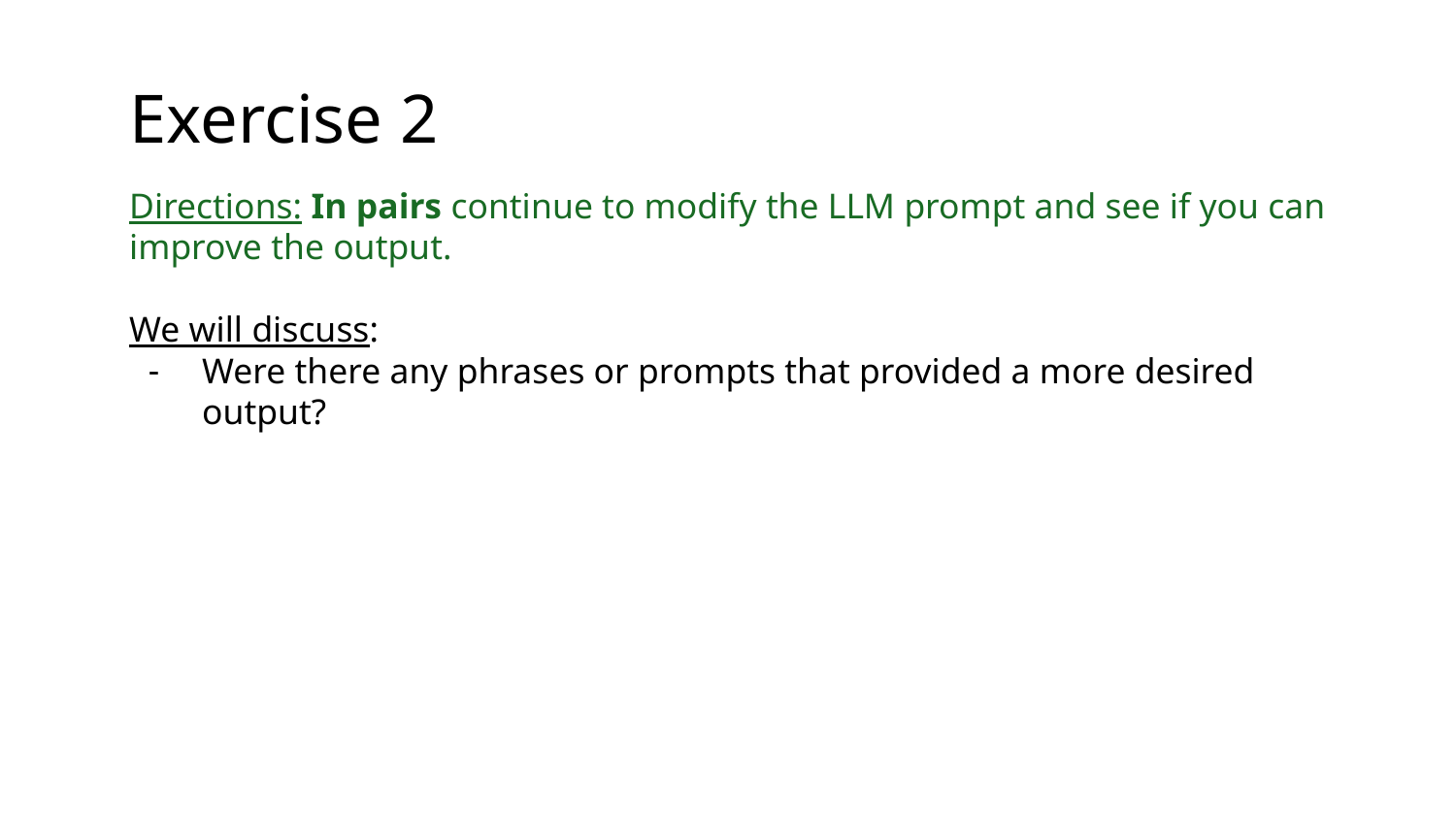

# Exercise 2
Directions: In pairs continue to modify the LLM prompt and see if you can improve the output.
We will discuss:
Were there any phrases or prompts that provided a more desired output?

## Slide 7
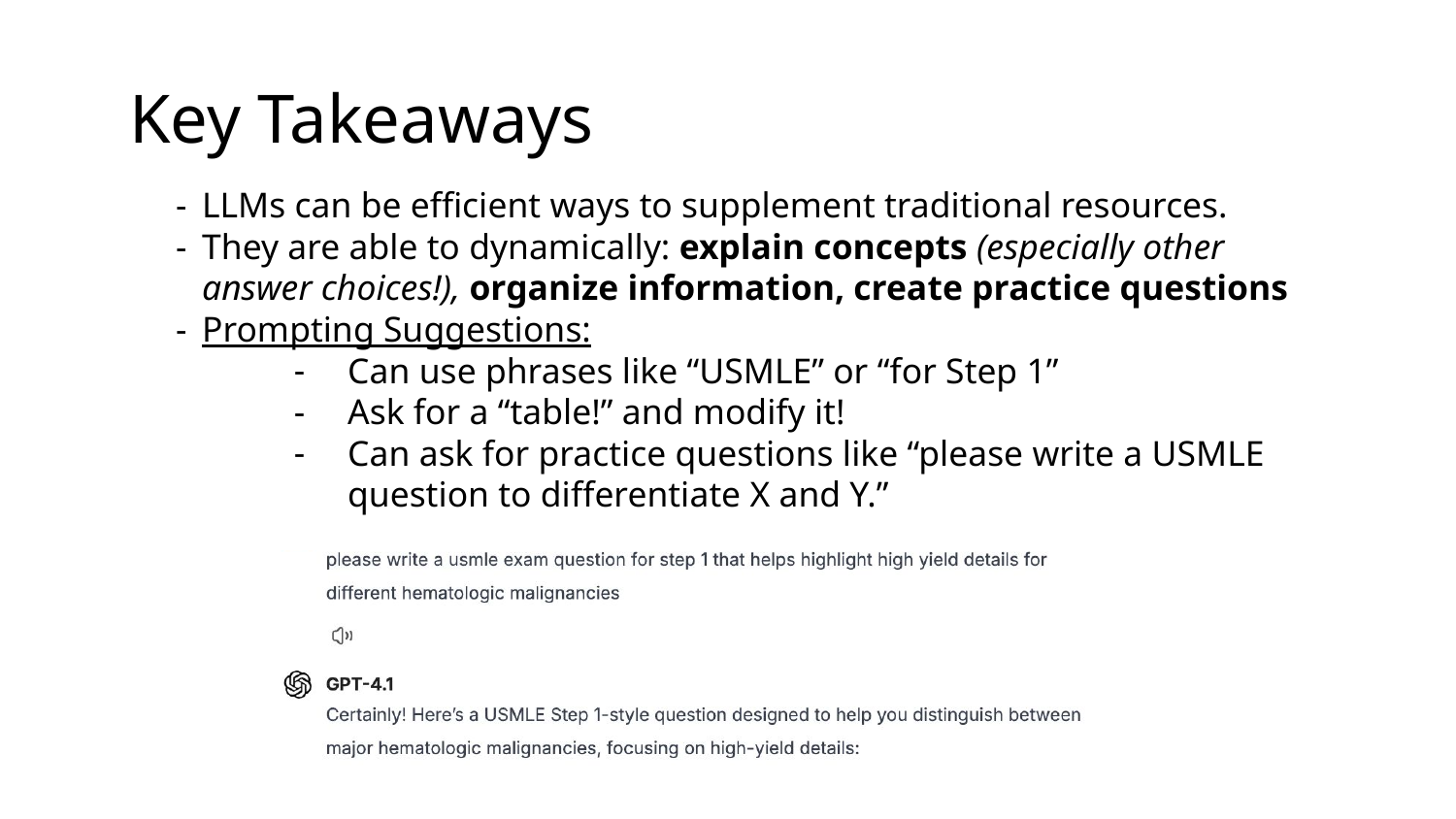

# Key Takeaways
LLMs can be efficient ways to supplement traditional resources.
They are able to dynamically: explain concepts (especially other answer choices!), organize information, create practice questions
Prompting Suggestions:
Can use phrases like “USMLE” or “for Step 1”
Ask for a “table!” and modify it!
Can ask for practice questions like “please write a USMLE question to differentiate X and Y.”

## Slide 8
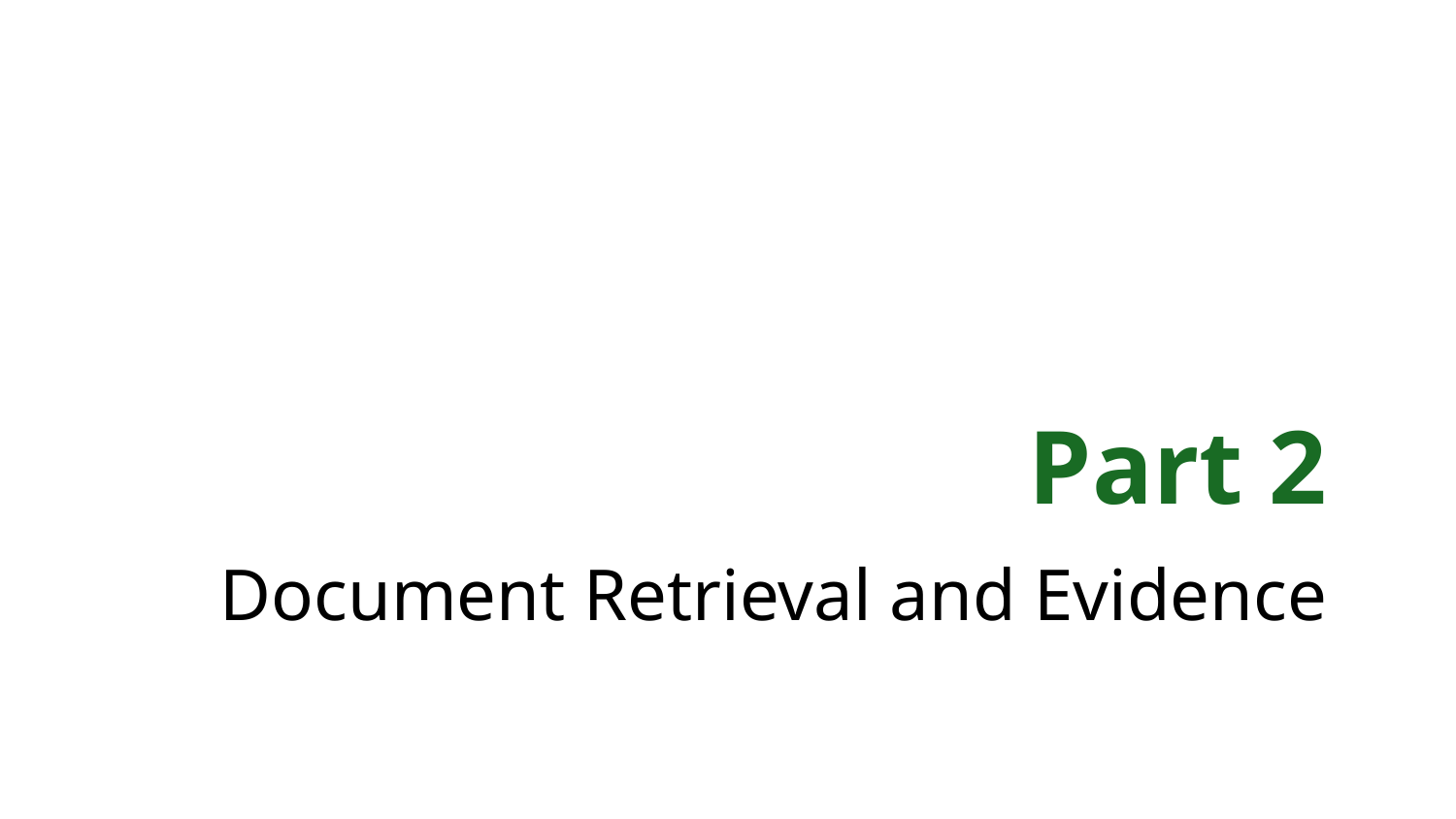

Part 2
# Document Retrieval and Evidence

## Slide 9
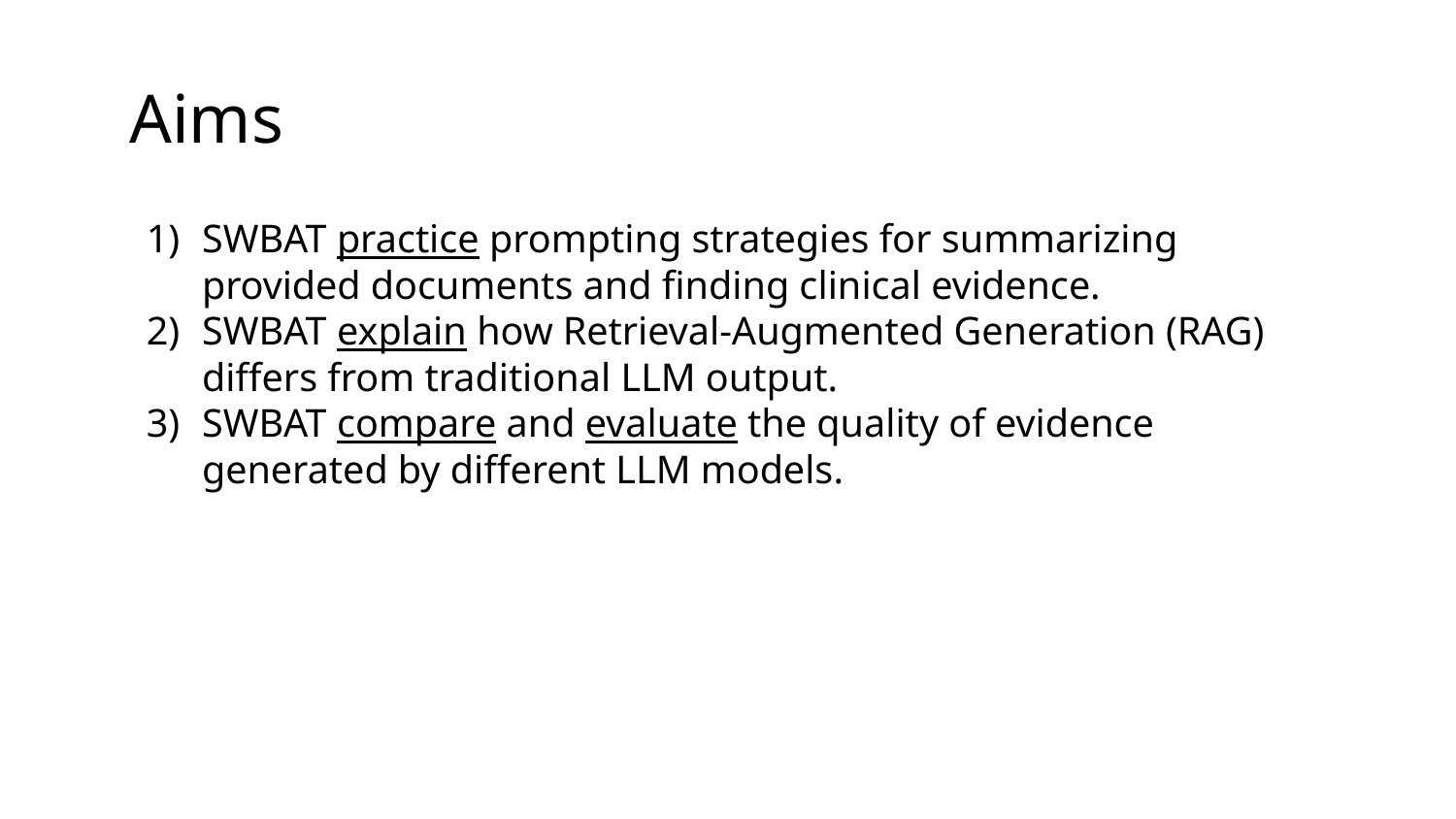

# Aims
SWBAT practice prompting strategies for summarizing provided documents and finding clinical evidence.
SWBAT explain how Retrieval-Augmented Generation (RAG) differs from traditional LLM output.
SWBAT compare and evaluate the quality of evidence generated by different LLM models.

## Slide 10
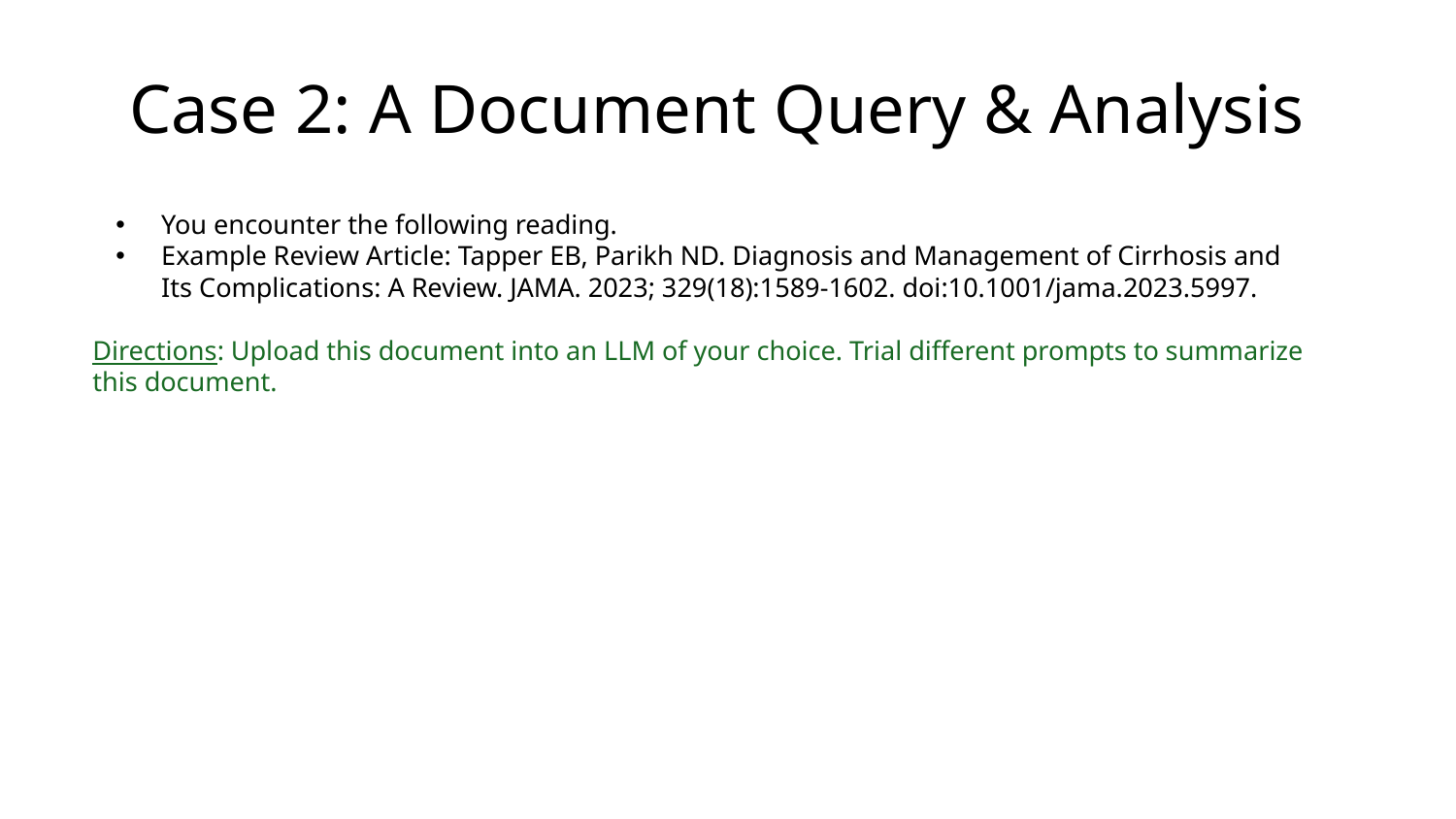

# Case 2: A Document Query & Analysis
You encounter the following reading.
Example Review Article: Tapper EB, Parikh ND. Diagnosis and Management of Cirrhosis and Its Complications: A Review. JAMA. 2023; 329(18):1589-1602. doi:10.1001/jama.2023.5997.
Directions: Upload this document into an LLM of your choice. Trial different prompts to summarize this document.

## Slide 11
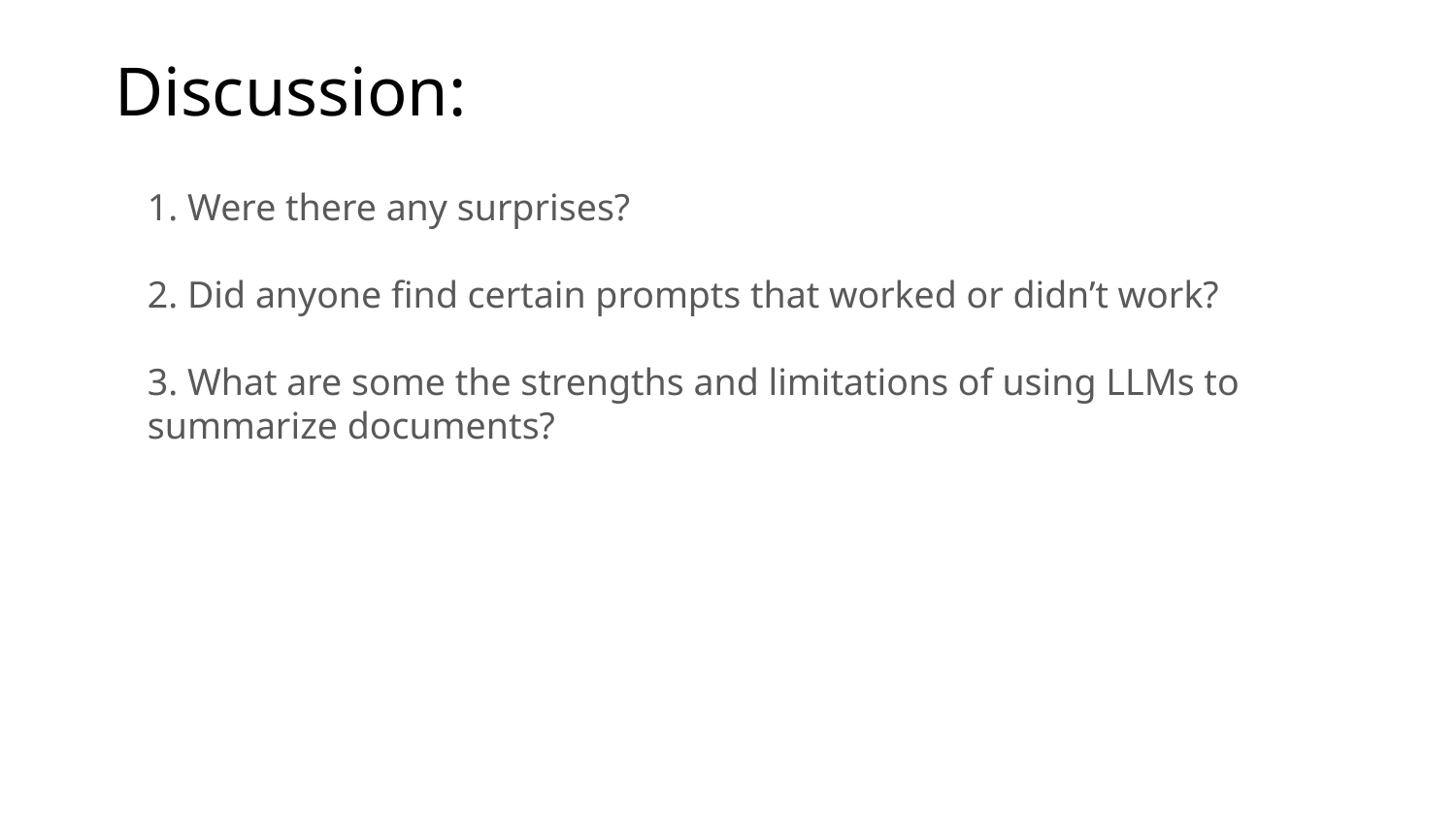

# Discussion:
1. Were there any surprises?
2. Did anyone find certain prompts that worked or didn’t work?
3. What are some the strengths and limitations of using LLMs to summarize documents?

## Slide 12
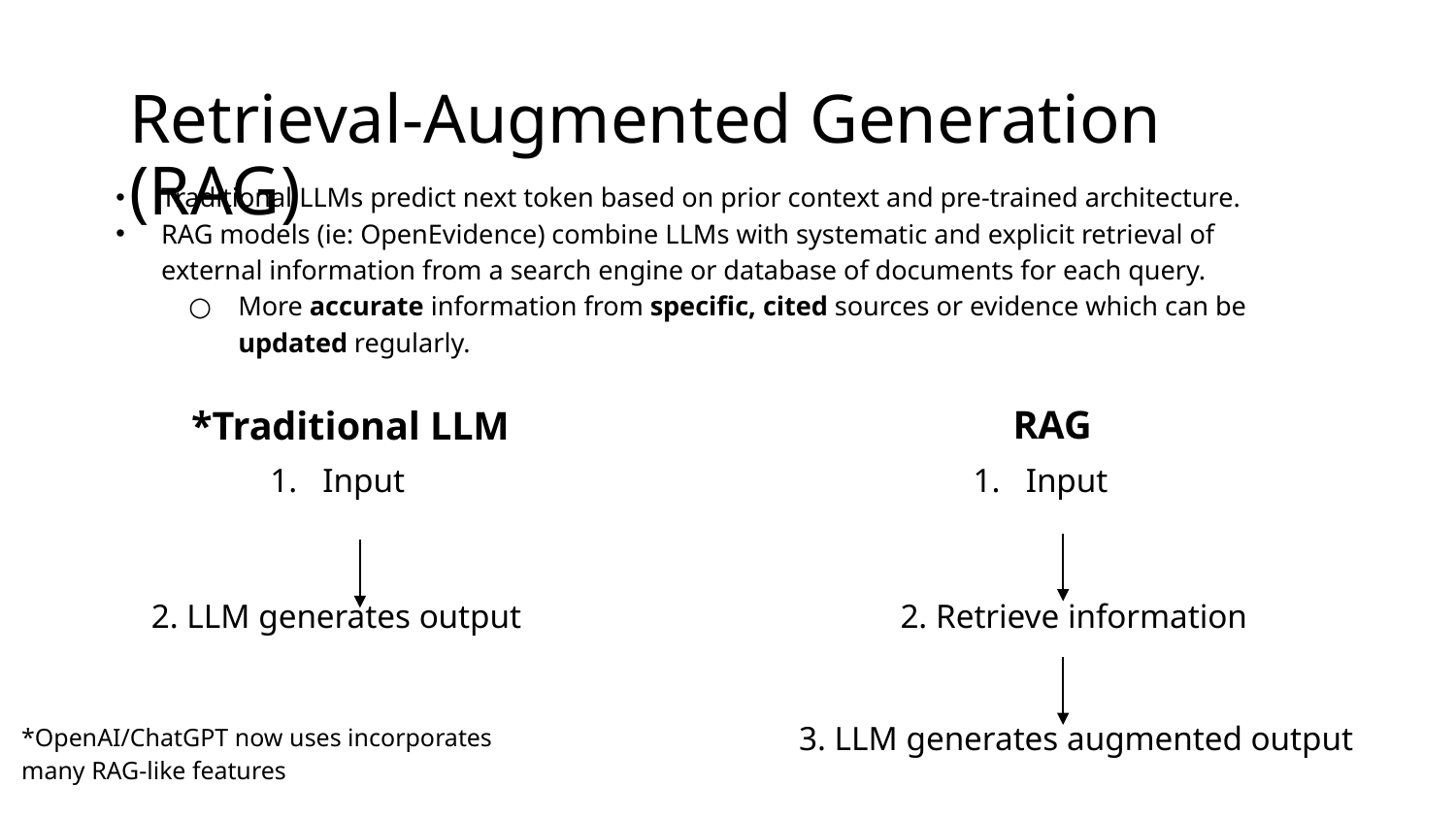

# Retrieval-Augmented Generation (RAG)
Traditional LLMs predict next token based on prior context and pre-trained architecture.
RAG models (ie: OpenEvidence) combine LLMs with systematic and explicit retrieval of external information from a search engine or database of documents for each query.
More accurate information from specific, cited sources or evidence which can be updated regularly.
RAG
*Traditional LLM
Input
Input
2. LLM generates output
2. Retrieve information
3. LLM generates augmented output
*OpenAI/ChatGPT now uses incorporates many RAG-like features

## Slide 13
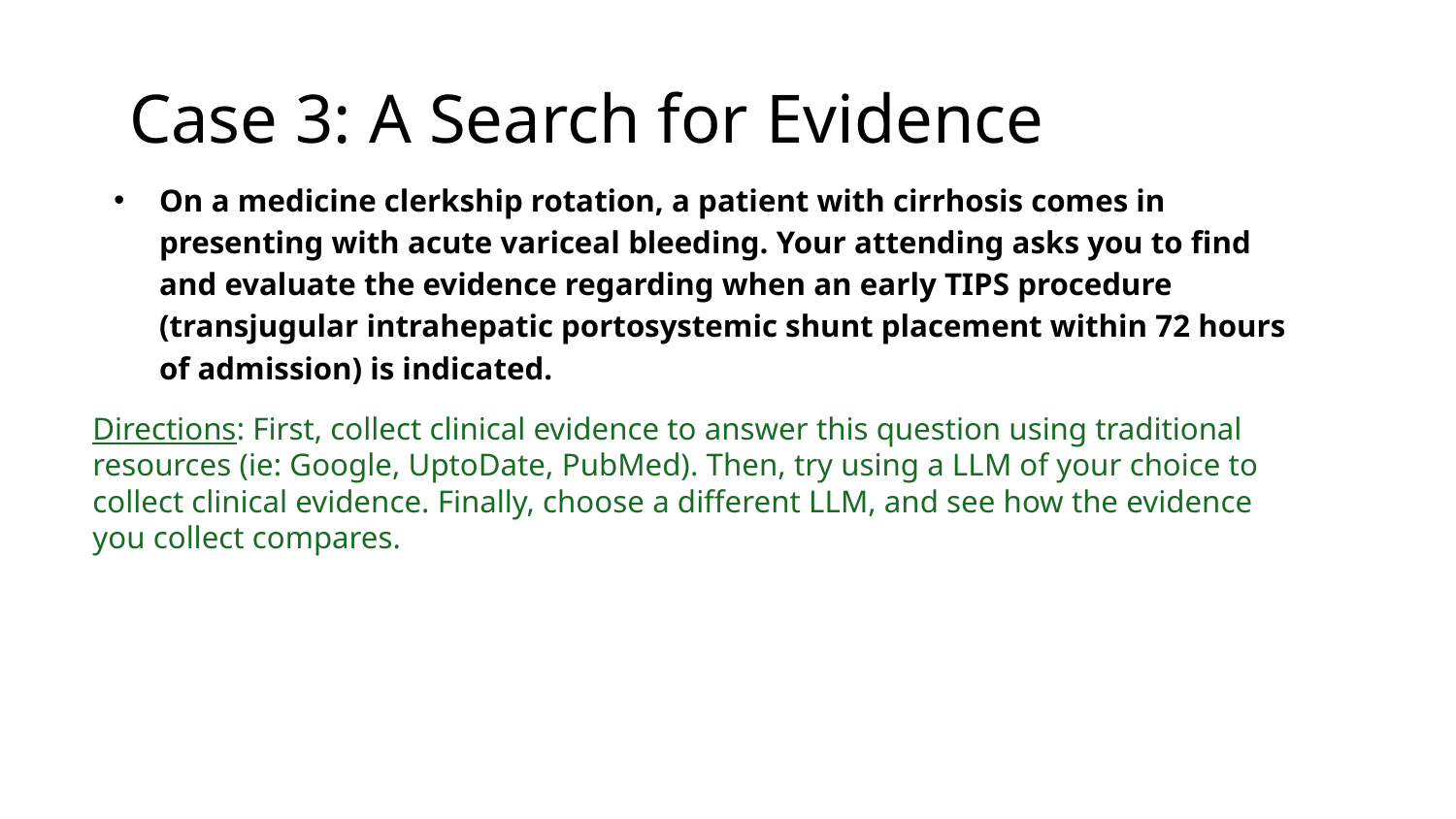

# Case 3: A Search for Evidence
On a medicine clerkship rotation, a patient with cirrhosis comes in presenting with acute variceal bleeding. Your attending asks you to find and evaluate the evidence regarding when an early TIPS procedure (transjugular intrahepatic portosystemic shunt placement within 72 hours of admission) is indicated.
Directions: First, collect clinical evidence to answer this question using traditional resources (ie: Google, UptoDate, PubMed). Then, try using a LLM of your choice to collect clinical evidence. Finally, choose a different LLM, and see how the evidence you collect compares.

## Slide 14
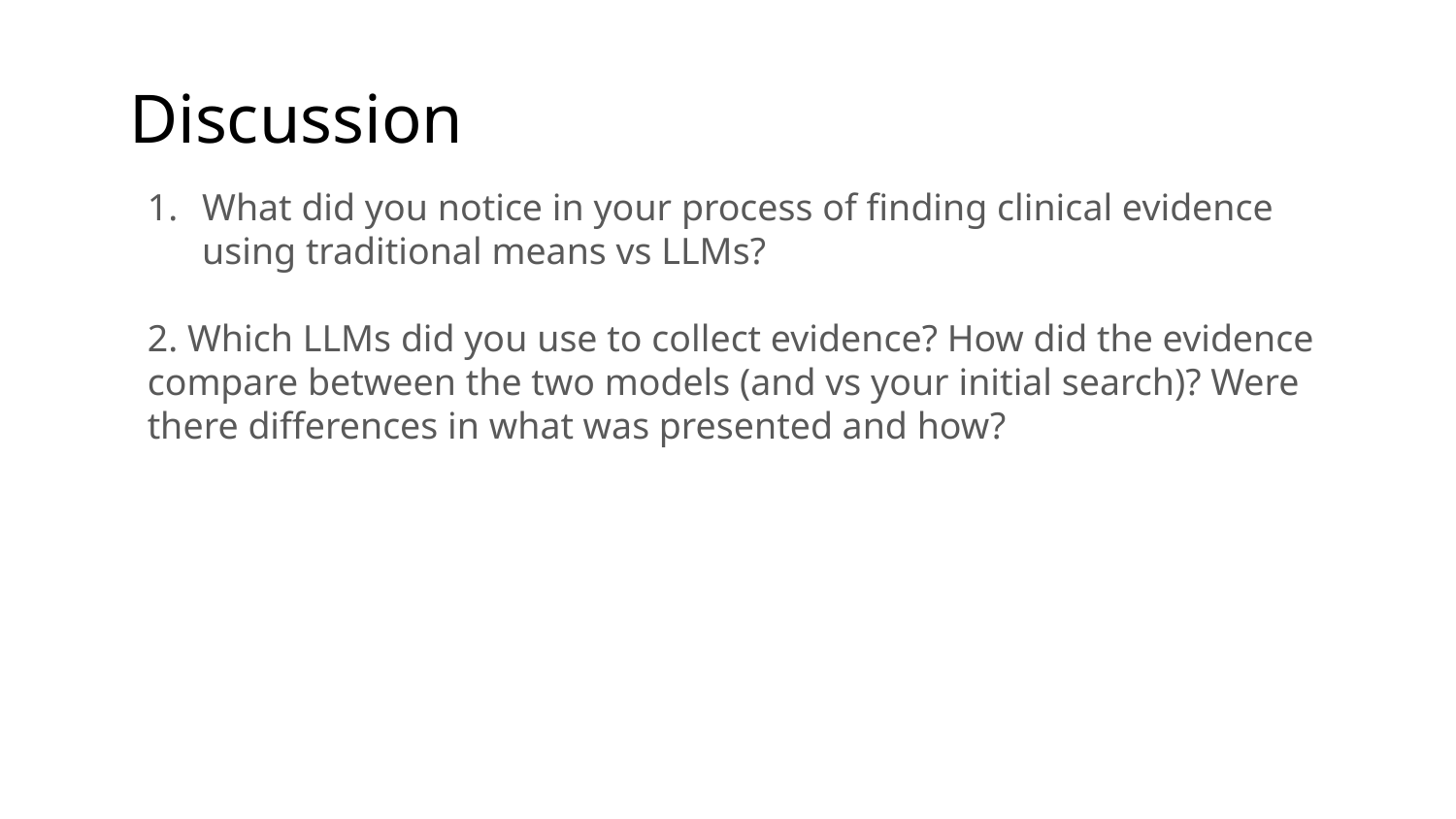

# Discussion
What did you notice in your process of finding clinical evidence using traditional means vs LLMs?
2. Which LLMs did you use to collect evidence? How did the evidence compare between the two models (and vs your initial search)? Were there differences in what was presented and how?

## Slide 15
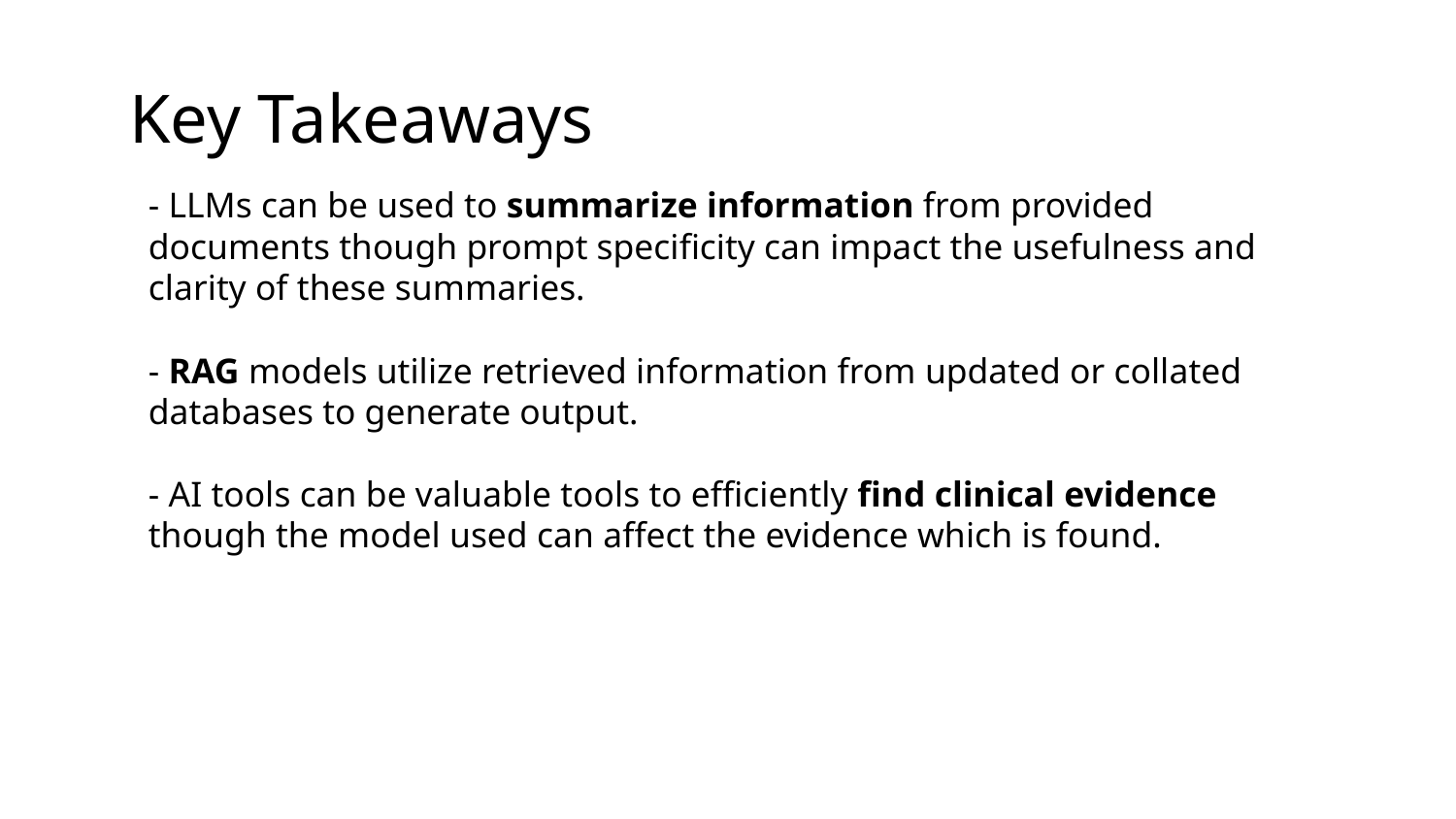

# Key Takeaways
- LLMs can be used to summarize information from provided documents though prompt specificity can impact the usefulness and clarity of these summaries.
- RAG models utilize retrieved information from updated or collated databases to generate output.
- AI tools can be valuable tools to efficiently find clinical evidence though the model used can affect the evidence which is found.

## Slide 16
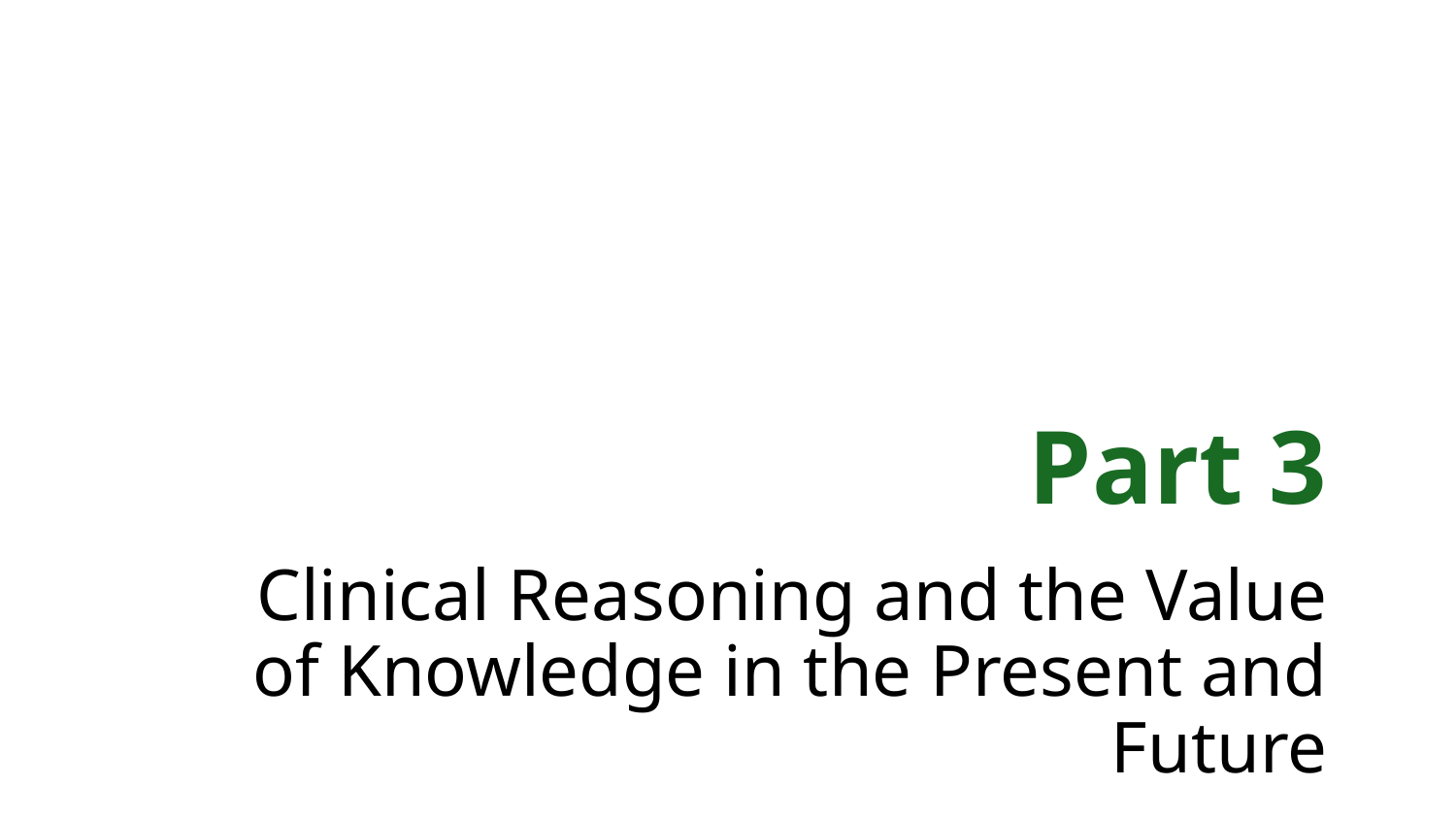

Part 3
# Clinical Reasoning and the Value of Knowledge in the Present and Future

## Slide 17
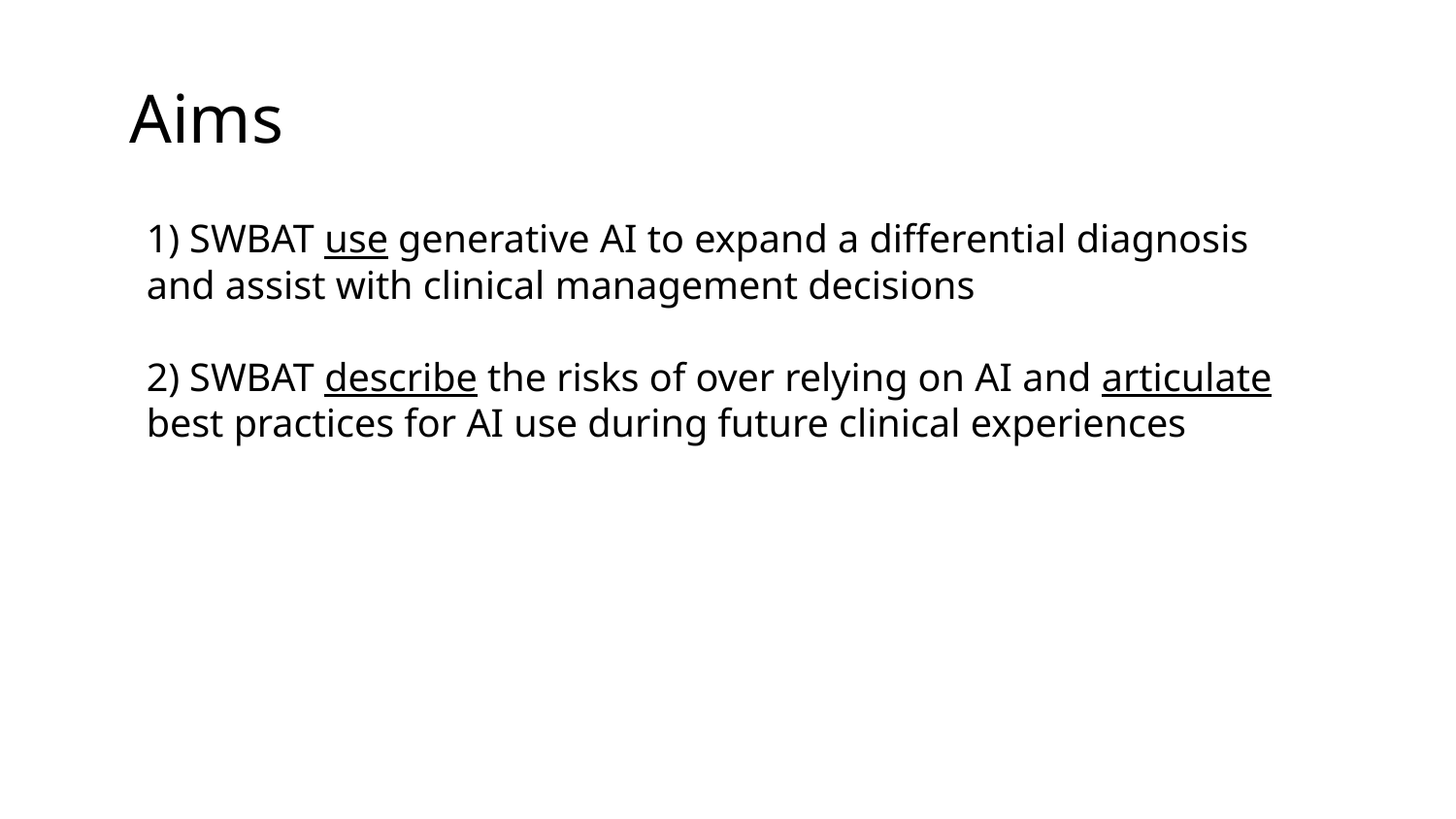

# Aims
1) SWBAT use generative AI to expand a differential diagnosis and assist with clinical management decisions
2) SWBAT describe the risks of over relying on AI and articulate best practices for AI use during future clinical experiences

## Slide 18
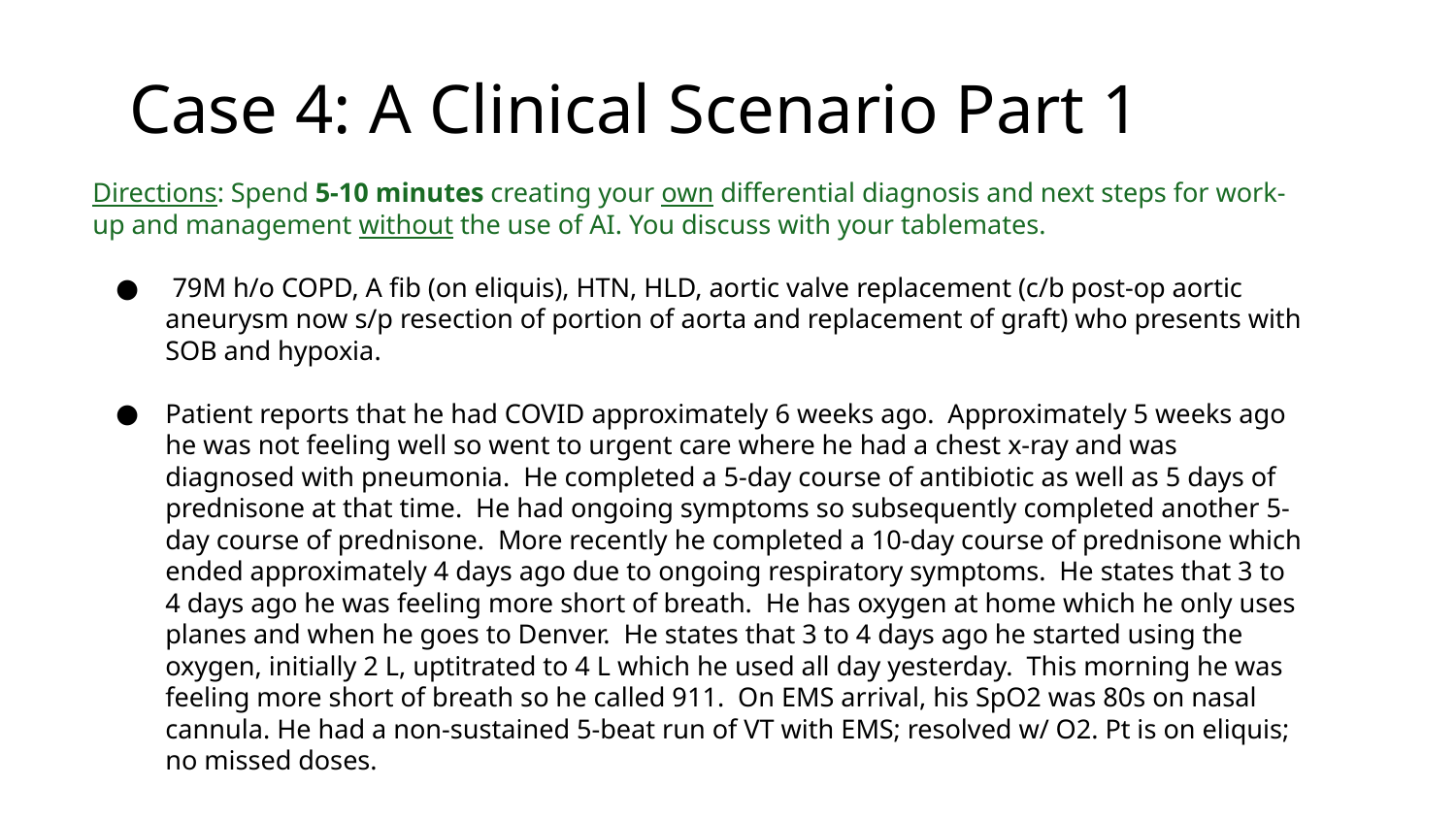

# Case 4: A Clinical Scenario Part 1
Directions: Spend 5-10 minutes creating your own differential diagnosis and next steps for work-up and management without the use of AI. You discuss with your tablemates.
 79M h/o COPD, A fib (on eliquis), HTN, HLD, aortic valve replacement (c/b post-op aortic aneurysm now s/p resection of portion of aorta and replacement of graft) who presents with SOB and hypoxia.
Patient reports that he had COVID approximately 6 weeks ago. Approximately 5 weeks ago he was not feeling well so went to urgent care where he had a chest x-ray and was diagnosed with pneumonia. He completed a 5-day course of antibiotic as well as 5 days of prednisone at that time. He had ongoing symptoms so subsequently completed another 5-day course of prednisone. More recently he completed a 10-day course of prednisone which ended approximately 4 days ago due to ongoing respiratory symptoms. He states that 3 to 4 days ago he was feeling more short of breath. He has oxygen at home which he only uses planes and when he goes to Denver. He states that 3 to 4 days ago he started using the oxygen, initially 2 L, uptitrated to 4 L which he used all day yesterday. This morning he was feeling more short of breath so he called 911. On EMS arrival, his SpO2 was 80s on nasal cannula. He had a non-sustained 5-beat run of VT with EMS; resolved w/ O2. Pt is on eliquis; no missed doses.

## Slide 19
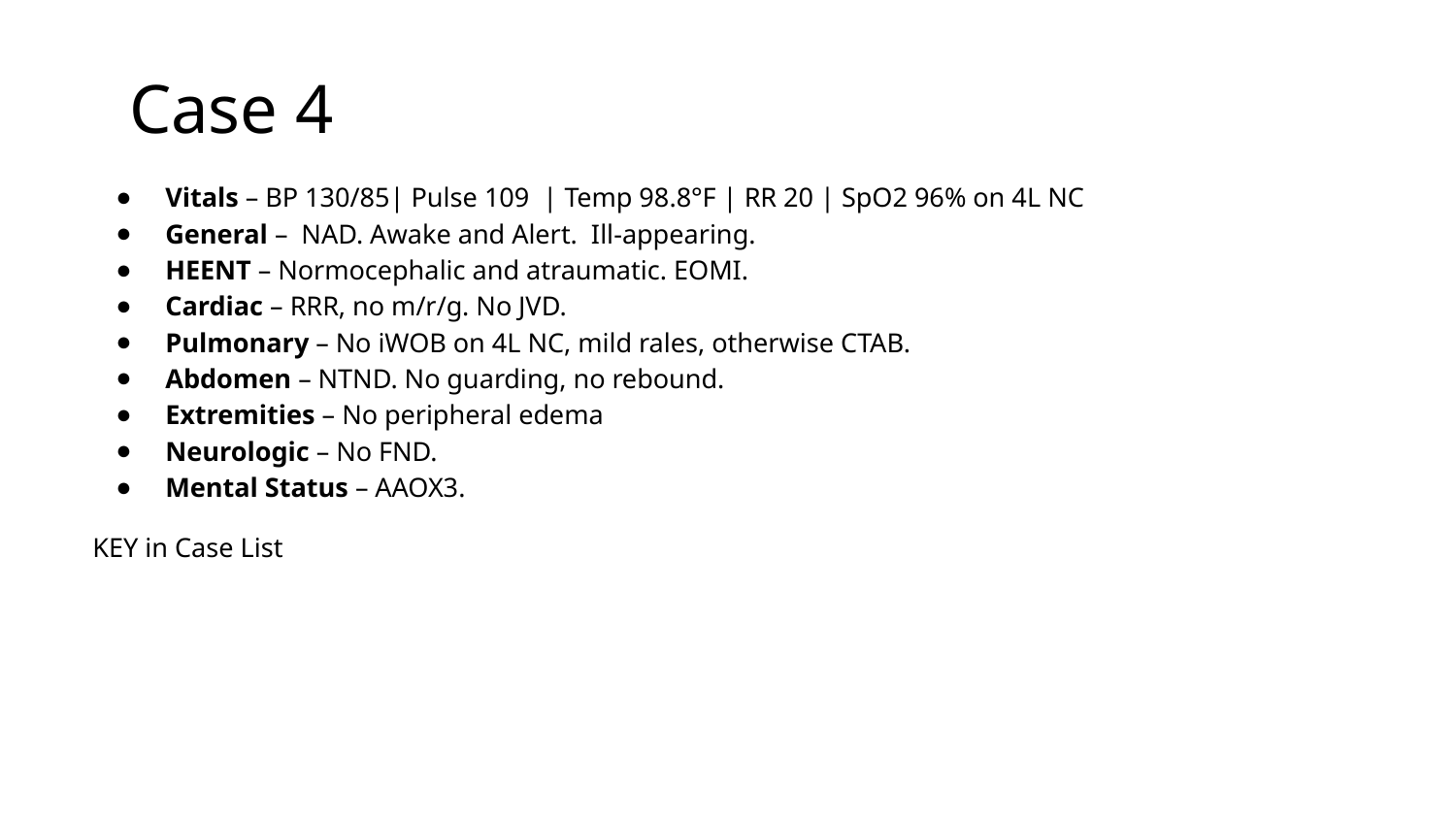

# Case 4
Vitals – BP 130/85| Pulse 109 | Temp 98.8°F | RR 20 | SpO2 96% on 4L NC
General – NAD. Awake and Alert. Ill-appearing.
HEENT – Normocephalic and atraumatic. EOMI.
Cardiac – RRR, no m/r/g. No JVD.
Pulmonary – No iWOB on 4L NC, mild rales, otherwise CTAB.
Abdomen – NTND. No guarding, no rebound.
Extremities – No peripheral edema
Neurologic – No FND.
Mental Status – AAOX3.
KEY in Case List

## Slide 20
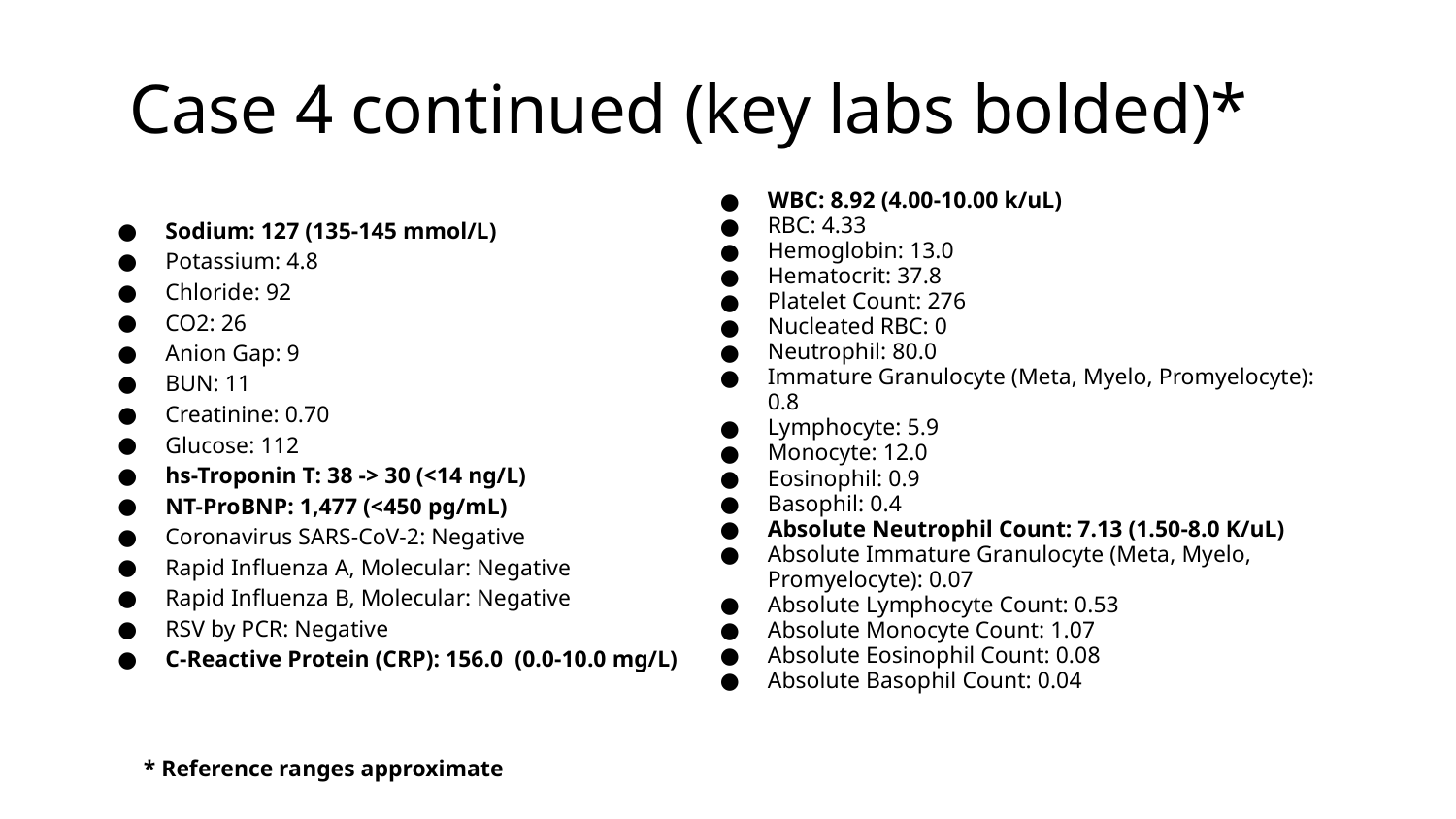

# Case 4 continued (key labs bolded)*
WBC: 8.92 (4.00-10.00 k/uL)
RBC: 4.33
Hemoglobin: 13.0
Hematocrit: 37.8
Platelet Count: 276
Nucleated RBC: 0
Neutrophil: 80.0
Immature Granulocyte (Meta, Myelo, Promyelocyte): 0.8
Lymphocyte: 5.9
Monocyte: 12.0
Eosinophil: 0.9
Basophil: 0.4
Absolute Neutrophil Count: 7.13 (1.50-8.0 K/uL)
Absolute Immature Granulocyte (Meta, Myelo, Promyelocyte): 0.07
Absolute Lymphocyte Count: 0.53
Absolute Monocyte Count: 1.07
Absolute Eosinophil Count: 0.08
Absolute Basophil Count: 0.04
Sodium: 127 (135-145 mmol/L)
Potassium: 4.8
Chloride: 92
CO2: 26
Anion Gap: 9
BUN: 11
Creatinine: 0.70
Glucose: 112
hs-Troponin T: 38 -> 30 (<14 ng/L)
NT-ProBNP: 1,477 (<450 pg/mL)
Coronavirus SARS-CoV-2: Negative
Rapid Influenza A, Molecular: Negative
Rapid Influenza B, Molecular: Negative
RSV by PCR: Negative
C-Reactive Protein (CRP): 156.0 (0.0-10.0 mg/L)
* Reference ranges approximate

## Slide 21
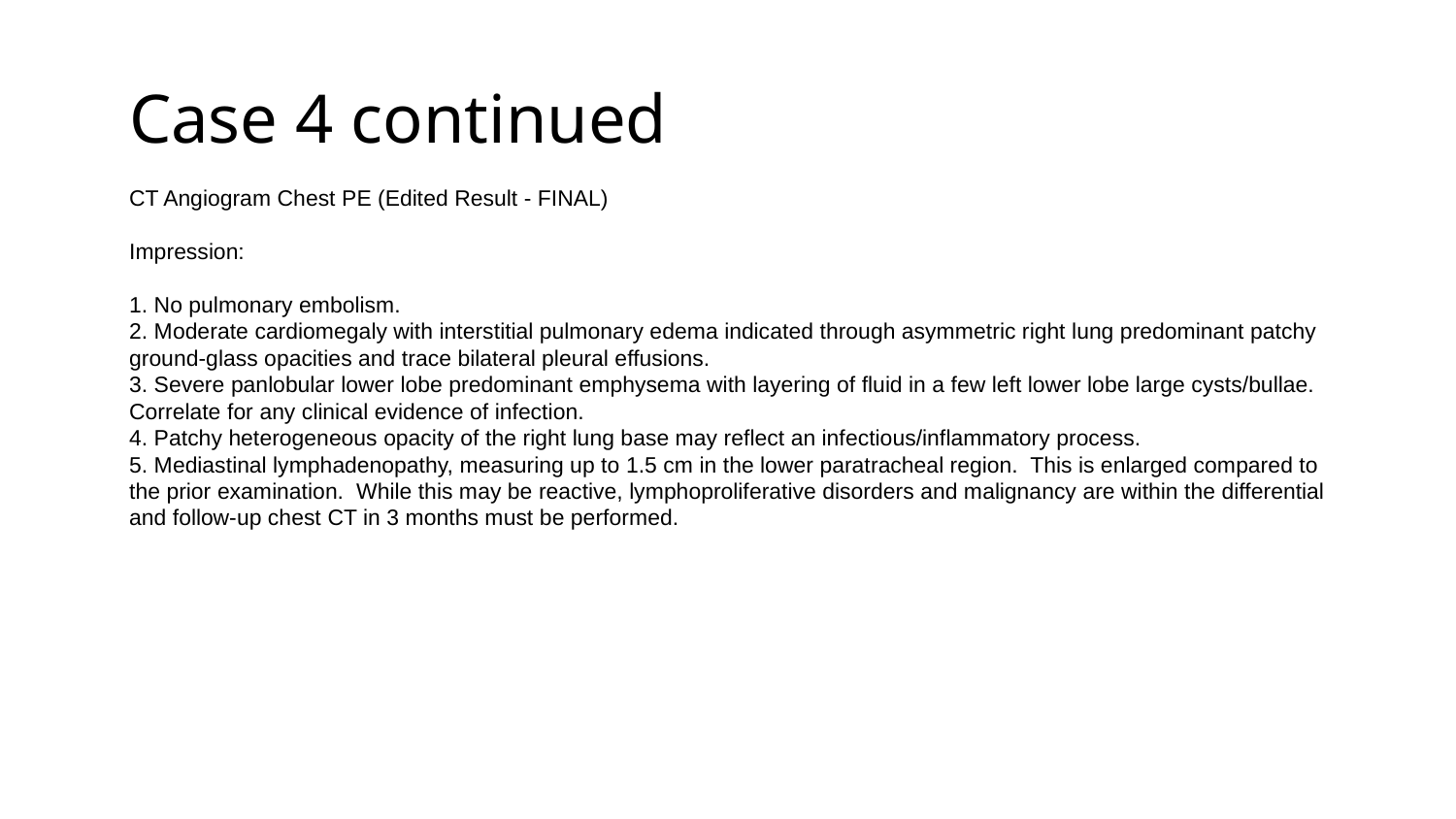

# Case 4 continued
CT Angiogram Chest PE (Edited Result - FINAL)
Impression:
1. No pulmonary embolism.
2. Moderate cardiomegaly with interstitial pulmonary edema indicated through asymmetric right lung predominant patchy ground-glass opacities and trace bilateral pleural effusions.
3. Severe panlobular lower lobe predominant emphysema with layering of fluid in a few left lower lobe large cysts/bullae. Correlate for any clinical evidence of infection.
4. Patchy heterogeneous opacity of the right lung base may reflect an infectious/inflammatory process.
5. Mediastinal lymphadenopathy, measuring up to 1.5 cm in the lower paratracheal region. This is enlarged compared to the prior examination. While this may be reactive, lymphoproliferative disorders and malignancy are within the differential and follow-up chest CT in 3 months must be performed.

## Slide 22
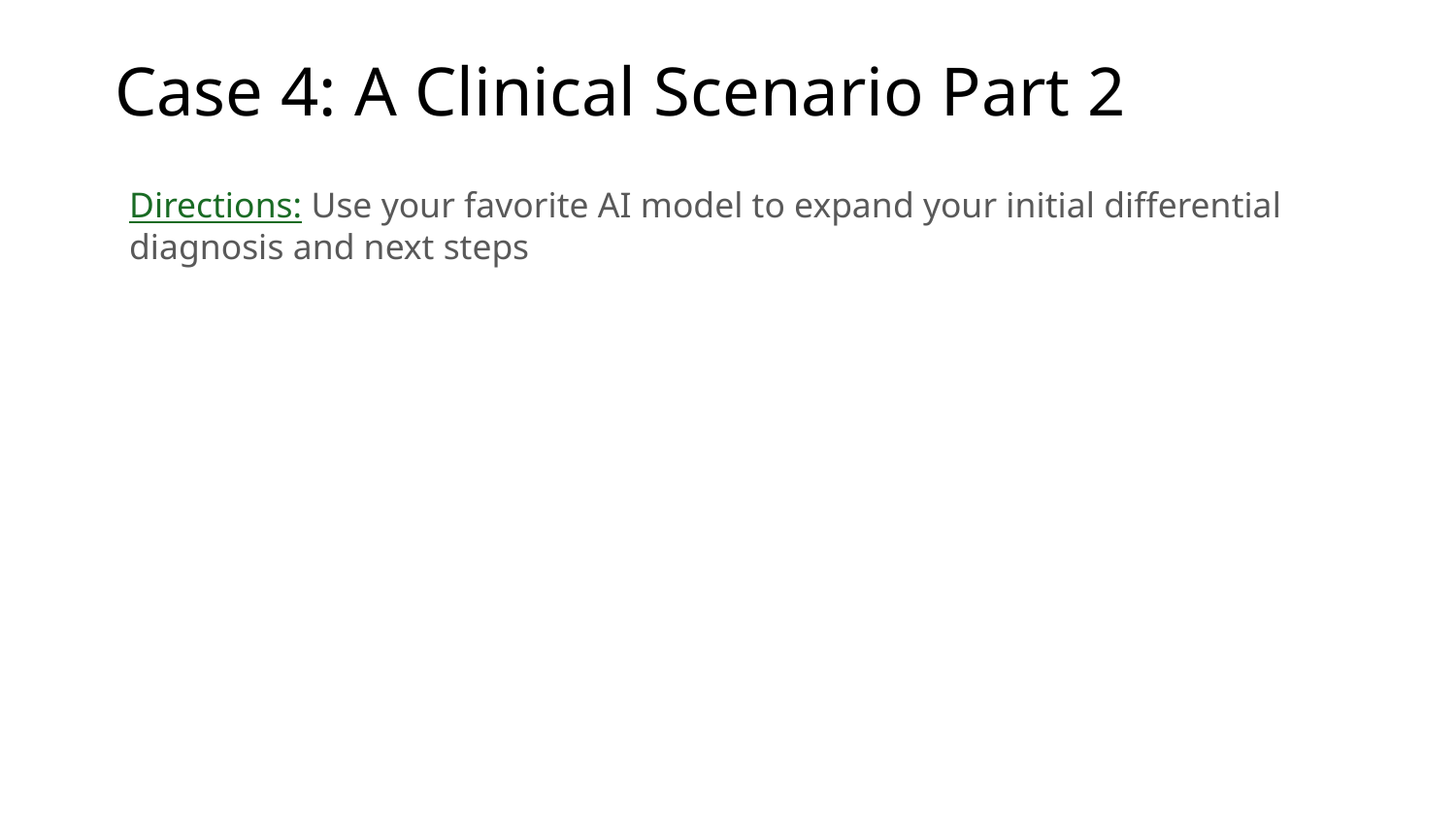

# Case 4: A Clinical Scenario Part 2
Directions: Use your favorite AI model to expand your initial differential diagnosis and next steps

## Slide 23
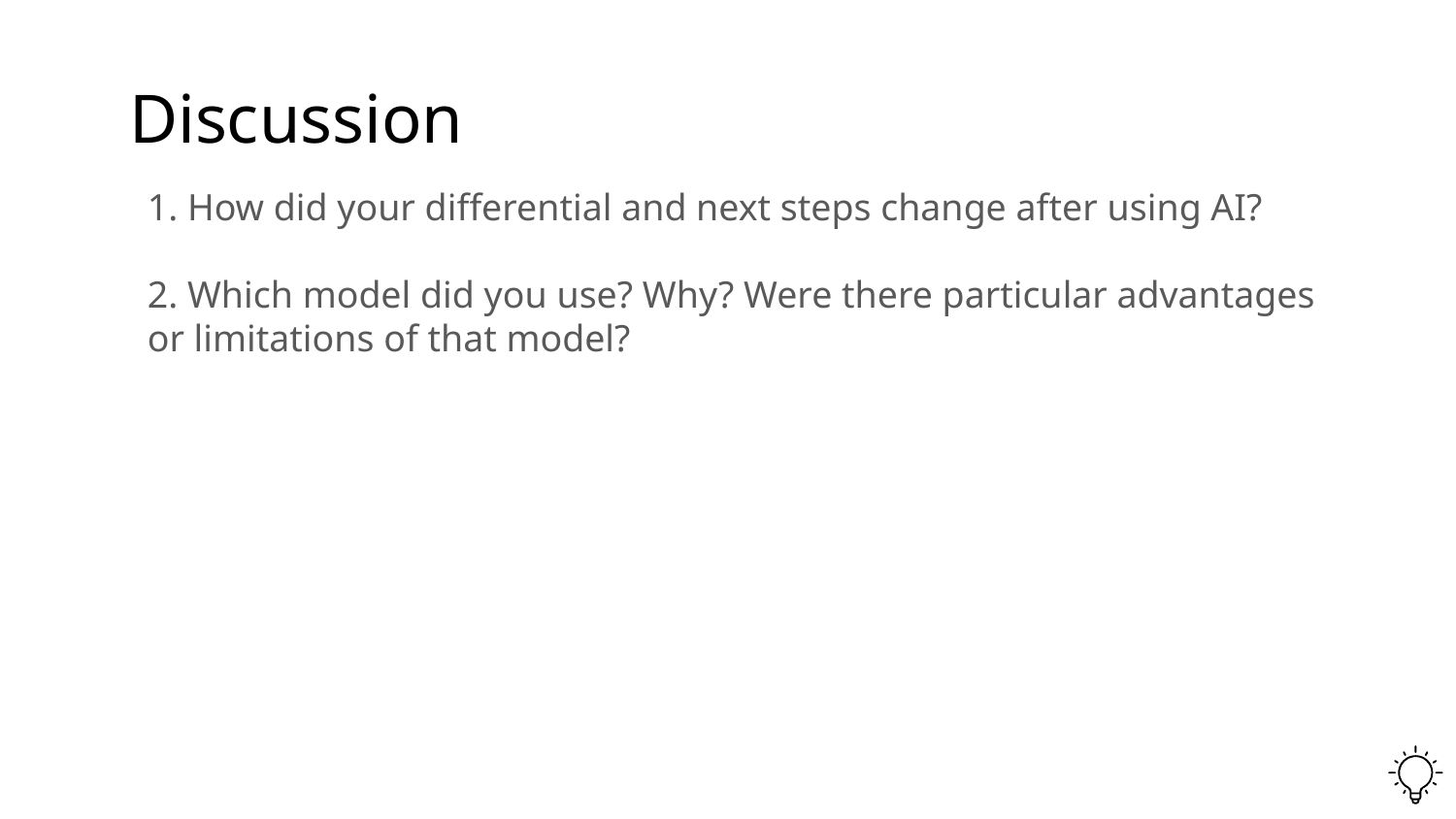

# Discussion
1. How did your differential and next steps change after using AI?
2. Which model did you use? Why? Were there particular advantages or limitations of that model?

## Slide 24
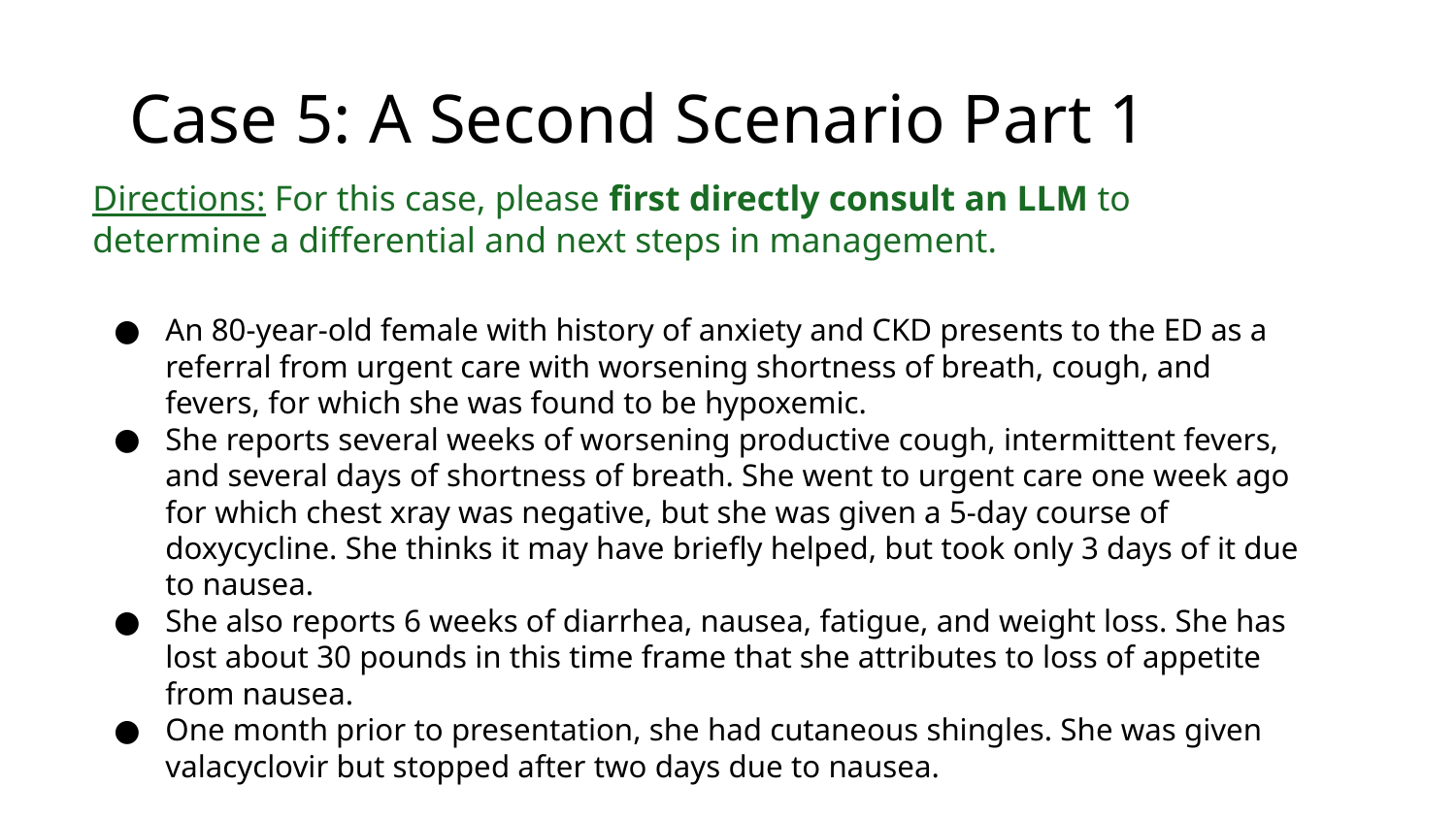

# Case 5: A Second Scenario Part 1
Directions: For this case, please first directly consult an LLM to determine a differential and next steps in management.
An 80-year-old female with history of anxiety and CKD presents to the ED as a referral from urgent care with worsening shortness of breath, cough, and fevers, for which she was found to be hypoxemic.
She reports several weeks of worsening productive cough, intermittent fevers, and several days of shortness of breath. She went to urgent care one week ago for which chest xray was negative, but she was given a 5-day course of doxycycline. She thinks it may have briefly helped, but took only 3 days of it due to nausea.
She also reports 6 weeks of diarrhea, nausea, fatigue, and weight loss. She has lost about 30 pounds in this time frame that she attributes to loss of appetite from nausea.
One month prior to presentation, she had cutaneous shingles. She was given valacyclovir but stopped after two days due to nausea.

## Slide 25
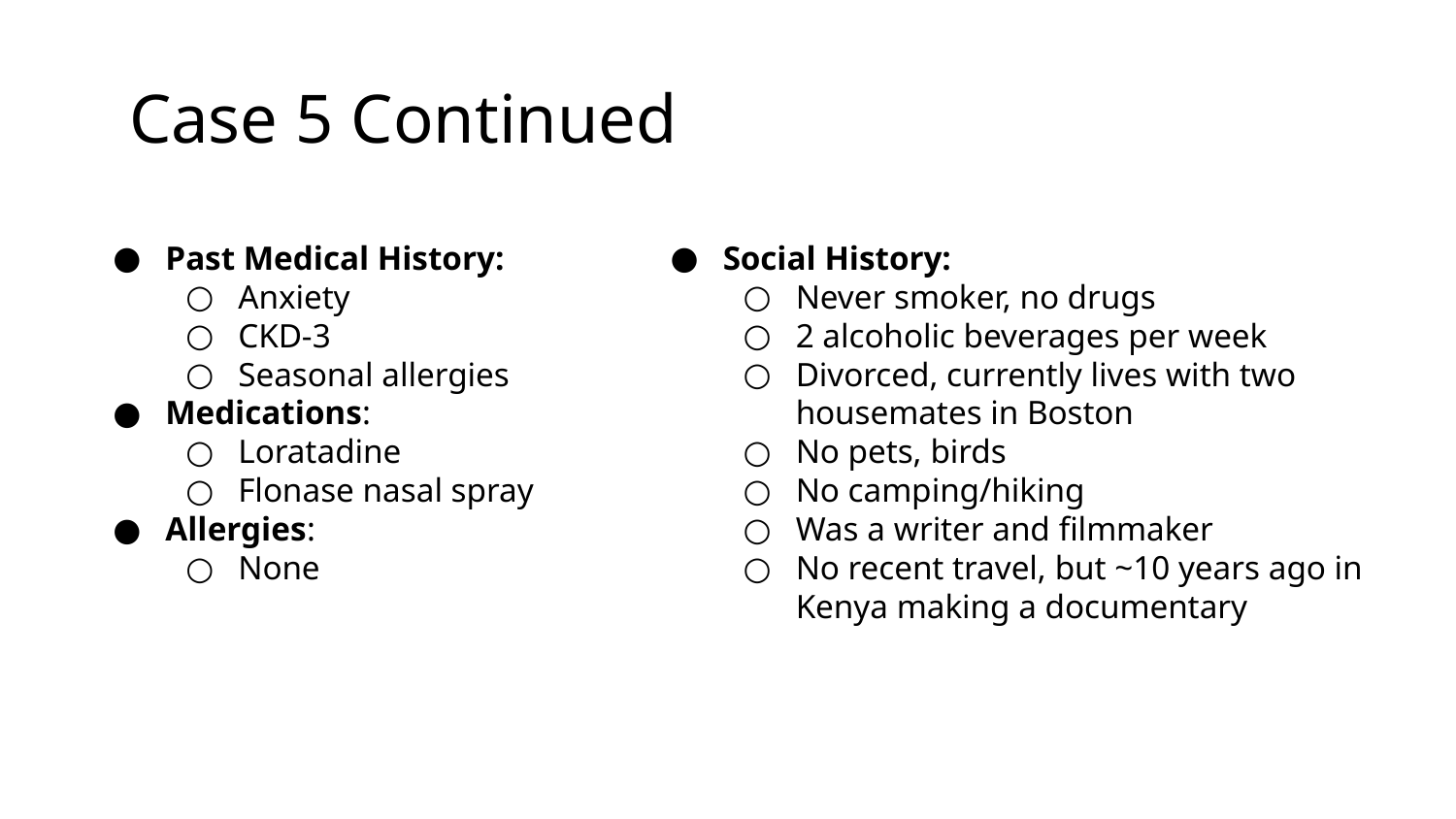

# Case 5 Continued
Past Medical History:
Anxiety
CKD-3
Seasonal allergies
Medications:
Loratadine
Flonase nasal spray
Allergies:
None
Social History:
Never smoker, no drugs
2 alcoholic beverages per week
Divorced, currently lives with two housemates in Boston
No pets, birds
No camping/hiking
Was a writer and filmmaker
No recent travel, but ~10 years ago in Kenya making a documentary

## Slide 26
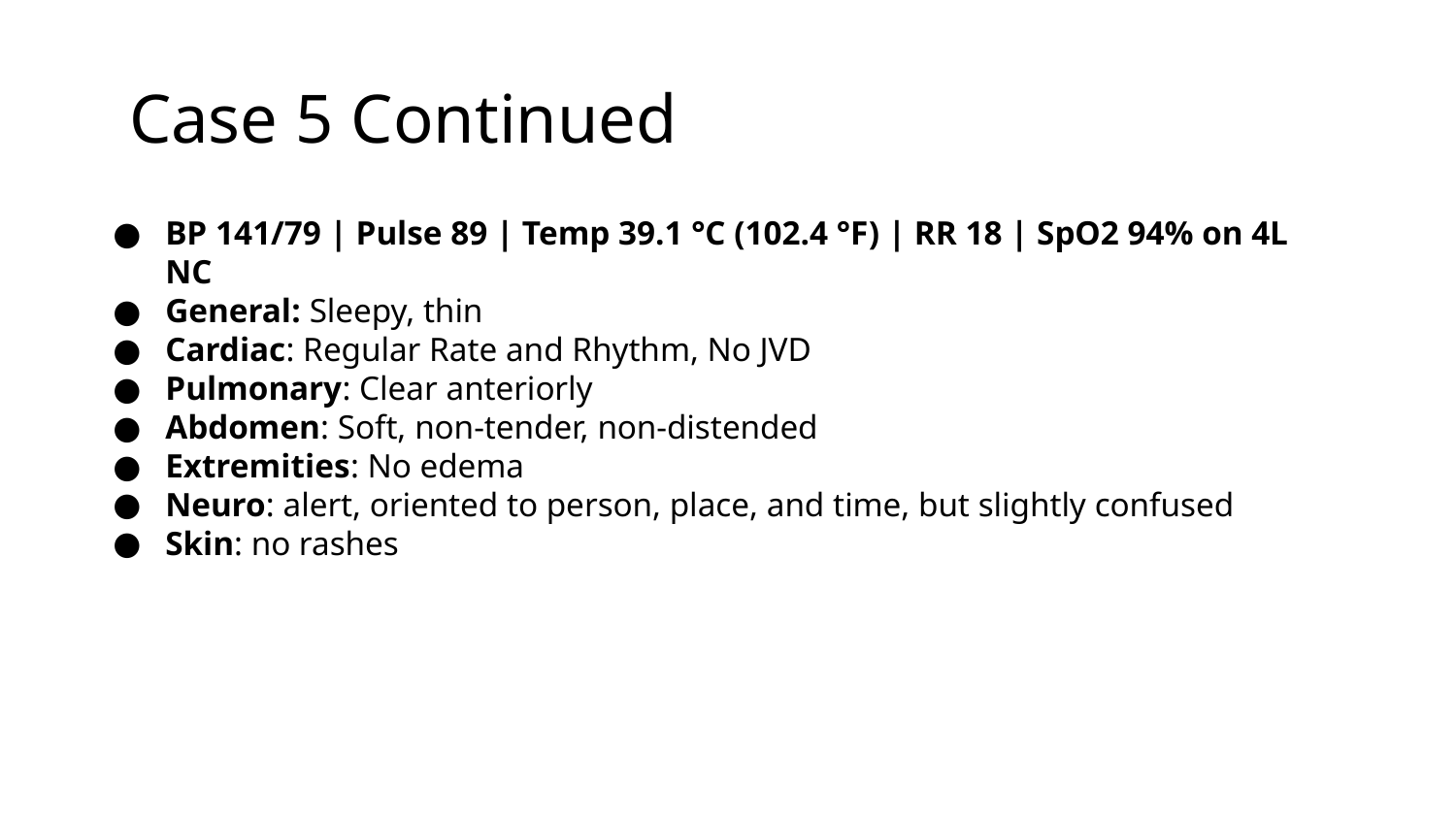

# Case 5 Continued
BP 141/79 | Pulse 89 | Temp 39.1 °C (102.4 °F) | RR 18 | SpO2 94% on 4L NC
General: Sleepy, thin
Cardiac: Regular Rate and Rhythm, No JVD
Pulmonary: Clear anteriorly
Abdomen: Soft, non-tender, non-distended
Extremities: No edema
Neuro: alert, oriented to person, place, and time, but slightly confused
Skin: no rashes

## Slide 27
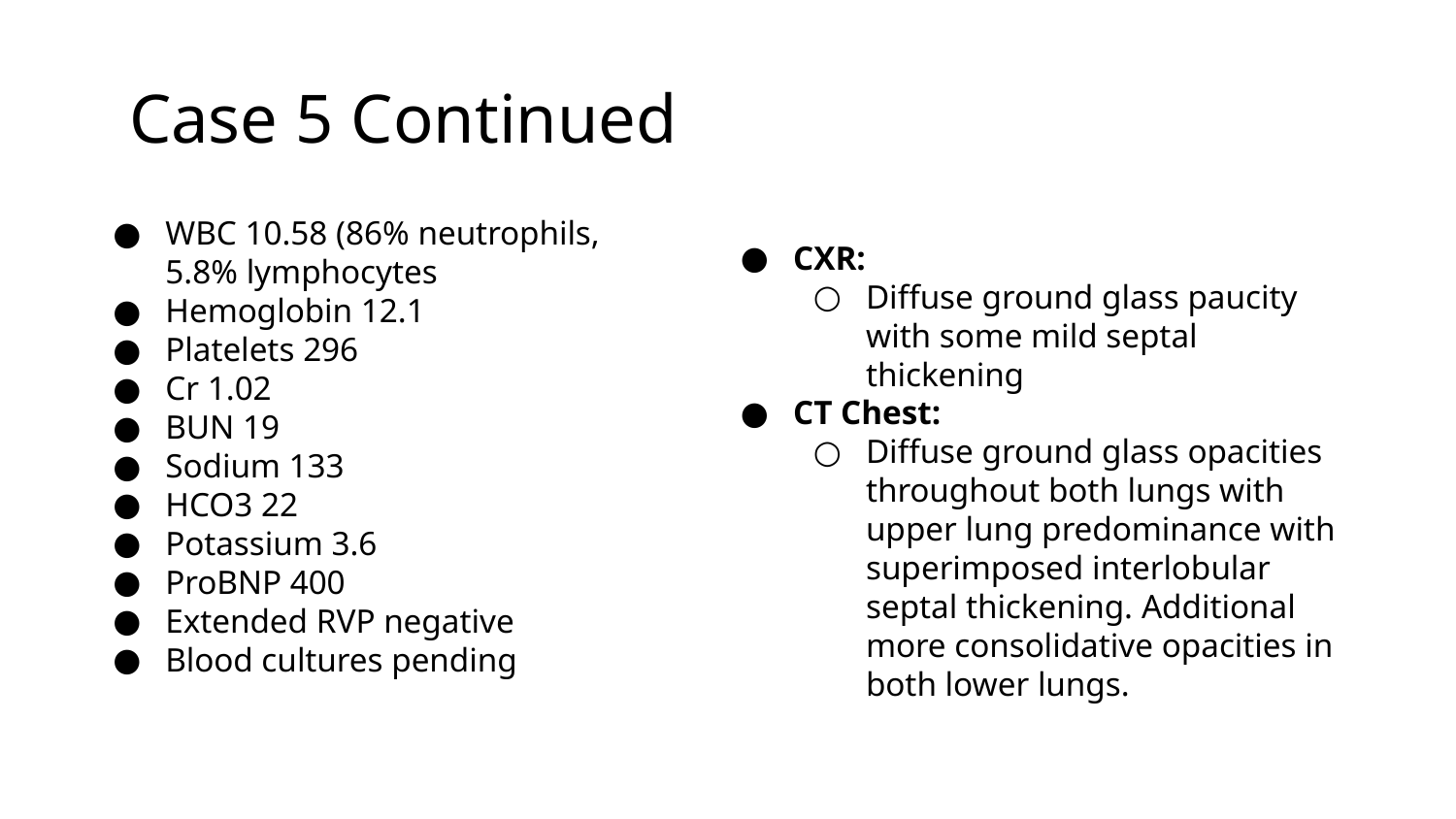

# Case 5 Continued
WBC 10.58 (86% neutrophils, 5.8% lymphocytes
Hemoglobin 12.1
Platelets 296
Cr 1.02
BUN 19
Sodium 133
HCO3 22
Potassium 3.6
ProBNP 400
Extended RVP negative
Blood cultures pending
CXR:
Diffuse ground glass paucity with some mild septal thickening
CT Chest:
Diffuse ground glass opacities throughout both lungs with upper lung predominance with superimposed interlobular septal thickening. Additional more consolidative opacities in both lower lungs.

## Slide 28
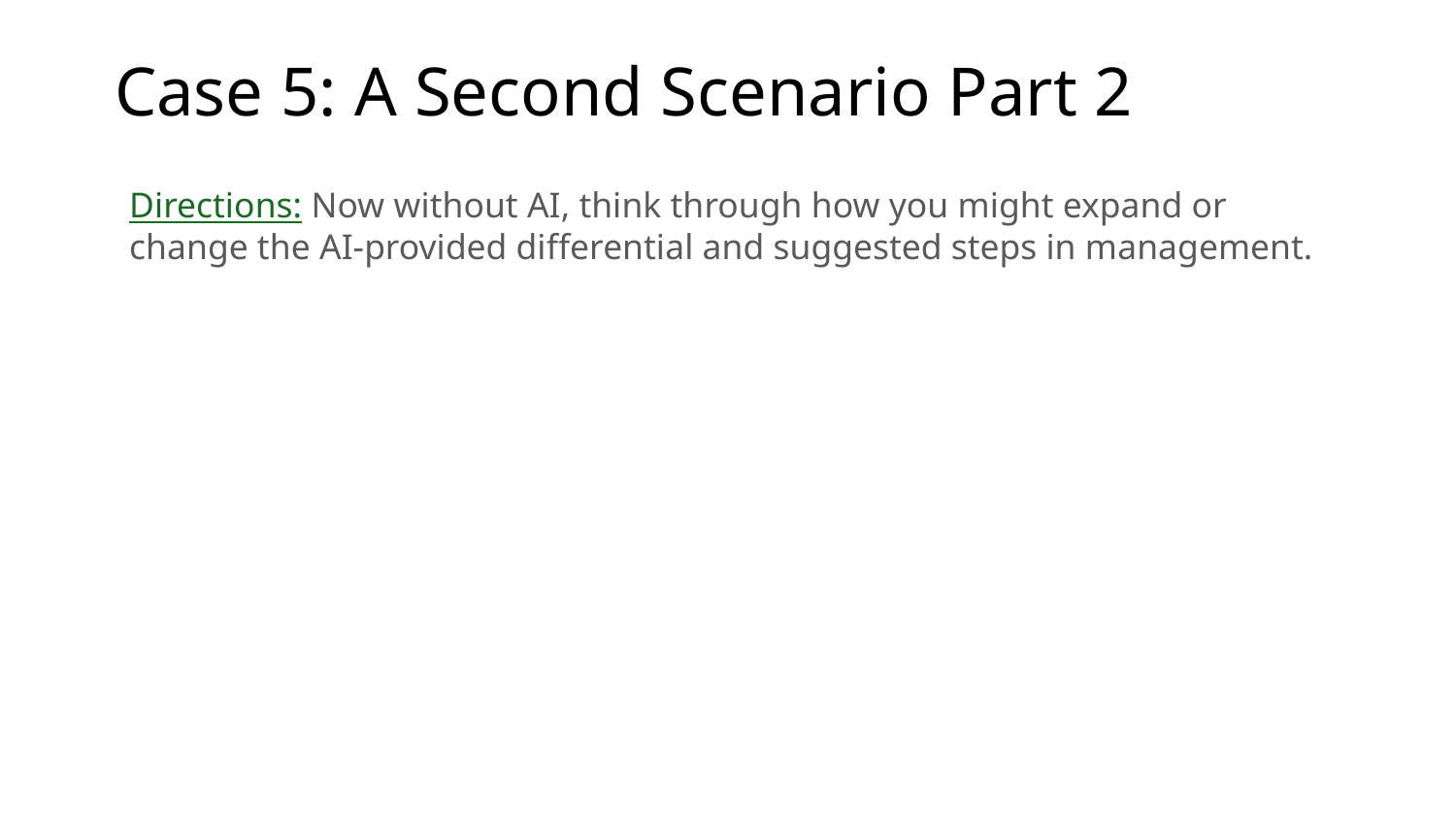

# Case 5: A Second Scenario Part 2
Directions: Now without AI, think through how you might expand or change the AI-provided differential and suggested steps in management.

## Slide 29
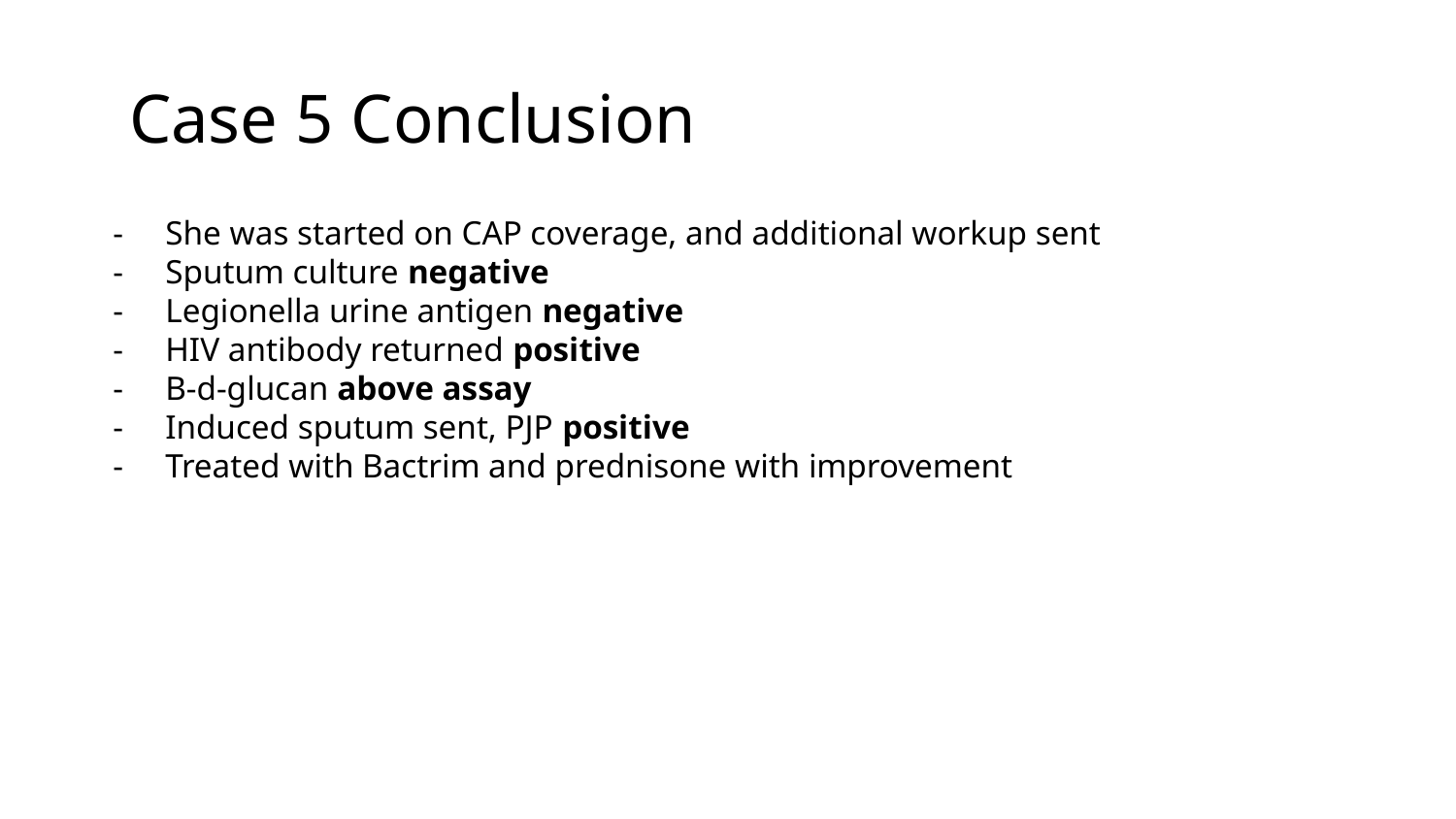

# Case 5 Conclusion
She was started on CAP coverage, and additional workup sent
Sputum culture negative
Legionella urine antigen negative
HIV antibody returned positive
B-d-glucan above assay
Induced sputum sent, PJP positive
Treated with Bactrim and prednisone with improvement

## Slide 30
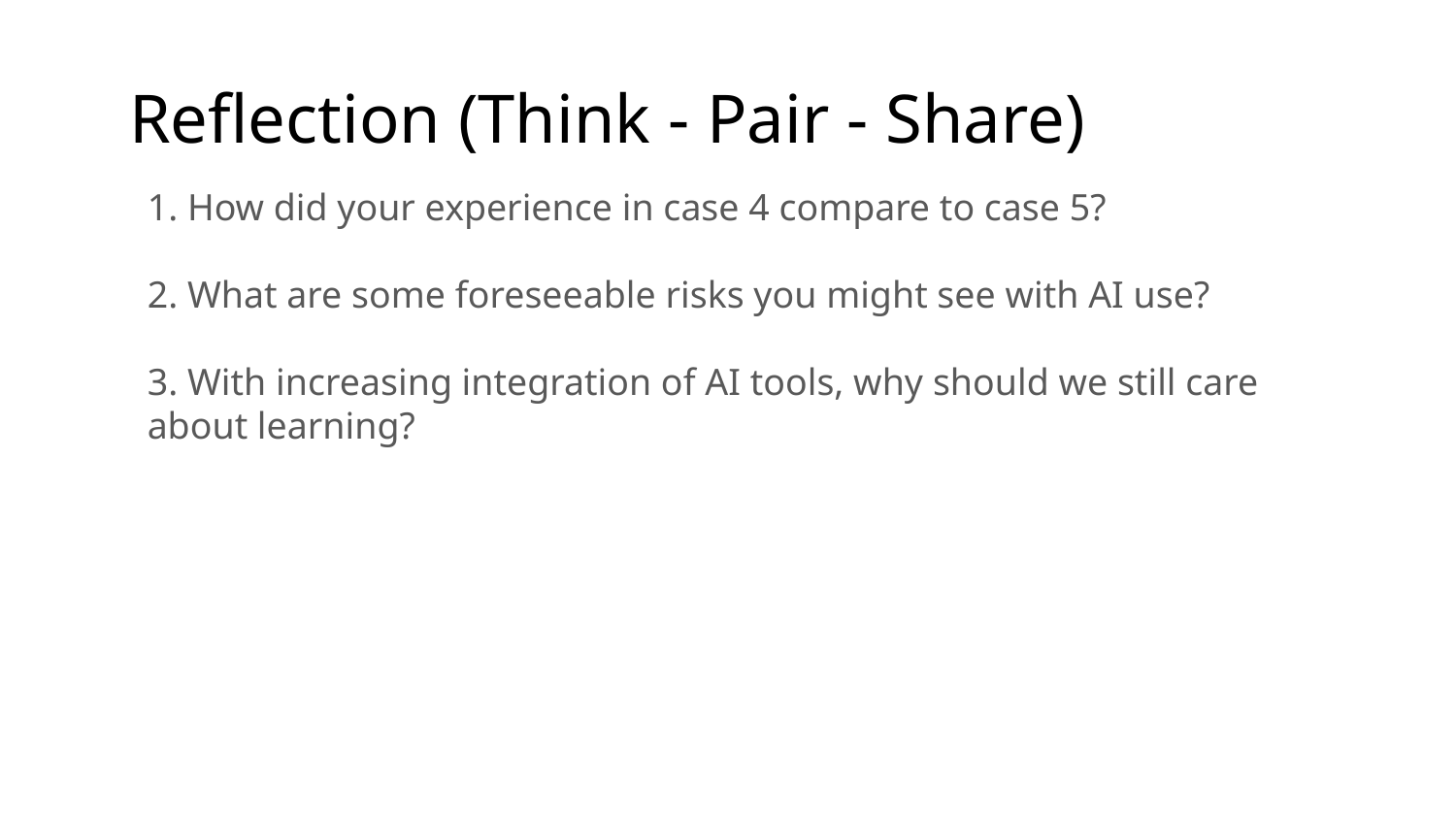

# Reflection (Think - Pair - Share)
1. How did your experience in case 4 compare to case 5?
2. What are some foreseeable risks you might see with AI use?
3. With increasing integration of AI tools, why should we still care about learning?

## Slide 31
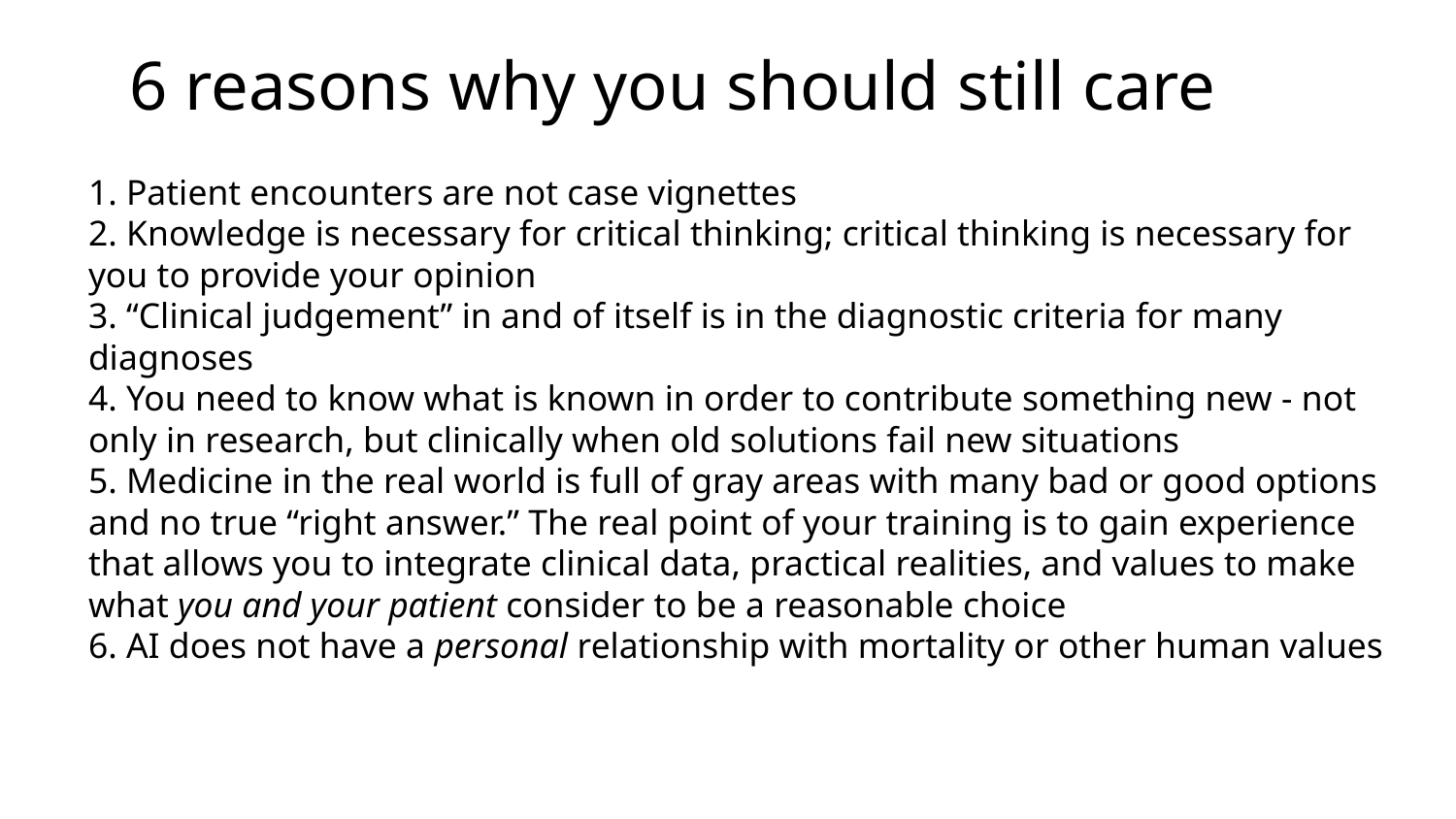

# 6 reasons why you should still care
1. Patient encounters are not case vignettes
2. Knowledge is necessary for critical thinking; critical thinking is necessary for you to provide your opinion
3. “Clinical judgement” in and of itself is in the diagnostic criteria for many diagnoses
4. You need to know what is known in order to contribute something new - not only in research, but clinically when old solutions fail new situations
5. Medicine in the real world is full of gray areas with many bad or good options and no true “right answer.” The real point of your training is to gain experience that allows you to integrate clinical data, practical realities, and values to make what you and your patient consider to be a reasonable choice
6. AI does not have a personal relationship with mortality or other human values

## Slide 32
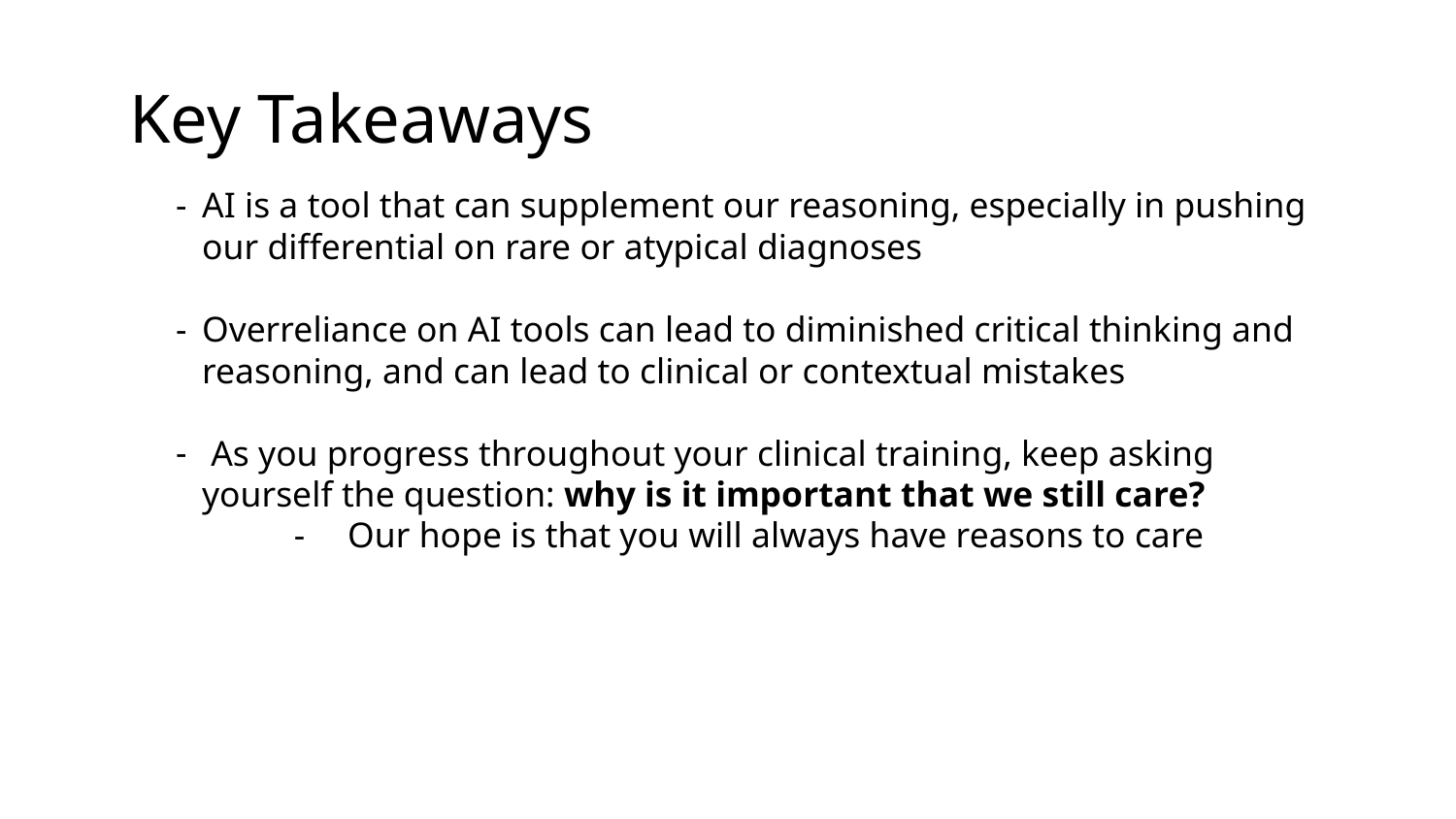

# Key Takeaways
AI is a tool that can supplement our reasoning, especially in pushing our differential on rare or atypical diagnoses
Overreliance on AI tools can lead to diminished critical thinking and reasoning, and can lead to clinical or contextual mistakes
 As you progress throughout your clinical training, keep asking yourself the question: why is it important that we still care?
Our hope is that you will always have reasons to care

## Slide 33
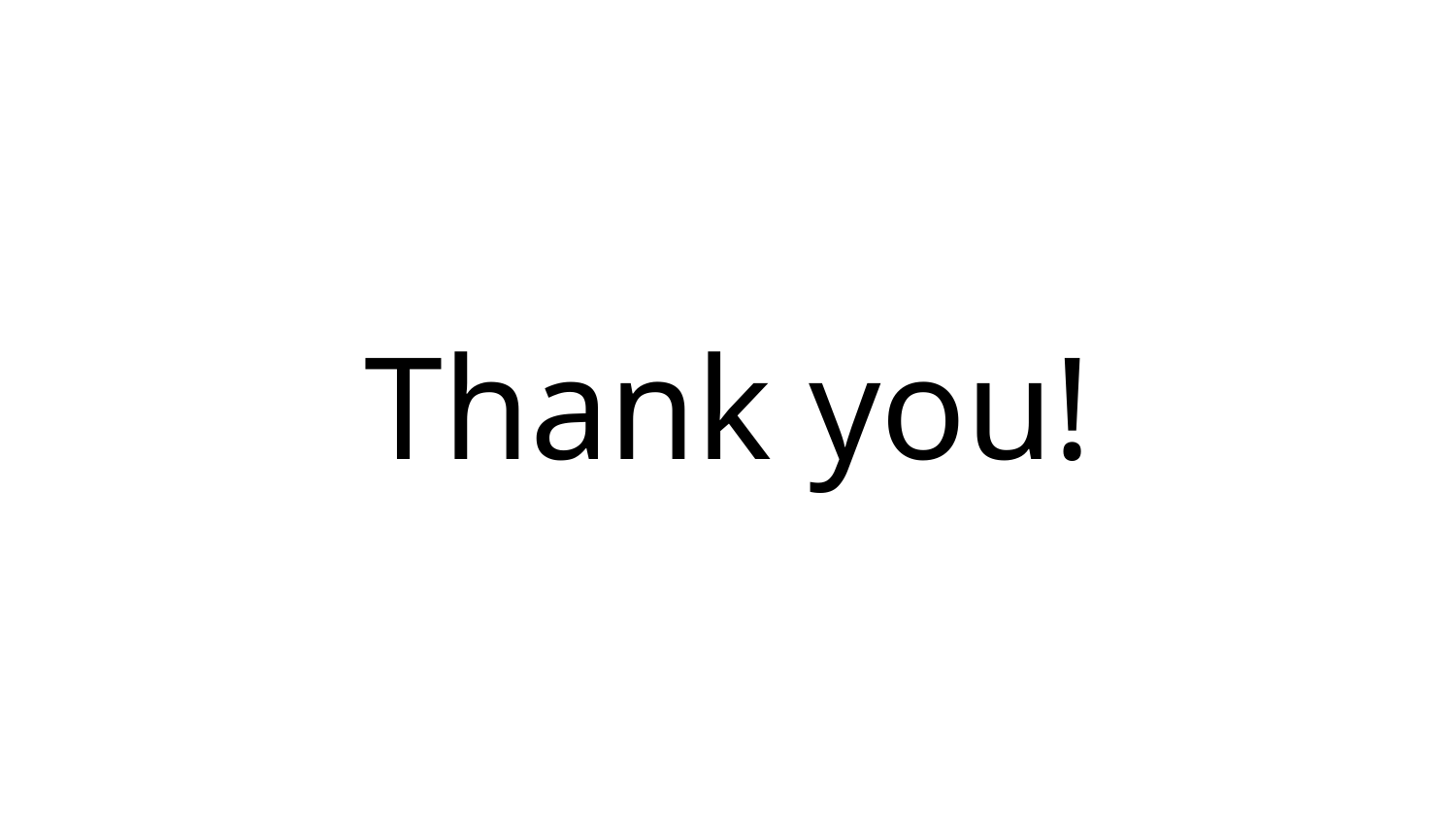

# Thank you!
