# Supplementary material for: Artificial Intelligence and Large Language Models: A Case-Based, Peer-Teaching Workshop for Preclinical Medical Students
Source: MedEdPORTAL. 2026 Jul 21;22:11621. doi: 10.15766/mep_2374-8265.11621 (PMC13385069; doi:10.15766/mep_2374-8265.11621)
Supplement: Supplementary file 1 — AI Didactic.pptxAI Workshop.pptxAI Workshop Presenter Guide.docxAI Workshop Case List.docxPre- and Postsurvey.docx [file mep_2374-8265.11621-s001.zip › C. AI Workshop Presenter Guide.docx]

**Interactive Case-Based Artificial Intelligence Workshop Notes:**

***INSTRUCTIONS:***

Please use the following notes as a presenter guide for facilitating your workshop session. Dialogue is simply suggested, so feel free to adjust as you see fit. Some suggested time labels (starting at time 0:00 representing hours:minutes) are provided for reference – please adjust timing to fit the flow of your room and total allotted time.

As an overview, this workshop consists of 3 parts. The first part involves using LLMs (large language models) for studying, a core activity for any student. This first part should take approximately 30 minutes and involves 2 exercises asking students to compare LLMs to traditional resources for studying and refine LLM prompts in the context of self-directed learning. The second part involves using LLMs to interact with documents and find clinical evidence. This second part should take approximately 30 minutes and involves an exercise to use an LLM to summarize a provided document and another to find clinical evidence using traditional means vs different LLMs. Finally, the third part involves using LLMs in the clinical context for diagnostic reasoning. This third part should take approximately 40 minutes and asks students to work through 2 clinical cases. In the first case, they must think through the case without AI then can use AI to add to their initial thoughts. In the second case, students are asked to directly turn to AI then see what they can add themselves. By comparing the experience in the two cases, students are exposed to the potential cognitive risks of immediately turning toward AI first. This transitions into a final reflection/discussion on potential risks of AI use for these students early on in their clinical training and thoughts on the value of knowledge in the age of these AI tools.

***Prior to Workshop***

- Please provide Appendix D (“AI Workshop Case List”) electronically to students so they can reference all cases in full during the workshop.

- Please also provide students with a review article electronically to be used for Case 2 (as described below/in Appendix B).

**Introduction (0:00):**

Welcome to the workshop component of our AI (artificial intelligence) session. *[Facilitators please introduce yourselves]* In this workshop, we hope to go through some use cases and exercises with AI that might be relevant for your medical education. In doing so, we aren’t saying that these LLMs (large language models) are the only tools you should use - rather one of many. And in this workshop, we hope that we can explore this tool with you all and have an open discussion of its strengths and limitations. This will be a very hands-on workshop in 3 parts- so we hope you join us on your laptops!

**Part 1: Studying**

*Introduction*: Medical students study a lot. We are well aware that there already exists many “traditional” sets of resources to pull from. The question is: are there ways we can use AI tools to supplement our studying?

*Aims:* The Aims for this part will be that [*read the 2 aims on slide*]. By the end of this first part, we hope that we can practice creating and modifying different LLM prompts for use in our studying and compare outputs to traditional resources!

*Case 1: A Question Appears* **(0:10-0:20 pairs work; 0:20-0:25 brief discussion)**: First exercise. Let’s say you are studying and you encounter the following question. *[Just read the first question; let students read the rest in their pairs]* You can read the rest on your own - but getting into our exercise …

[*Note: This case generated using AI tool GPT4.1 via prompt “please write a usmle exam question for step 1 that helps highlight high yield details for different hematologic malignancies” in order to demonstrate how AI tools can be used to create practice questions.*]

*Exercise 1* **(0:10-0:20 pairs work; 0:20-0:25 brief discussion):** When you see a question like this, hopefully you all know at this point that getting the answer is only a small part of the studying process. Learning and understanding the underlying concept is far more important. SO how would we go about learning about the topic tested in this question? Please go back and read the question. Then in pairs, one partner will try to learn more and collect information via a “traditional resource” (ie: your notes, uptodate, amboss, uworld) while the other partner will learn more using an LLM model of their choice. After around 5 minutes, please compare the information you collected. Were there differences in the information collected? Was one strategy more or less comprehensive? Accurate? Efficient? We will discuss.

*Exercise 2* **(0:20-0:30)**: Okay for our next exercise, if you haven’t already, we will take a few minutes to modify the last LLM prompt and see if you can improve the output. (If you only used traditional resources last time, try a few prompts of your own now!) We will discuss: Were there any phrases or prompts that provided a more desired output?

*[Note: If anyone asks, the answer to the sample case is B - Detection of BCR-ABL gene. This is describing CML as suggested by the low LAP, left shift w/ basophilia, 9;22 translocation. (A) involves B-cells characteristic for CLL, (C) involves Smudge cells classic for CLL. (D) Involves rouleaux formation on blood smear classic for multiple myeloma. (E) Involves TRAP-positive cells seen in hairy cell leukemia.]*

*Key Takeaways*: LLMs can be efficient ways to supplement traditional resources for studying!

Their perks are that they can dynamically

- Explain concepts. Meaning if you don’t understand a topic or Uworld explanation you can have a conversation asking more about it. Also practice tests may have poorly explained answer choice explanations, especially for choices which are incorrect. LLMs are very good at helping to add on and clarify these explanations.
- LLMs are also helpful in organizing information efficiently - so instead of digging through notes or if you want a nice compare/contrast table of high yield information - you can just generate it. This can be an efficient way to review and compare many topics at once. Additionally, unlike tables provided by other sources, you can modify AI generated tables to cater to specific diseases or information. (Let’s say you have a table on CML, CLL, Multiple Myeloma, Hairy Cell Leukemia - you can easily add ALL/AML as another column, take things out, or add information.
- Finally, LLMs can create practice questions, you can use phrases like please write a USMLE question to differentiate X and Y. The question you saw before was from a prompt in an LLM (GPT4.1)! And different ones can be made even with the same prompt.

**Part 2: Document Retrieval and Evidence (0:30)**

*Introduction:* Let’s now turn to our second part on document retrieval and evidence.

*Aims:* In this part our aims are [*read the aims*].

*Case 2: A Document Query and Analysis* **(0:35-0:42 exercise; 0:42-0:45 brief discussion)**: Now we will begin by uploading a document into an LLM and asking it to summarize this document. *(Please electronically provide students with any standard, accessible review article of any topic per discretion of instructor. An example review article on cirrhosis is provided below).* Please upload the document into an LLM of your choice and trial different prompts to summarize this document.

Example Review Article: Tapper EB, Parikh ND. Diagnosis and Management of Cirrhosis and Its Complications: A review. Jama. 2023; 329(18):1589-1602. doi:10.1001/jama.2023.5997

*Discussion* **(0:35-0:42 exercise; 0:42-0:45 brief discussion)**: We can discuss all these questions at once. Were there any surprises to using LLMs to try to summarize this document? Did anyone find certain prompts that worked or didn’t work? What are some strengths and limitations of using LLMs to summarize documents?

*Retrieval-Augmented Generation:* What you just did, conceptually involves making an LLM pull information from a provided document, certain LLMs use this concept more systematically and at large scale in a process called Retrieval-Augmented Generation (RAG). As described in your lecture, traditional large language models predict the next token based on prior context and pre-trained architecture. RAGs combine LLMs with systemic and explicit retrieval of external information from a search engine or database of documents for each query. This allows RAGs to provide generally more accurate information, from specific, cited sources or evidence, which can be updated regularly (past the time an LLM was trained). As shown, overall, a traditional LLM gets an input and generates an output. RAG models get an input, search and retrieve relevant information, and generate an output using this retrieved information. A common RAG model used in the medical field in the United States is OpenEvidence.

*Case 3: A Search for Evidence* **(0:45-0:55)**: Alright let’s move on to case 3. [*Read case + directions*]. You will have 10 minutes.

*Discussion* **(0:55-1:00):** For our discussion, we wanted to talk about [*read discussion questions*].

*Key Takeaways:*

- LLMs can be used to summarize information from provided documents though prompt specificity can impact the usefulness and clarity of these summaries.
- RAG models utilize retrieved information from updated or collated databases to generate output.
- AI tools can be valuable tools to efficiently find clinical evidence though the model used can affect the evidence which is found.
- We wanted to highlight.
  - These skills can be especially useful on the wards when you’re asked to put together a case conference or presentation, find evidence, research, give a chalk talk, etc.
  - However, remember to always critically appraise AI-generated summaries—AI can streamline evidence review, but cannot replace clinical judgment or deep reading.

**Part 3: Clinical Reasoning and the Value of Knowledge in the Present and Future (1:00)**

*Introduction:* We will now enter our third part on clinical reasoning and the value of knowledge in the present and future.

*Aims:* In this part our aims are [*read the aims*].

*Case 4: A Clinical Scenario Part 1* **(1:00-1:10)**: Please refer to your case worksheet for the following case. Spend 5-10 minutes creating your own differential diagnosis and next steps for work-up and management without the use of AI. You can discuss with your tablemates. Please note for the physical exam, we purposefully included some more commonly used abbreviations. A key is provided in the case list.

*Case 4: A Clinical Scenario Part 2* **(1:10-1:15)**: Now use your favorite AI model to expand your initial differential diagnosis and next steps. See what the AI might add.

*Discussion:* [*Ask each of these questions one by one. Try to limit the discussion to 5 minutes or less to allow ample time for the next exercise and discussion. Note: The goal of these cases is not the clinical information or clinical decision making but the process of using these AI tools. Please feel free to defer questions about the clinical case to focus on the exercises themselves if short on time.*]

*Case 5: A Second Scenario Part 1* **(1:20-1:25)**: For this case, we will give you 5 minutes, because we want you to first directly consult an LLM. You can copy and paste this in, summarize things, whatever you like.

*Case 5: A Second Scenario Part 2* **(1:25-1:30)**: Now stop using AI. And independently think through how you might expand the AI provided differential and suggested steps in management.

*Case 5 Conclusion:* [*Provide a brief conclusion to the case. Note again – the point of all these cases is not to dive into the actual clinical content/problem presented and we would suggest deferring most case related questions. It is the process of doing these cases which is most important*].

*Reflection* (**1:30-1:35**): Let’s take a moment to individually reflect on that process integrating AI tools in these 2 different ways. How did your experience in case 4 compare to case 5? Was it difficult to think of your own differential after AI gave you one? Or expand on what was given? And also think - with the increasing integration of AI tools, what are some foreseeable risks you might see. And Why should we still care about learning? After thinking, share with a partner!

As you all enter your clinical spaces, your learning is in your hands. I’m sure it can be very tempting, especially in the beginning, to turn to AI. It will be hard in the beginning to come up with differentials, management, or that clinical reasoning. But it really gets easier the more you do it. And you really need to train yourself to do it often in order to build this skill up and not just rely on quick answer.

*6 reasons why you should still care* (**1:35-1:40**): We wanted to share some of our personal thoughts on why we should still care in the age of AI. [*Read through, adjust, or expand upon reasons listed in slide as you see fit*].

*Key Takeaways:* Alright so just to wrap up with some final key takeaways of our own: AI is clearly a powerful tool that can supplement our reasoning and provide quick answers. However, hopefully, as you may have seen with the second part of the last case, turning immediately to AI tools might make it hard to think on our own afterwards. It can be pretty difficult not to anchor on what AI is saying to create our own differentials or ideas. Over relying on AIs tools, can severely diminish our own abilities to critically think, reason, and develop these necessary skills. In the end, AI tools are probably best at expanding our differential or thinking in complex cases after we have given some thought to things. They also can be good in categories that do not naturally play to the strengths of a human mind like exceedingly rare/niche diagnoses. As you progress throughout your clinical training, keep asking yourself the question: why is it important that we still care? Our hope is that you will always have reasons to care

So that’s our workshop. Thank you all for your engagement and participation.
