# Supplementary material for: Artificial Intelligence and Large Language Models: A Case-Based, Peer-Teaching Workshop for Preclinical Medical Students
Source: MedEdPORTAL. 2026 Jul 21;22:11621. doi: 10.15766/mep_2374-8265.11621 (PMC13385069; doi:10.15766/mep_2374-8265.11621)
Supplement: Supplementary file 1 — AI Didactic.pptxAI Workshop.pptxAI Workshop Presenter Guide.docxAI Workshop Case List.docxPre- and Postsurvey.docx [file mep_2374-8265.11621-s001.zip › D. AI Workshop Case List.docx]

**Interactive Case-Based Artificial Intelligence Workshop**

**Case List**

***INSTRUCTIONS***

This case list is provided for reference and will be used during workshop exercises.

**Case 1: A Question Appears***

Question: A 56-year-old man presents with progressive fatigue, night sweats, and unintentional weight loss. Physical examination reveals massive splenomegaly. Laboratory studies show:

Hemoglobin: 10.2 g/dL

White blood cell count: 90,000/mm³ (with increased mature granulocytes and myelocytes)

Platelets: 600,000/mm³

Peripheral blood smear: marked left shift with increased basophils

Leukocyte alkaline phosphatase (LAP): decreased

Which of the following findings is most likely to differentiate this patient’s diagnosis from other chronic hematologic malignancies?

1. Presence of CD5+ neoplastic B-cells
2. Detection of BCR-ABL fusion gene
3. Smudge cells on peripheral smear
4. Rouleaux formation on blood smear
5. Tartrate-resistant acid phosphatase (TRAP)-positive cells

**This case generated using AI tool GPT4.1 via prompt “please write a usmle exam question for step 1 that helps highlight high yield details for different hematologic malignancies” in order to demonstrate how AI tools can be used to create practice questions.*

**Case 2: A Document Retrieval**

Please use the review article provided electronically by instructor. If none are provided, the following cirrhosis review article is an example of one that can be used: Tapper EB, Parikh ND. Diagnosis and Management of Cirrhosis and Its Complications: A Review. Jama. 2023; 329(18):1589-1602. doi:10.1001/jama.2023.5997

**Case 3: A Search for Evidence**

On a medicine clerkship rotation, a patient with cirrhosis comes in presenting with acute variceal bleeding. Your attending asks you to find and evaluate the evidence regarding when an early TIPs procedure (transjugular intrahepatic portosystemic shunt placement within 72 hours of admission) is indicated.

**Case 4: A Clinical Scenario**

- **HPI:**
  - 79M h/o COPD, A fib (on eliquis), HTN, HLD, aortic valve replacement (c/b post-op aortic aneurysm now s/p resection of portion of aorta and replacement of graft) who presents with SOB and hypoxia.
  - Patient reports that he had COVID approximately 6 weeks ago. Approximately 5 weeks ago he was not feeling well so went to urgent care where he had a chest x-ray and was diagnosed with pneumonia. He completed a 5-day course of antibiotic as well as 5 days of prednisone at that time. He had ongoing symptoms so subsequently completed another 5-day course of prednisone. More recently he completed a 10-day course of prednisone which ended approximately 4 days ago due to ongoing respiratory symptoms. He states that 3 to 4 days ago he was feeling more short of breath. He has oxygen at home which he only uses planes and when he goes to Denver. He states that 3 to 4 days ago he started using the oxygen, initially 2 L, uptitrated to 4 L which he used all day yesterday. This morning he was feeling more short of breath so he called 911. On EMS arrival, his SpO2 was 80s on nasal cannula. He had a non-sustained 5-beat run of VT with EMS; resolved w/ O2. Pt is on eliquis; no missed doses.
- **Physical Exam**
  - Vitals – BP 130/85| Pulse 109 | Temp 98.8°F | RR 20 | SpO2 96% on 4L NC
  - General –  NAD. Awake and Alert.  Ill-appearing.
  - HEENT – Normocephalic and atraumatic. EOMI.
  - Cardiac – RRR, no m/r/g. No JVD.
  - Pulmonary – No iWOB on 4L NC, mild rales, otherwise CTAB.
  - Abdomen – NTND. No guarding, no rebound.
  - Extremities – No peripheral edema
  - Neurologic – No FND.
  - Mental Status – AAOX3.

KEY:

- - - NAD = no acute distress
    - EOMI = extraocular movements intact
    - RRR = regular, rate and rhythm
    - m/r/g = murmur/rub/gallops
    - JVD = jugular venous distention
    - iWOB = increased work of breathing
    - NC = nasal canula
    - CTAB = clear to auscultation bilaterally
    - NTND = nontender, nondistended
    - FND = focal neurological deficits
    - AAO x3 = alert, awake, oriented (to person, place, time)
- **Lab Values * Key values bolded, reference ranges approximate**
  - **Sodium: 127 (135-145 mmol/L)**
  - Potassium: 4.8
  - Chloride: 92
  - CO2: 26
  - Anion Gap: 9
  - BUN: 11
  - Creatinine: 0.70
  - Estimated GFR(CKD-EPI): 94
  - Glucose: 112
  - Calcium: 8.4
  - **hs-Troponin T: 38 -> 30 (<14 ng/L)**
  - **NT-ProBNP: 1,477 (<450 pg/mL)**
  - Coronavirus SARS-CoV-2: Negative
  - Rapid Influenza A, Molecular: Negative
  - Rapid Influenza B, Molecular: Negative
  - RSV by PCR: Negative
  - **C-Reactive Protein (CRP): 156.0 (0.00-10.0 mg/L)**
  - **WBC: 8.92 (4.00-10.00 k/uL)**
  - RBC: 4.33
  - Hemoglobin: 13.0
  - Hematocrit: 37.8
  - MCV: 87
  - MCH: 30.0
  - MCHC: 34.4
  - RDW: 14.7
  - RDW-SD: 47.6
  - Platelet Count: 276
  - MPV: 8.9
  - Nucleated RBC: 0
  - Neutrophil: 80.0
  - Immature Granulocyte (Meta, Myelo, Promyelocyte): 0.8
  - Lymphocyte: 5.9
  - Monocyte: 12.0
  - Eosinophil: 0.9
  - Basophil: 0.4
  - **Absolute Neutrophil Count: 7.13 (1.50-8.0 k/uL)**
  - Absolute Immature Granulocyte (Meta, Myelo, Promyelocyte): 0.07
  - Absolute Lymphocyte Count: 0.53
  - Absolute Monocyte Count: 1.07
  - Absolute Eosinophil Count: 0.08
  - Absolute Basophil Count: 0.04
- **Imaging**
  - CT Angiogram Chest PE (Edited Result - FINAL)
    - Impression:

1. No pulmonary embolism.

2. Moderate cardiomegaly with interstitial pulmonary edema indicated through asymmetric right lung predominant patchy ground-glass opacities and trace bilateral pleural effusions.

3. Severe panlobular lower lobe predominant emphysema with layering of fluid in a few left lower lobe large cysts/bullae. Correlate for any clinical evidence of infection.

4. Patchy heterogeneous opacity of the right lung base may reflect an infectious/inflammatory process.

5. Mediastinal lymphadenopathy, measuring up to 1.5 cm in the lower paratracheal region. This is enlarged compared to the prior examination. While this may be reactive, lymphoproliferative disorders and malignancy are within the differential and follow-up chest CT in 3 months must be performed.

**Case 5: A Second Scenario**

- **HPI**
  - An 80-year-old female with history of anxiety and CKD presents to the ED as a referral from urgent care with worsening shortness of breath, cough, and fevers, for which she was found to be hypoxemic.
  - She reports several weeks of worsening productive cough, intermittent fevers, and several days of shortness of breath. She went to urgent care one week ago for which chest xray was negative, but she was given a 5-day course of doxycycline. She thinks it may have briefly helped, but took only 3 days of it due to nausea.
  - She also reports 6 weeks of diarrhea, nausea, fatigue, and weight loss. She has lost about 30 pounds in this time frame that she attributes to loss of appetite from nausea.
  - One month prior to presentation, she had cutaneous shingles. She was given valacyclovir but stopped after two days due to nausea.
- **Past Medical History:**
  - Anxiety
  - CKD-3
  - Seasonal allergies
- **Medications**:
  - Loratadine
  - Flonase nasal spray
- **Allergies**:
  - None
- **Social History:**
  - Never smoker, no drugs
  - 2 alcoholic beverages per week
  - Divorced, currently lives with two housemates in Boston
  - No pets, birds
  - No camping/hiking
  - Was a writer and filmmaker
  - No recent travel, but ~10 years ago in Kenya making a documentary
- **Physical Exam**
  - BP 141/79 | Pulse 89 | Temp 39.1 °C (102.4 °F) | RR 18 | SpO2 94% on 4L NC
  - General: Sleepy, thin
  - Cardiac: Regular Rate and Rhythm, No JVD
  - Pulmonary: Clear anteriorly
  - Abdomen: Soft, non-tender, non-distended
  - Extremities: No edema Neuro: alert, oriented to person, place, and time, but slightly confused
  - Skin: no rashes
- **Laboratory Values**
  - WBC 10.58 (86% neutrophils, 5.8% lymphocytes)
  - Hemoglobin 12.1
  - Platelets 296
  - Cr 1.02
  - BUN 19
  - Sodium 133
  - HCO3 22
  - Potassium 3.6
  - ProBNP 400
  - Extended RVP negative
  - Blood cultures pending
- **Imaging**
  - **CXR:**
    - Diffuse ground glass paucity with some mild septal thickening
  - **CT Chest:**
    - Diffuse ground glass opacities throughout both lungs with upper lung predominance with superimposed interlobular septal thickening. Additional more consolidative opacities in both lower lungs.
