# Supplementary material for: Artificial Intelligence and Large Language Models: A Case-Based, Peer-Teaching Workshop for Preclinical Medical Students
Source: MedEdPORTAL. 2026 Jul 21;22:11621. doi: 10.15766/mep_2374-8265.11621 (PMC13385069; doi:10.15766/mep_2374-8265.11621)
Supplement: Supplementary file 1 — AI Didactic.pptxAI Workshop.pptxAI Workshop Presenter Guide.docxAI Workshop Case List.docxPre- and Postsurvey.docx [file mep_2374-8265.11621-s001.zip › E. Pre- and Postsurvey.docx]

***INSTRUCTIONS****:*

Items used in pre- and post-session evaluations. Items under the “pre-only” section were only in the pre-session evaluation, and items under the “post-only” section were only in sthe post-session evaluation. Items under “Both” were in both evaluations. Minor wording changes made compared to original evaluation to make this appendix more generalizable.

___________________________________________________________________________

- **Pre-Only (2):**
  - How often do you use AI (artificial intelligence) tools to study?
    - 1 = Never, 2 = Rarely, 3 = Sometimes, 4 = Often, 5 = Always
  - How often do you use AI tools for clinical activities?
    - 1 = Never, 2 = Rarely, 3 = Sometimes, 4 = Often, 5 = Always
- **Both (12)**:
  - I am a
    - Medical student; Dental student
  - I can describe at least two different LLMs (large language models) and their differences in their functions.
    - 1 = Strongly Disagree 2 = Disagree 3 = Neutral 4 = Agree 5 = Strongly Agree
  - I choose a specific LLM to fit my needs.
    - 1 = Strongly Disagree 2 = Disagree 3 = Neutral 4 = Agree 5 = Strongly Agree
  - I feel comfortable generating effective prompts for LLMs to supplement my studying.
    - 1 = Strongly Disagree 2 = Disagree 3 = Neutral 4 = Agree 5 = Strongly Agree
  - I intend to use LLMs to supplement my studying for standardized exams.
    - 1 = Strongly Disagree 2 = Disagree 3 = Neutral 4 = Agree 5 = Strongly Agree
  - I can explain how Retrieval-Augmented Generation (RAG) differs from traditional LLM output.
    - 1 = Strongly Disagree 2 = Disagree 3 = Neutral 4 = Agree 5 = Strongly Agree
  - I feel comfortable using LLMs to summarize provided documents.
    - 1 = Strongly Disagree 2 = Disagree 3 = Neutral 4 = Agree 5 = Strongly Agree
  - I feel comfortable using LLMs in place of traditional resources (ie: UpToDate, PubMed, Google, etc.) to find clinical evidence.
    - 1 = Strongly Disagree 2 = Disagree 3 = Neutral 4 = Agree 5 = Strongly Agree
  - I intend to use AI tools during future clinical experiences to help with creating a differential.
    - 1 = Strongly Disagree 2 = Disagree 3 = Neutral 4 = Agree 5 = Strongly Agree
  - I intend to use AI tools during future clinical experiences to provide additional confidence in my clinical management decisions.
    - 1 = Strongly Disagree 2 = Disagree 3 = Neutral 4 = Agree 5 = Strongly Agree
  - I believe that AI tools will affect the development of my clinical reasoning in my next phases of training.
    - 1 = Very negatively 2 = Negatively 3 = Neutral 4 = Positively 5 = Very positively
  - I believe LLMs and AI tools have a net positive effect for my education.
    - 1 = Strongly Disagree 2 = Disagree 3 = Neutral 4 = Agree 5 = Strongly Agree
- **Post-Only (4):**
  - My workshop was taught by:
    - Student Leaders
    - Faculty
  - I felt comfortable expressing my views in this workshop.
    - 1 = Strongly Disagree 2 = Disagree 3 = Neutral 4 = Agree 5 = Strongly Agree
  - I was satisfied by the didactic portion of this AI Session.
    - 1 = Strongly Disagree 2 = Disagree 3 = Neutral 4 = Agree 5 = Strongly Agree
  - I was satisfied by the workshop portion of this AI Session.
    - 1 = Strongly Disagree 2 = Disagree 3 = Neutral 4 = Agree 5 = Strongly Agree

Abbreviations: LLM = Large Language Model, AI = Artificial Intelligence
