# Supplementary material for: Artificial Intelligence and Large Language Models: A Case-Based, Peer-Teaching Workshop for Preclinical Medical Students
Source: MedEdPORTAL. 2026 Jul 21;22:11621. doi: 10.15766/mep_2374-8265.11621 (PMC13385069; doi:10.15766/mep_2374-8265.11621)
Supplement: Supplementary file 1 — AI Didactic.pptxAI Workshop.pptxAI Workshop Presenter Guide.docxAI Workshop Case List.docxPre- and Postsurvey.docx [file mep_2374-8265.11621-s001.zip › A. AI Didactic.pptx]

## Slide 1
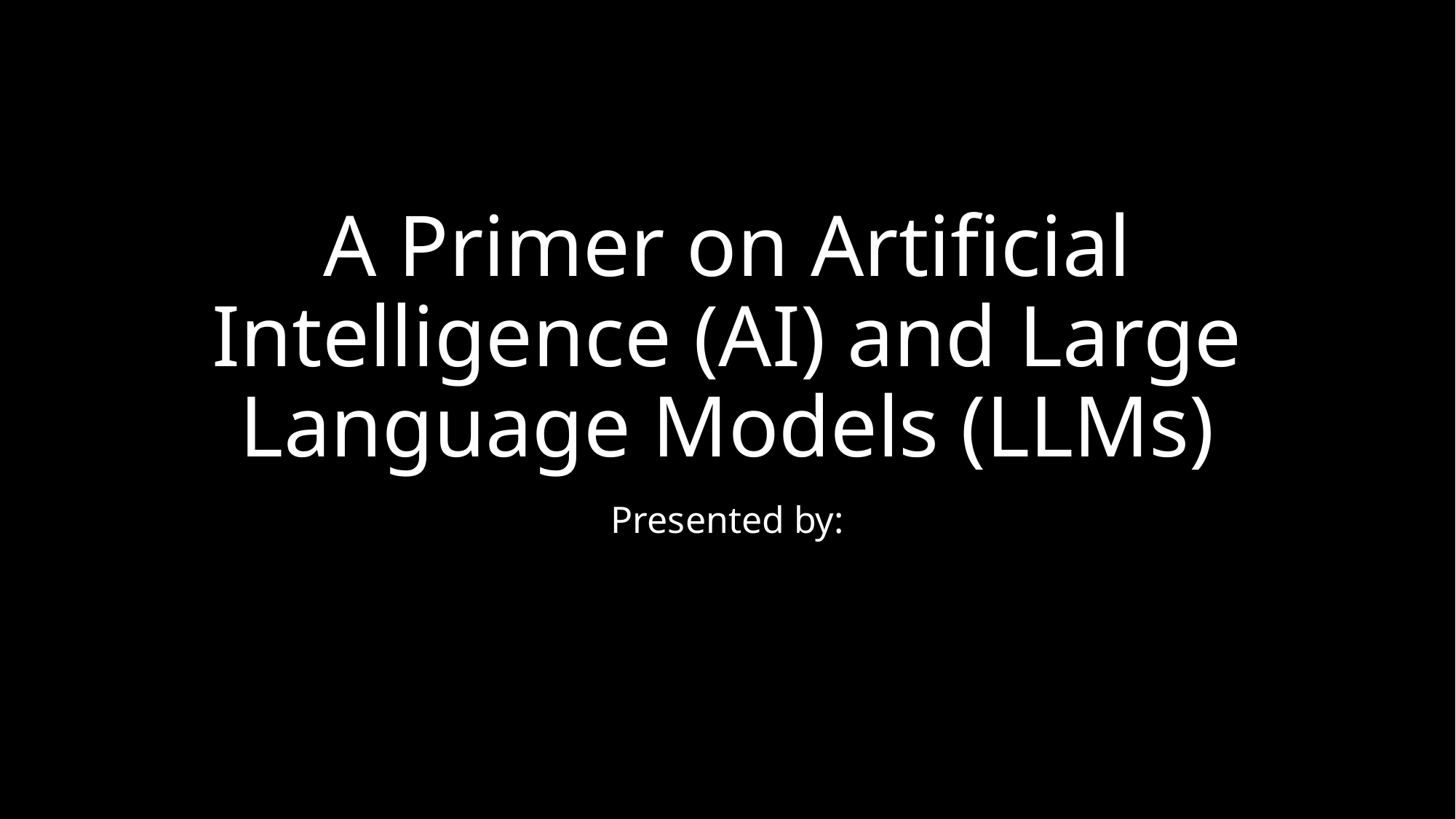

# A Primer on Artificial Intelligence (AI) and Large Language Models (LLMs)
Presented by:

## Slide 2
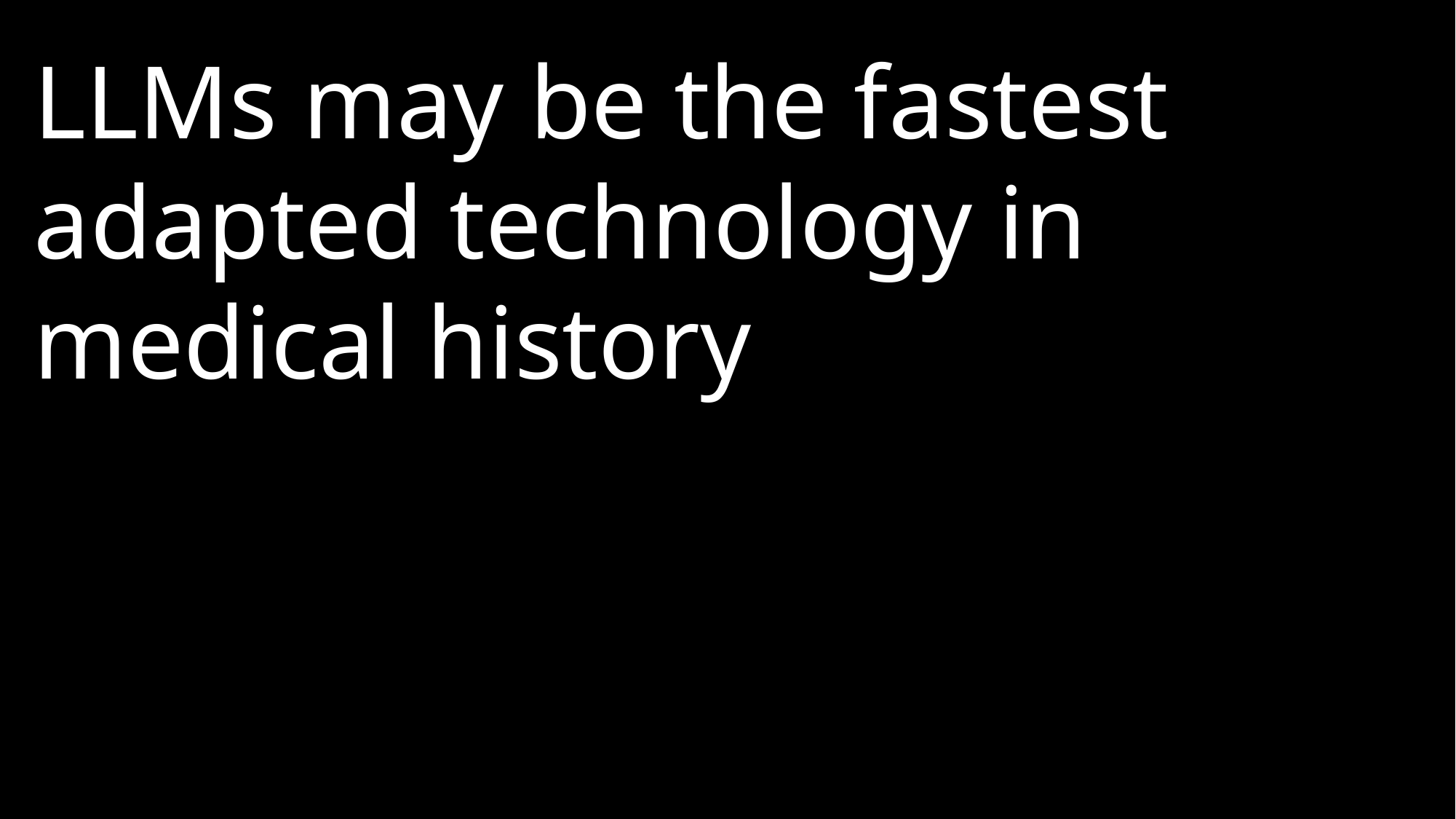

LLMs may be the fastest adapted technology in medical history

## Slide 3
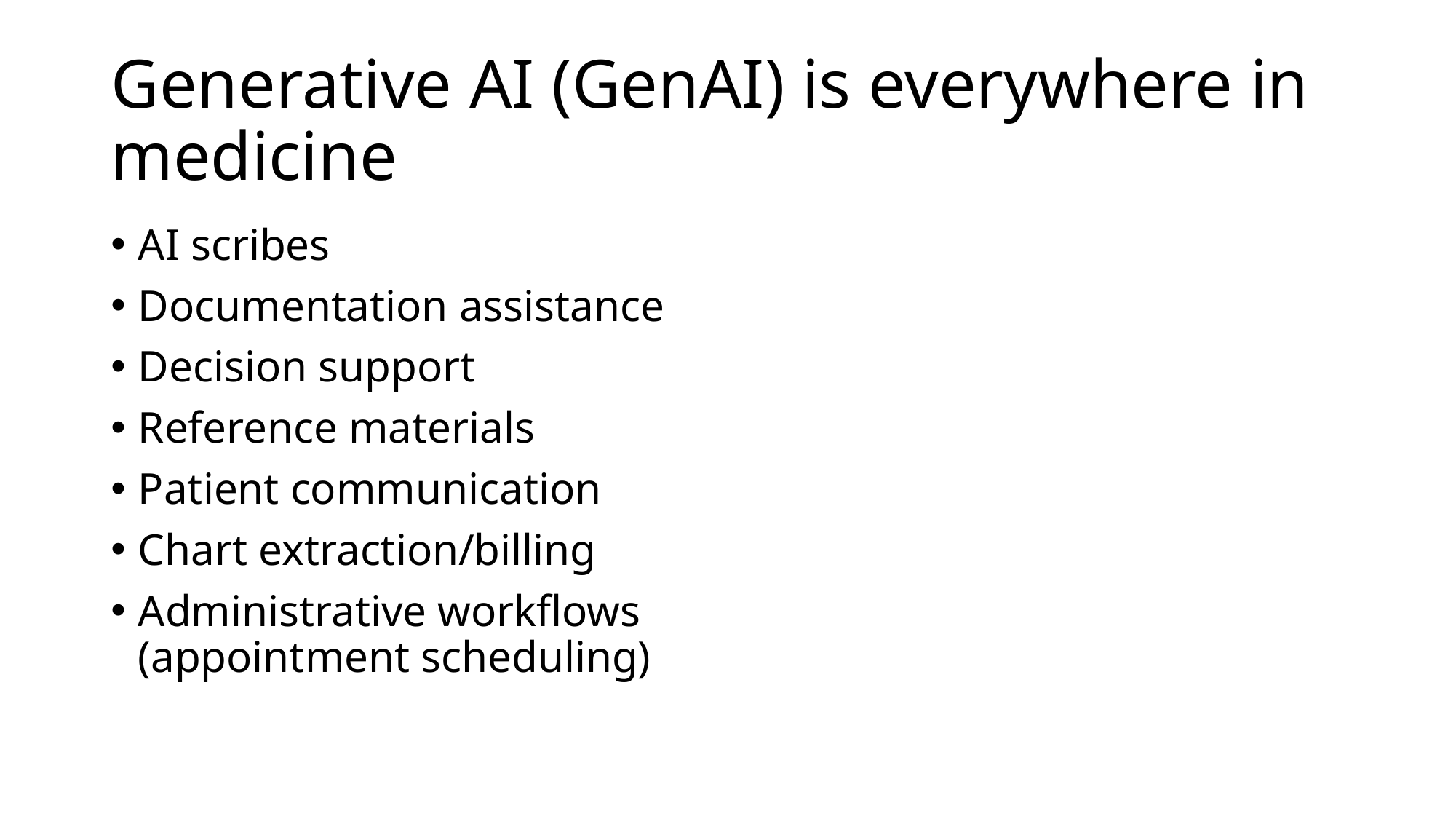

# Generative AI (GenAI) is everywhere in medicine
AI scribes
Documentation assistance
Decision support
Reference materials
Patient communication
Chart extraction/billing
Administrative workflows (appointment scheduling)

## Slide 4
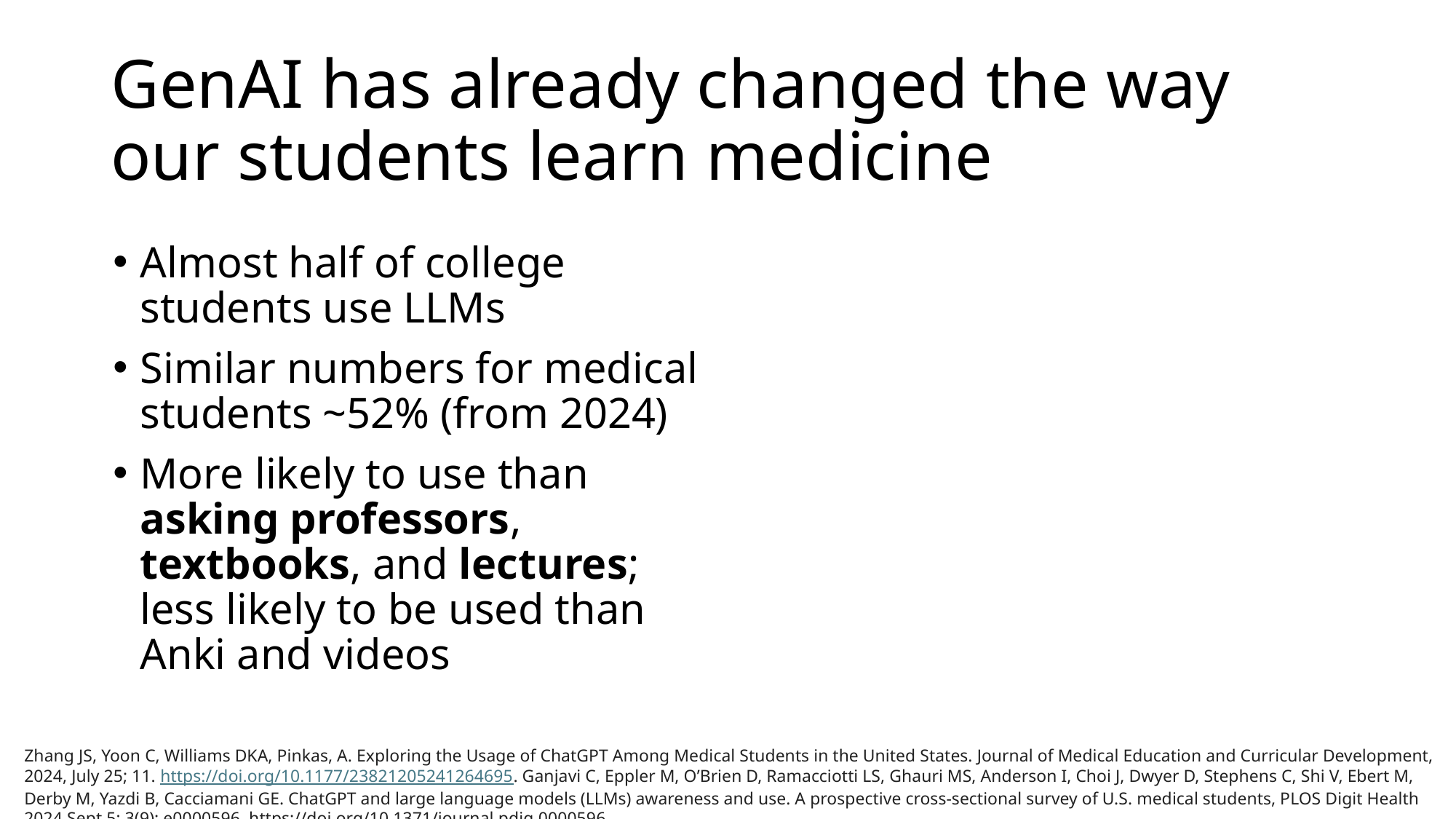

# GenAI has already changed the way our students learn medicine
Almost half of college students use LLMs
Similar numbers for medical students ~52% (from 2024)
More likely to use than asking professors, textbooks, and lectures; less likely to be used than Anki and videos
Zhang JS, Yoon C, Williams DKA, Pinkas, A. Exploring the Usage of ChatGPT Among Medical Students in the United States. Journal of Medical Education and Curricular Development, 2024, July 25; 11. https://doi.org/10.1177/23821205241264695. Ganjavi C, Eppler M, O’Brien D, Ramacciotti LS, Ghauri MS, Anderson I, Choi J, Dwyer D, Stephens C, Shi V, Ebert M, Derby M, Yazdi B, Cacciamani GE. ChatGPT and large language models (LLMs) awareness and use. A prospective cross-sectional survey of U.S. medical students, PLOS Digit Health 2024 Sept 5; 3(9): e0000596. https://doi.org/10.1371/journal.pdig.0000596

## Slide 5
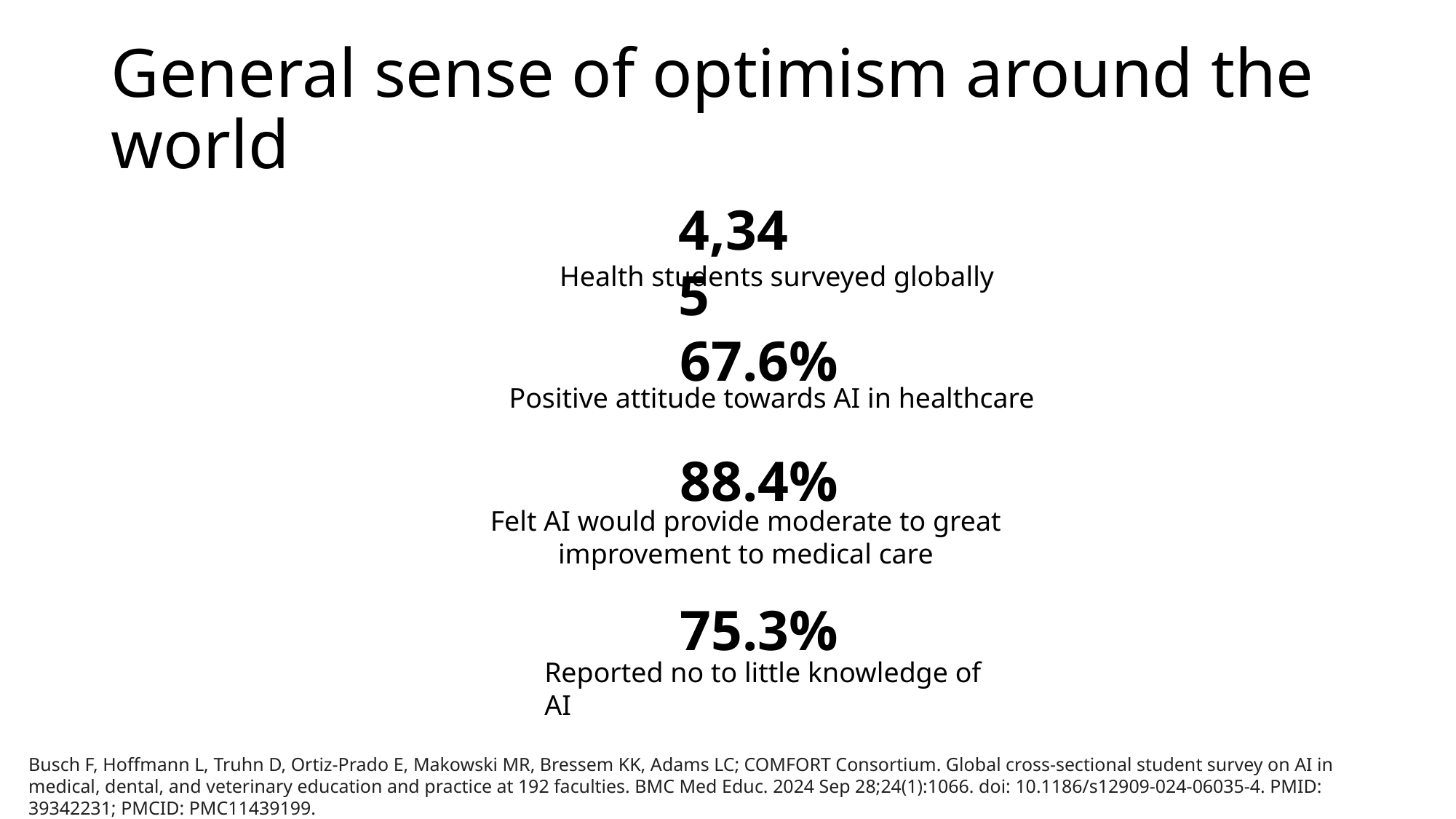

# General sense of optimism around the world
4,345
Health students surveyed globally
67.6%
Positive attitude towards AI in healthcare
88.4%
Felt AI would provide moderate to great improvement to medical care
75.3%
Reported no to little knowledge of AI
Busch F, Hoffmann L, Truhn D, Ortiz-Prado E, Makowski MR, Bressem KK, Adams LC; COMFORT Consortium. Global cross-sectional student survey on AI in medical, dental, and veterinary education and practice at 192 faculties. BMC Med Educ. 2024 Sep 28;24(1):1066. doi: 10.1186/s12909-024-06035-4. PMID: 39342231; PMCID: PMC11439199.

## Slide 6
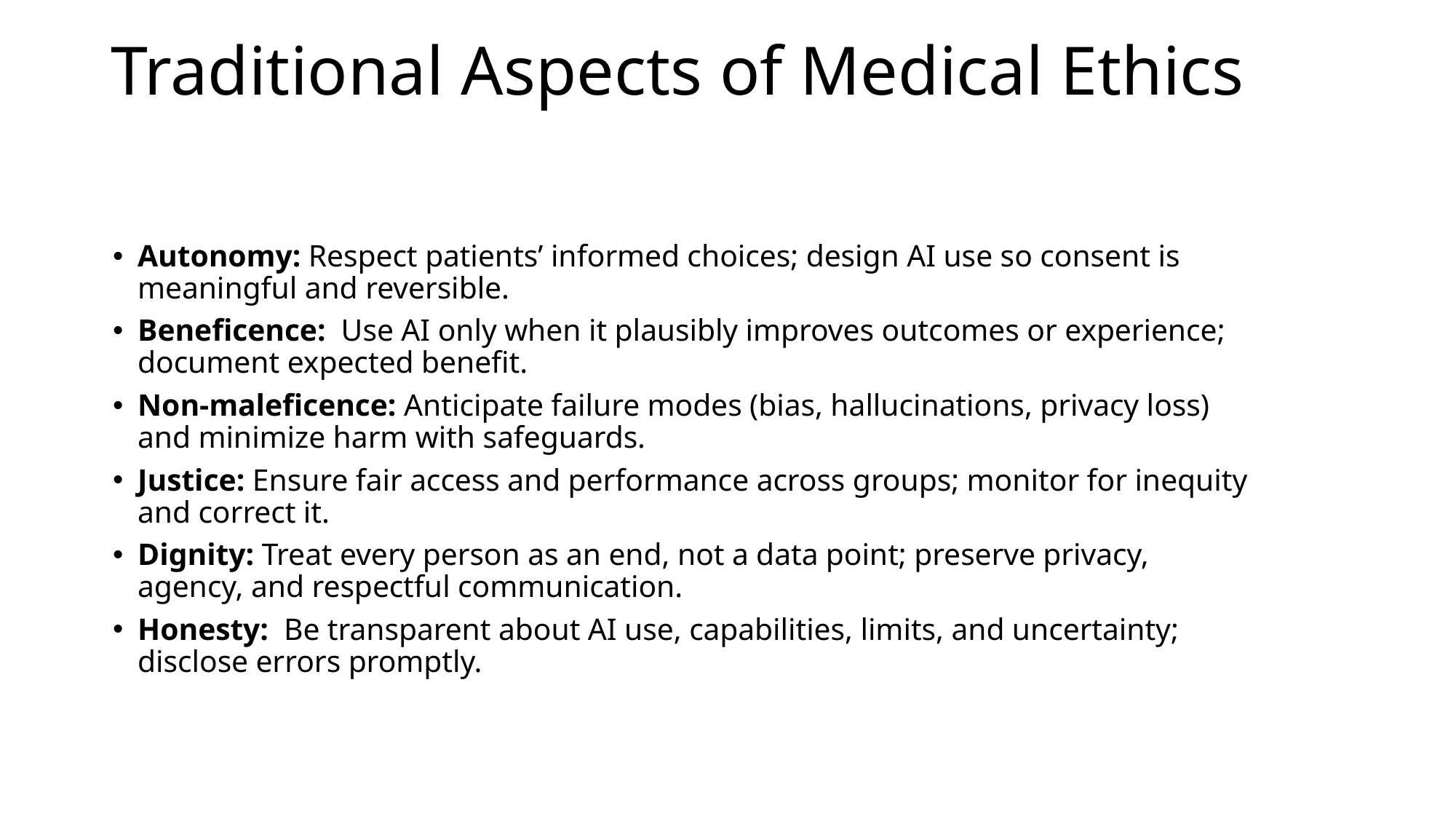

Traditional Aspects of Medical Ethics
Autonomy: Respect patients’ informed choices; design AI use so consent is meaningful and reversible.
Beneficence: Use AI only when it plausibly improves outcomes or experience; document expected benefit.
Non-maleficence: Anticipate failure modes (bias, hallucinations, privacy loss) and minimize harm with safeguards.
Justice: Ensure fair access and performance across groups; monitor for inequity and correct it.
Dignity: Treat every person as an end, not a data point; preserve privacy, agency, and respectful communication.
Honesty: Be transparent about AI use, capabilities, limits, and uncertainty; disclose errors promptly.

## Slide 7
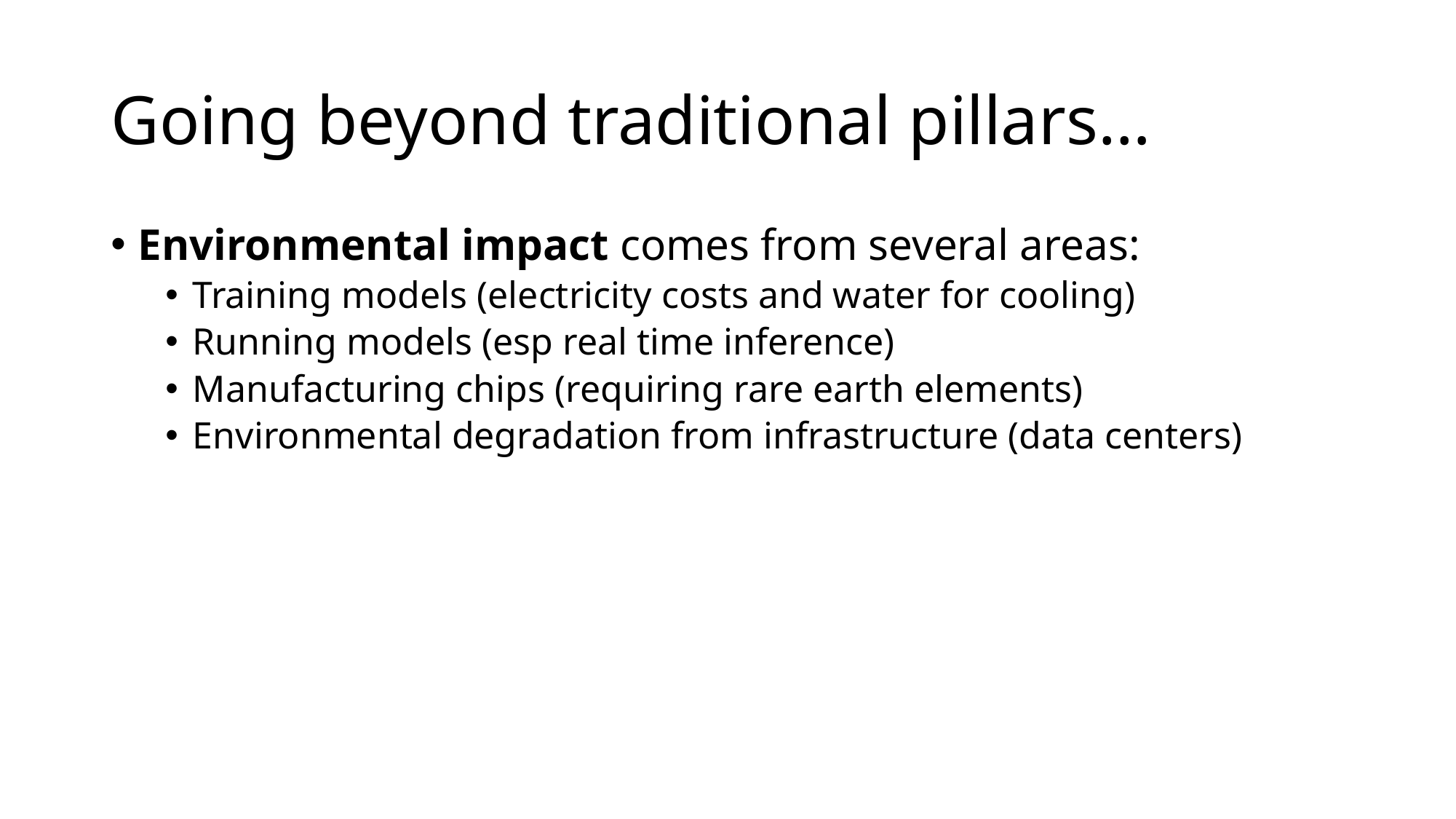

# Going beyond traditional pillars…
Environmental impact comes from several areas:
Training models (electricity costs and water for cooling)
Running models (esp real time inference)
Manufacturing chips (requiring rare earth elements)
Environmental degradation from infrastructure (data centers)

## Slide 8
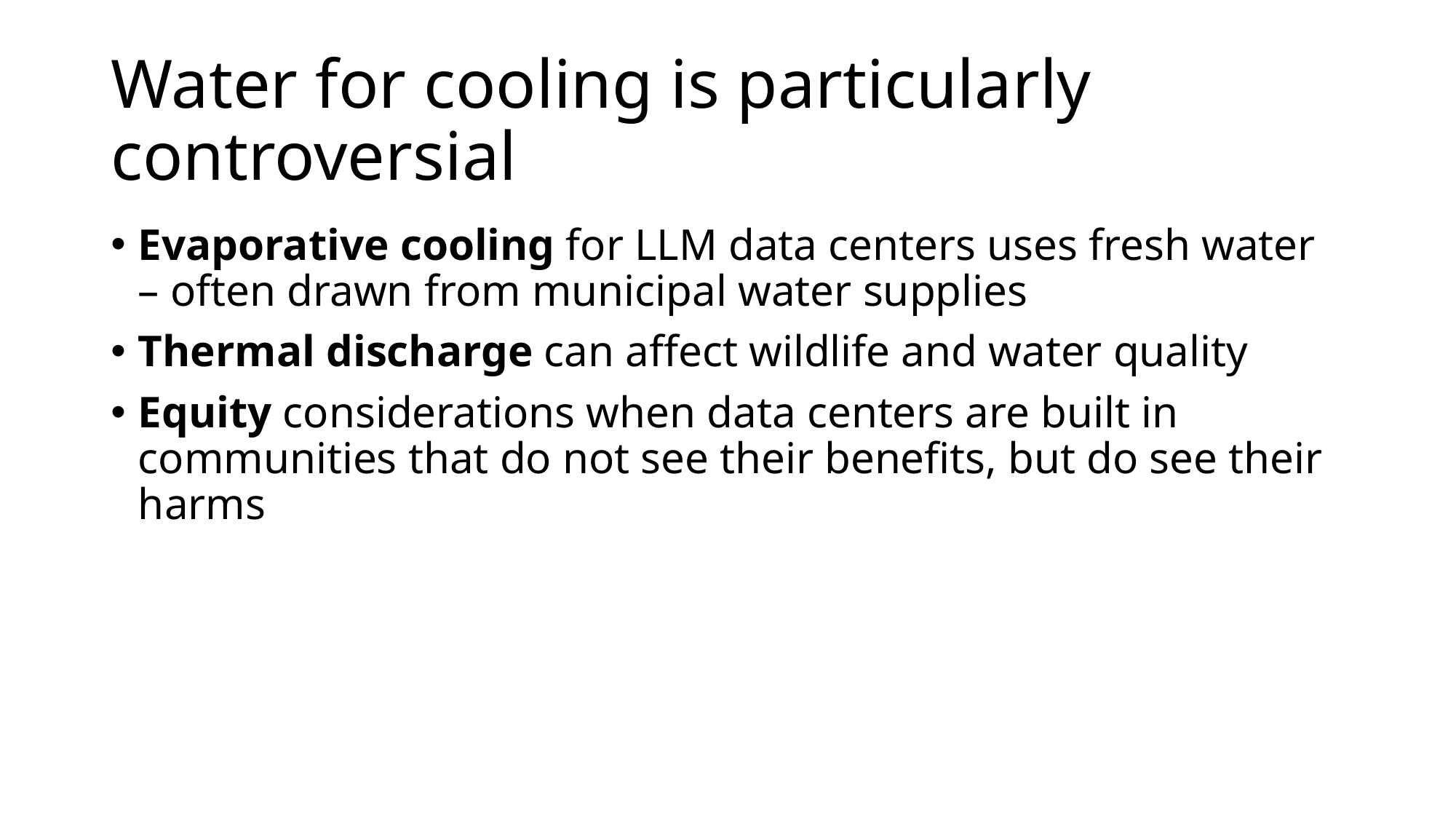

# Water for cooling is particularly controversial
Evaporative cooling for LLM data centers uses fresh water – often drawn from municipal water supplies
Thermal discharge can affect wildlife and water quality
Equity considerations when data centers are built in communities that do not see their benefits, but do see their harms

## Slide 9
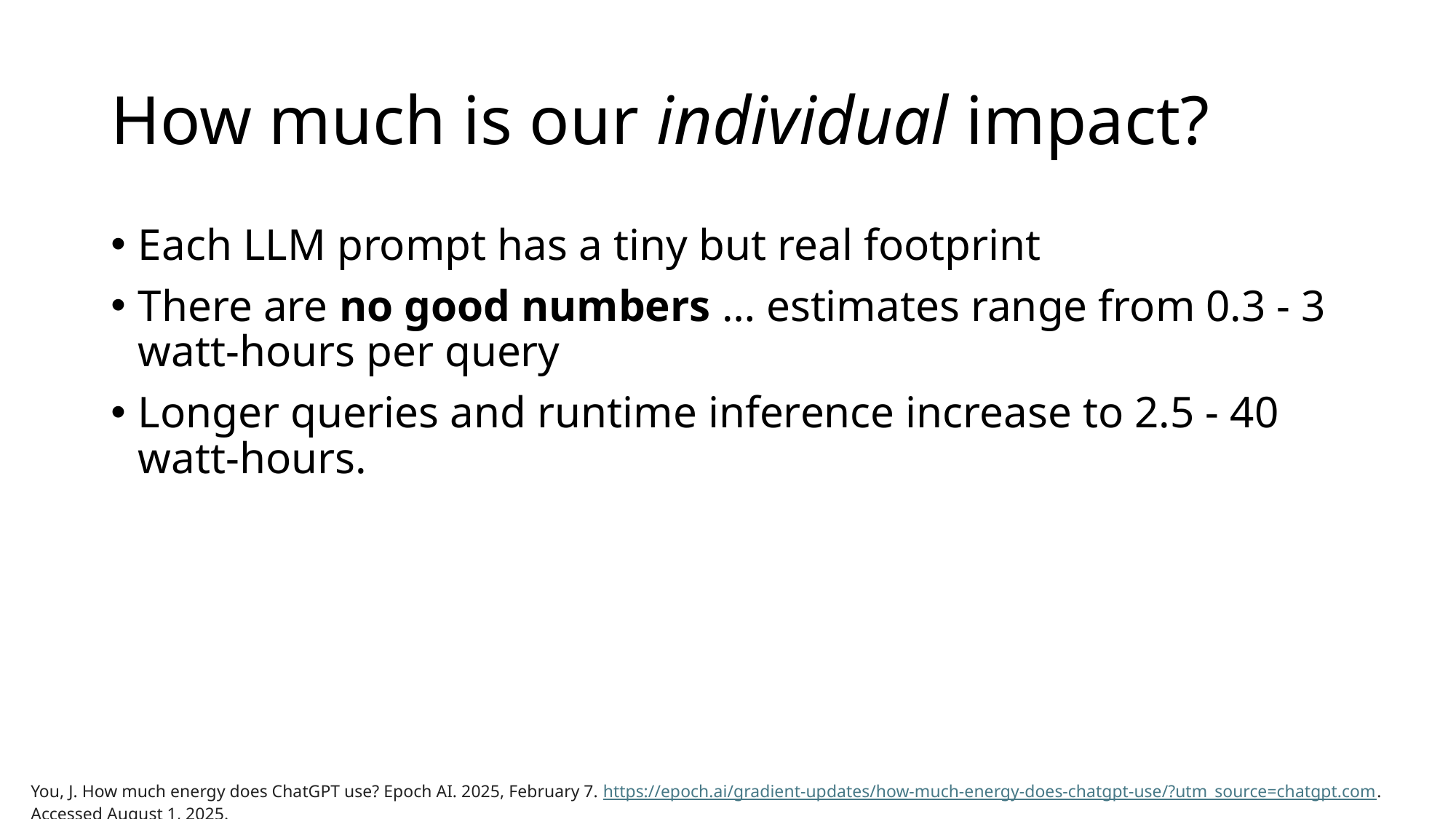

# How much is our individual impact?
Each LLM prompt has a tiny but real footprint
There are no good numbers … estimates range from 0.3 - 3 watt-hours per query
Longer queries and runtime inference increase to 2.5 - 40 watt-hours.
You, J. How much energy does ChatGPT use? Epoch AI. 2025, February 7. https://epoch.ai/gradient-updates/how-much-energy-does-chatgpt-use/?utm_source=chatgpt.com. Accessed August 1, 2025.

## Slide 10
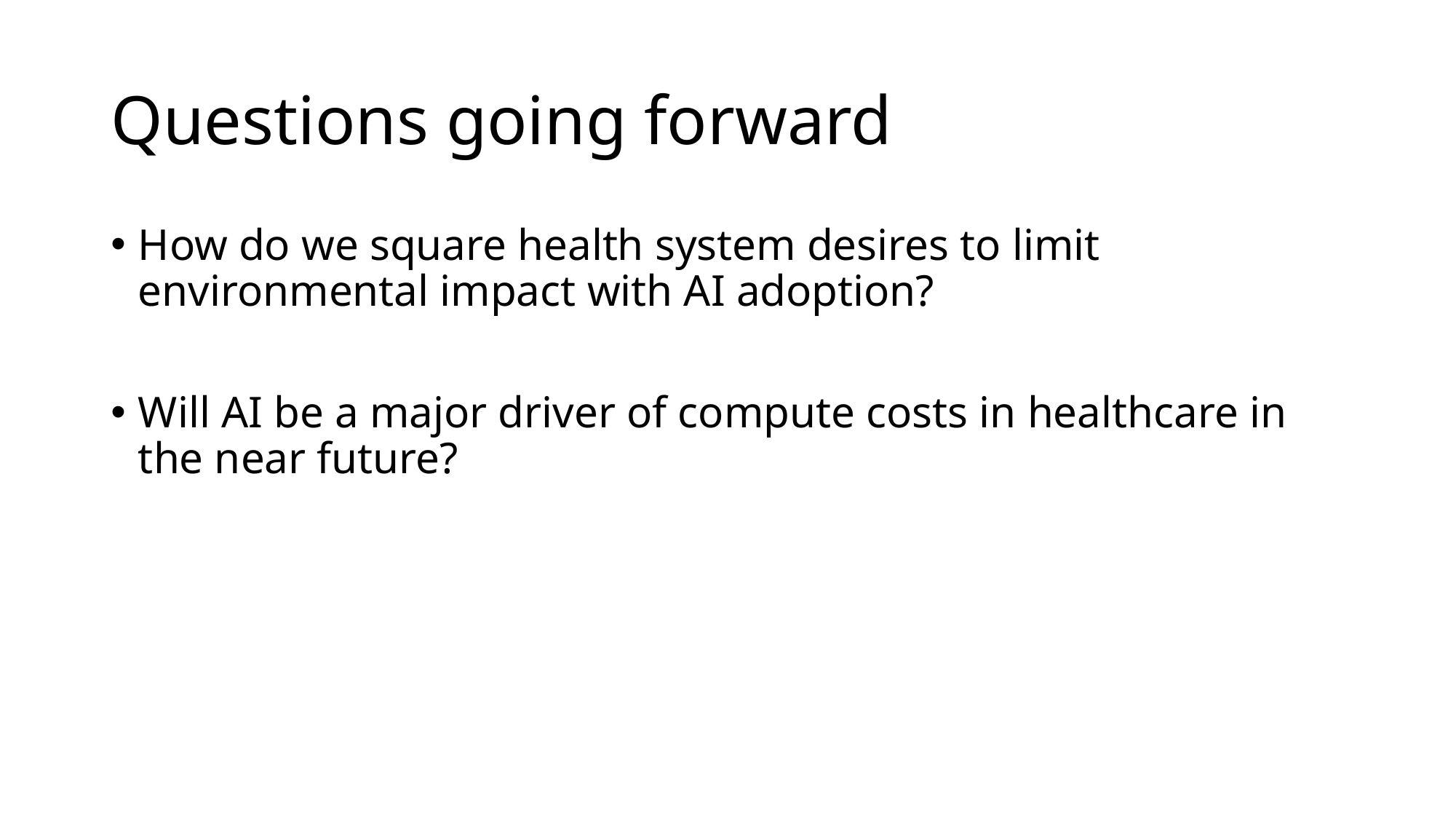

Questions going forward
How do we square health system desires to limit environmental impact with AI adoption?
Will AI be a major driver of compute costs in healthcare in the near future?

## Slide 11
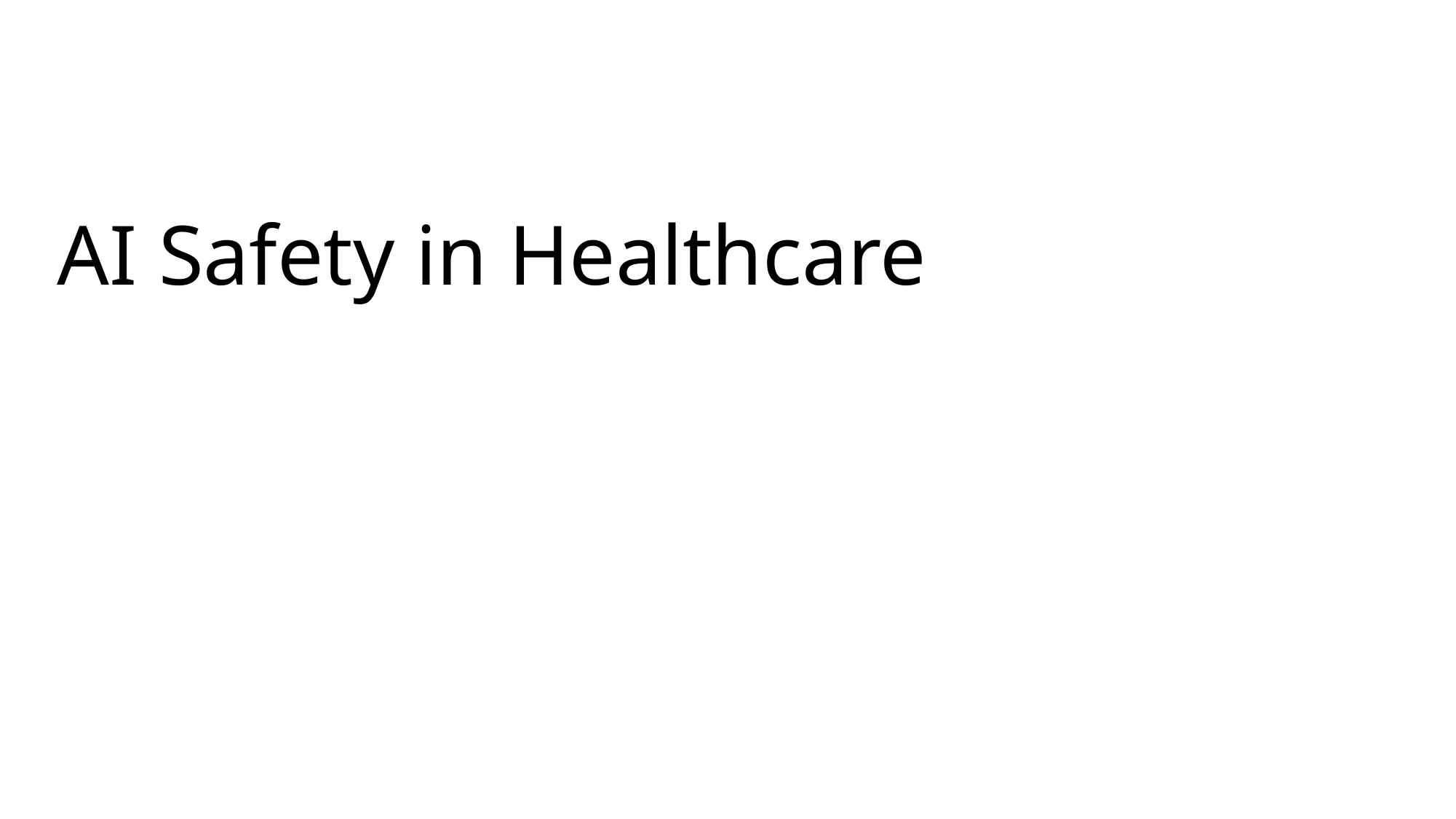

AI Safety in Healthcare

## Slide 12
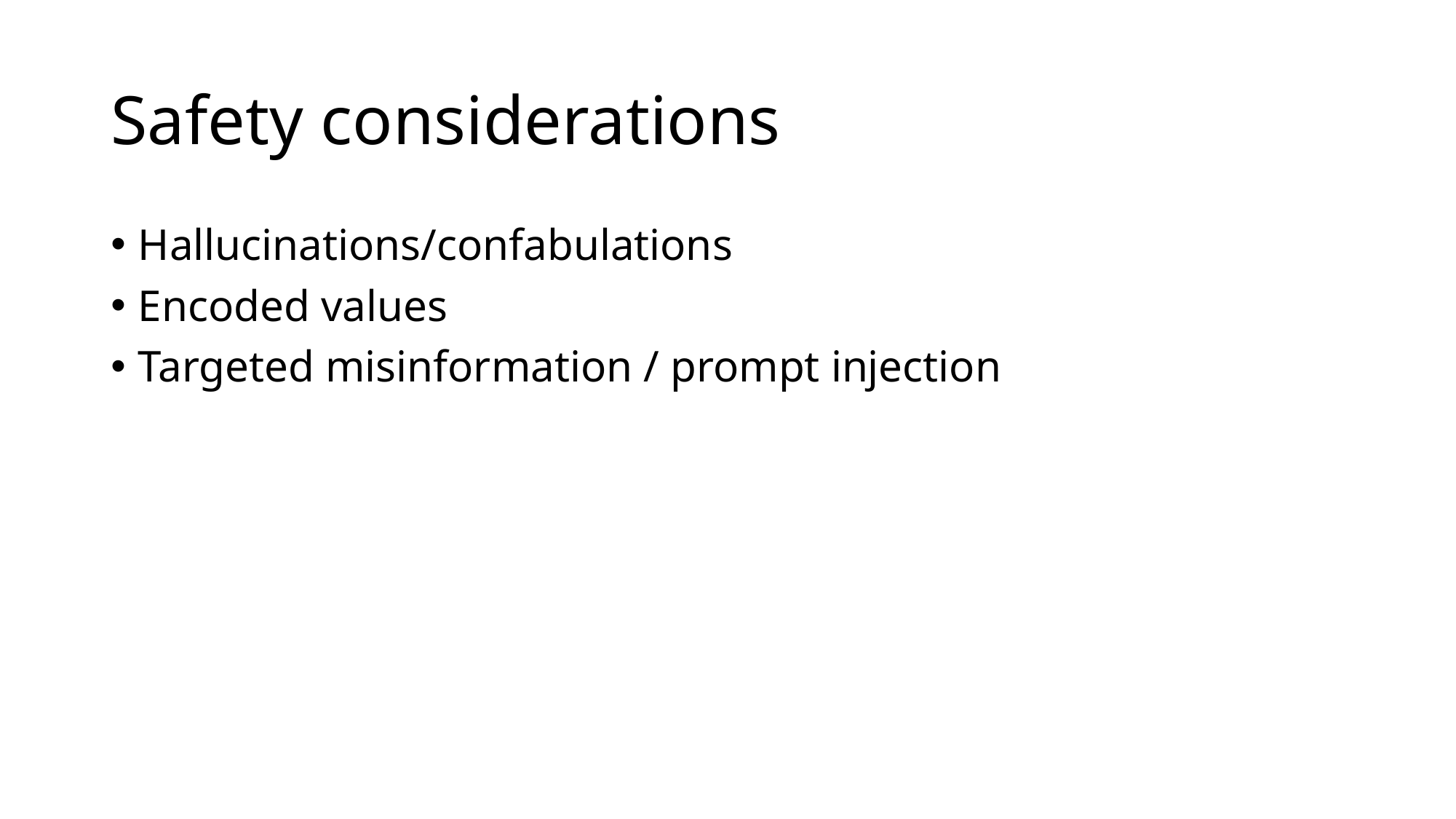

# Safety considerations
Hallucinations/confabulations
Encoded values
Targeted misinformation / prompt injection

## Slide 13
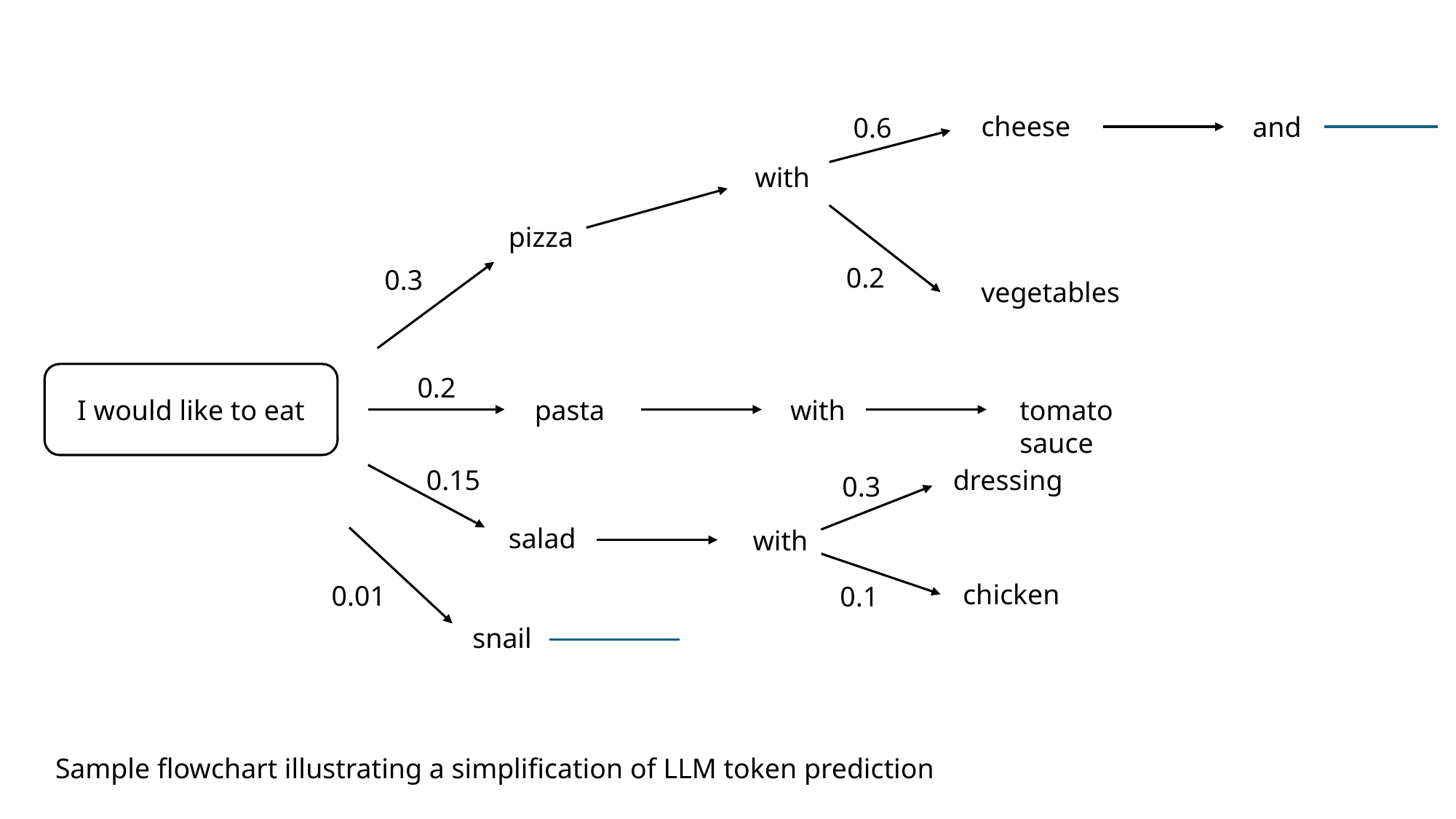

cheese
and
0.6
with
pizza
0.2
0.3
vegetables
I would like to eat
0.2
pasta
with
tomato sauce
dressing
0.15
0.3
salad
with
chicken
0.01
0.1
snail
Sample flowchart illustrating a simplification of LLM token prediction

## Slide 14
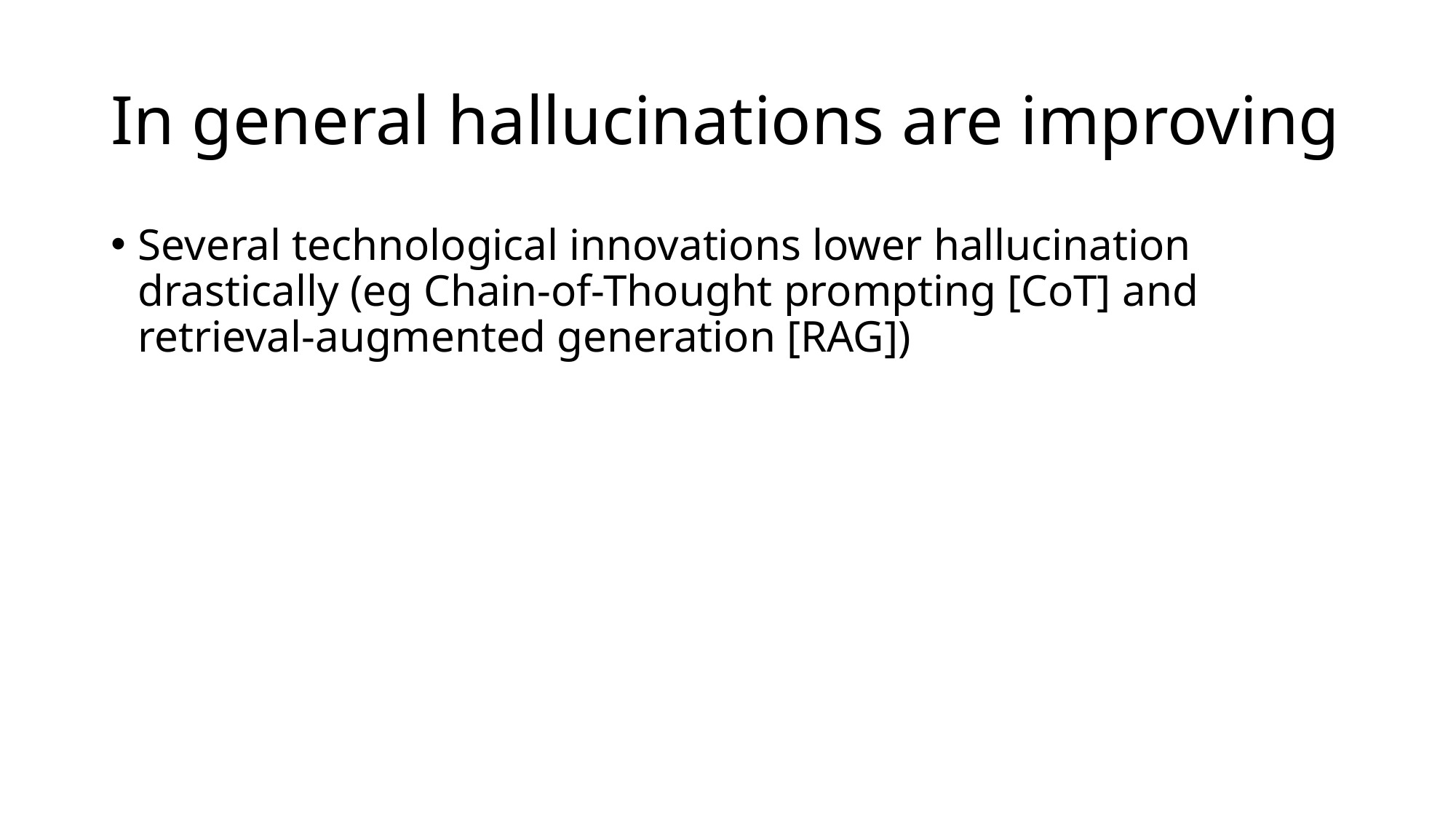

# In general hallucinations are improving
Several technological innovations lower hallucination drastically (eg Chain-of-Thought prompting [CoT] and retrieval-augmented generation [RAG])

## Slide 15
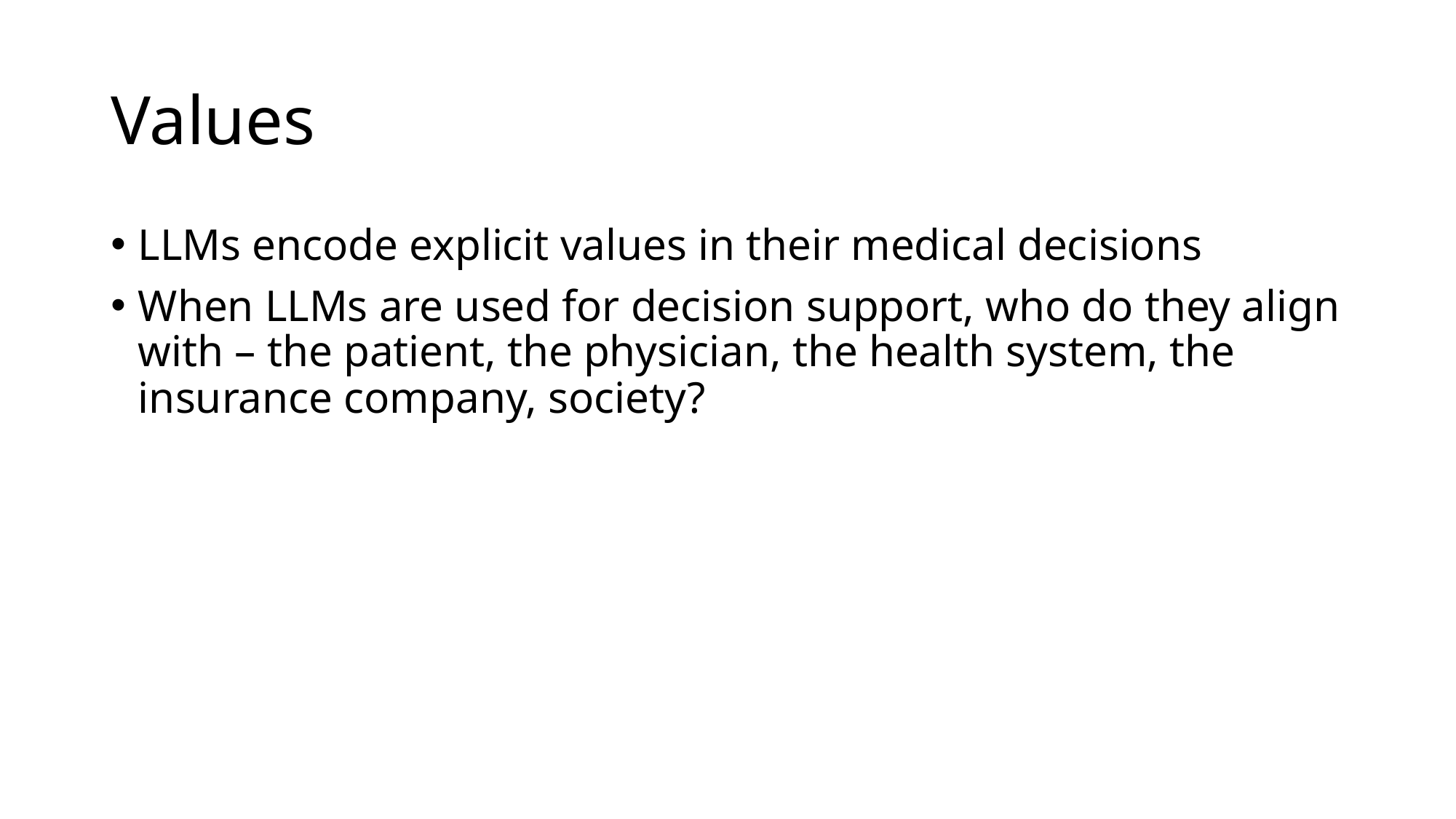

# Values
LLMs encode explicit values in their medical decisions
When LLMs are used for decision support, who do they align with – the patient, the physician, the health system, the insurance company, society?

## Slide 16
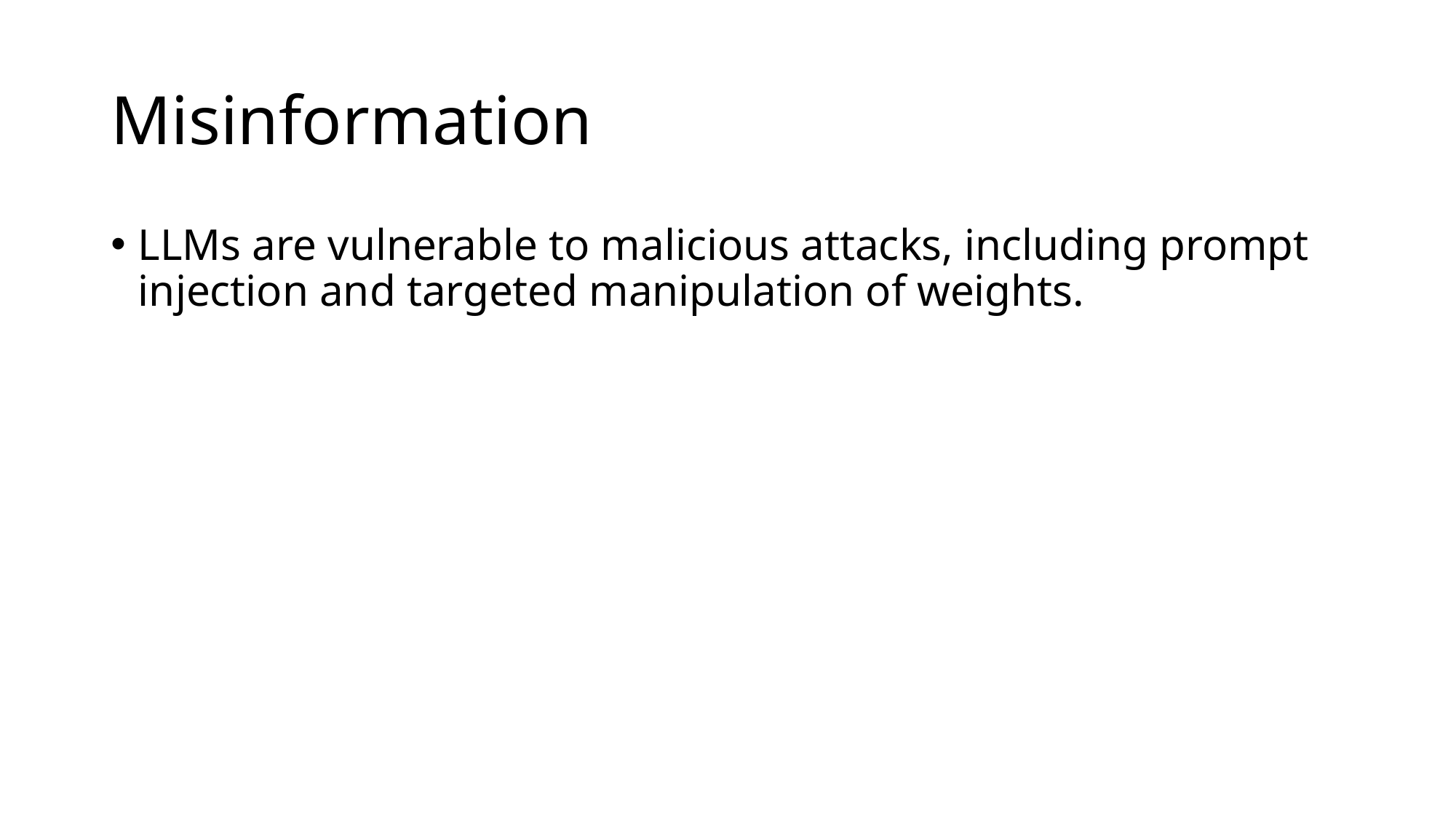

# Misinformation
LLMs are vulnerable to malicious attacks, including prompt injection and targeted manipulation of weights.

## Slide 17
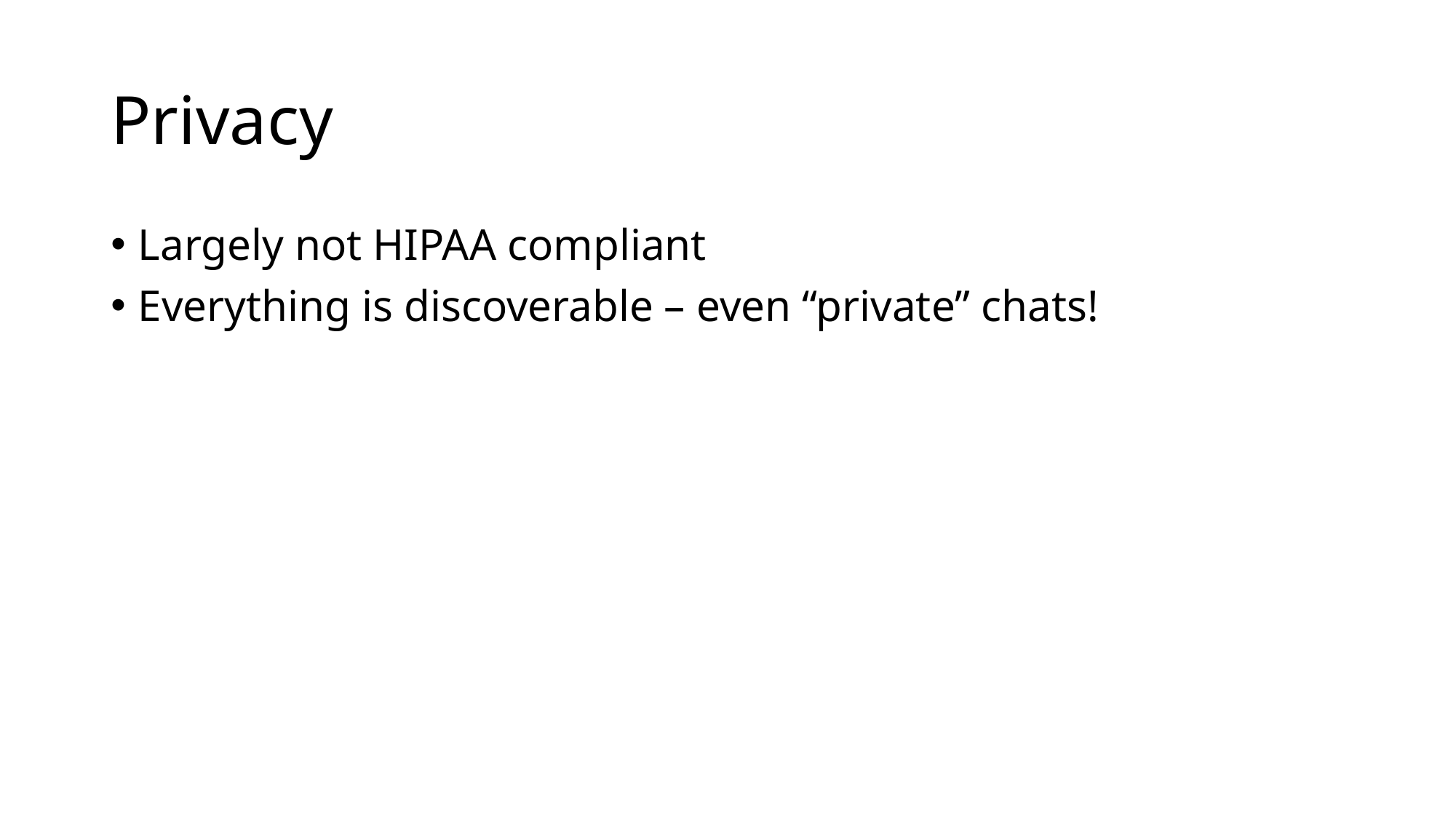

# Privacy
Largely not HIPAA compliant
Everything is discoverable – even “private” chats!

## Slide 18
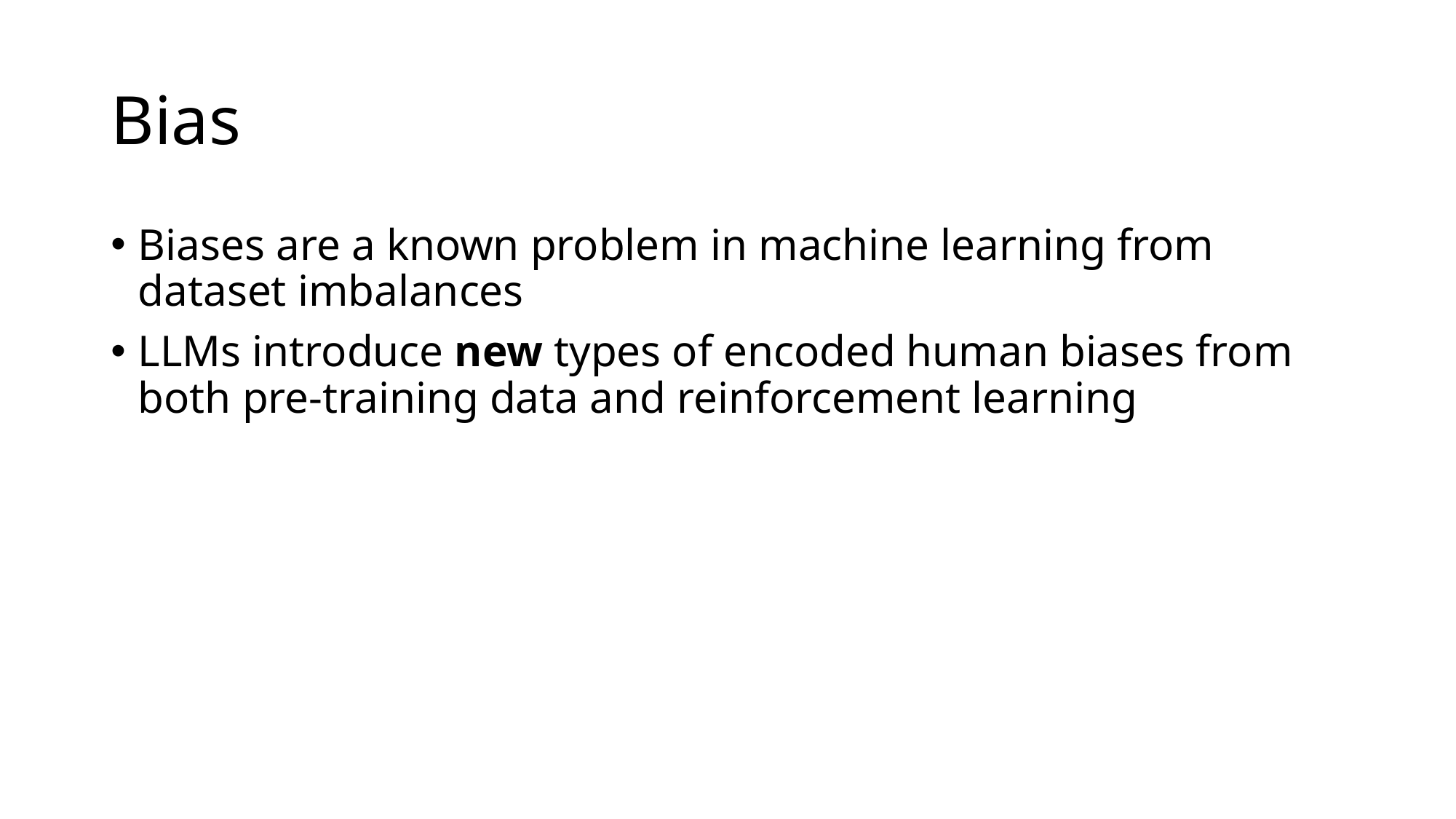

# Bias
Biases are a known problem in machine learning from dataset imbalances
LLMs introduce new types of encoded human biases from both pre-training data and reinforcement learning

## Slide 19
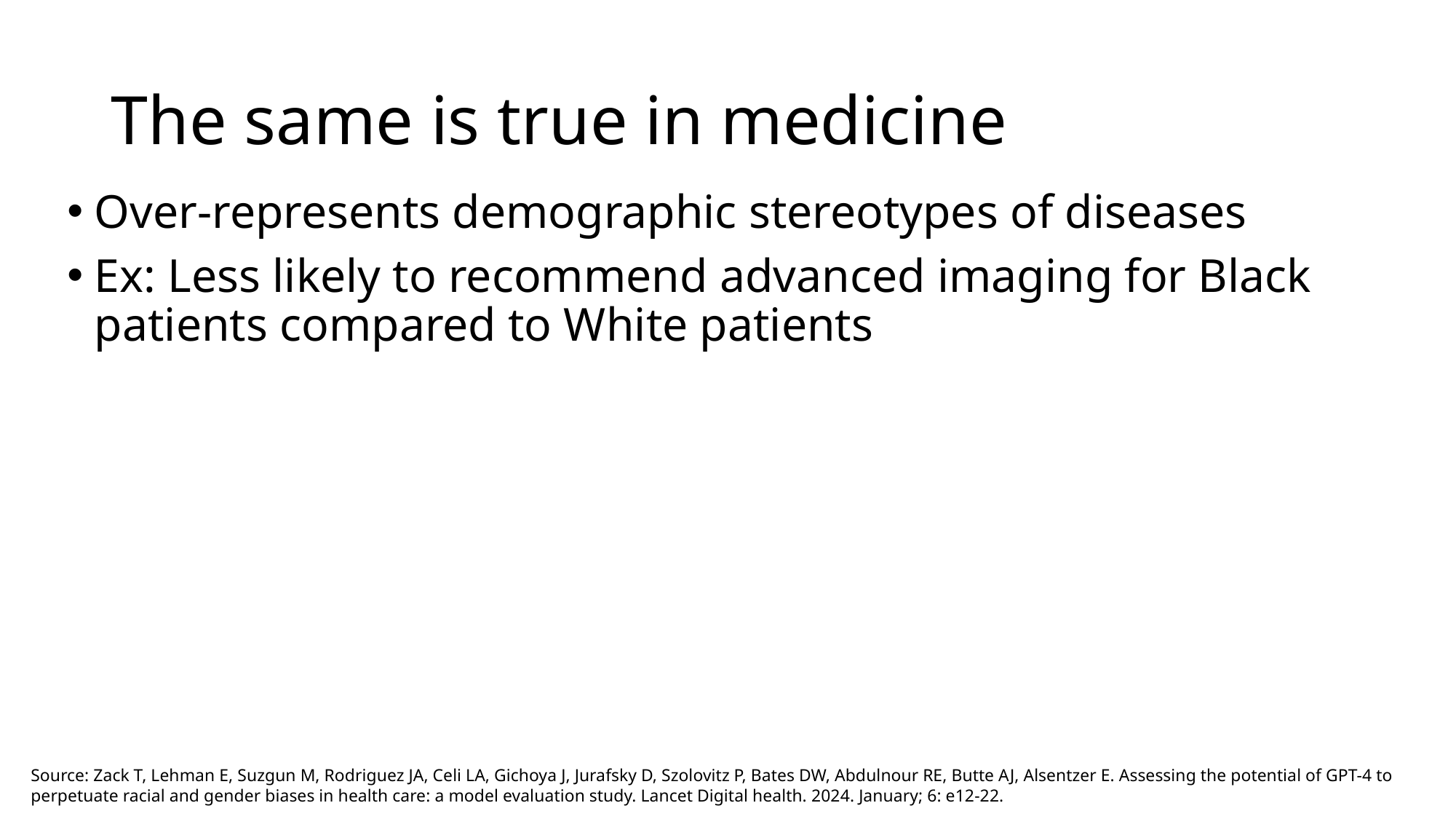

# The same is true in medicine
Over-represents demographic stereotypes of diseases
Ex: Less likely to recommend advanced imaging for Black patients compared to White patients
Source: Zack T, Lehman E, Suzgun M, Rodriguez JA, Celi LA, Gichoya J, Jurafsky D, Szolovitz P, Bates DW, Abdulnour RE, Butte AJ, Alsentzer E. Assessing the potential of GPT-4 to perpetuate racial and gender biases in health care: a model evaluation study. Lancet Digital health. 2024. January; 6: e12-22.

## Slide 20
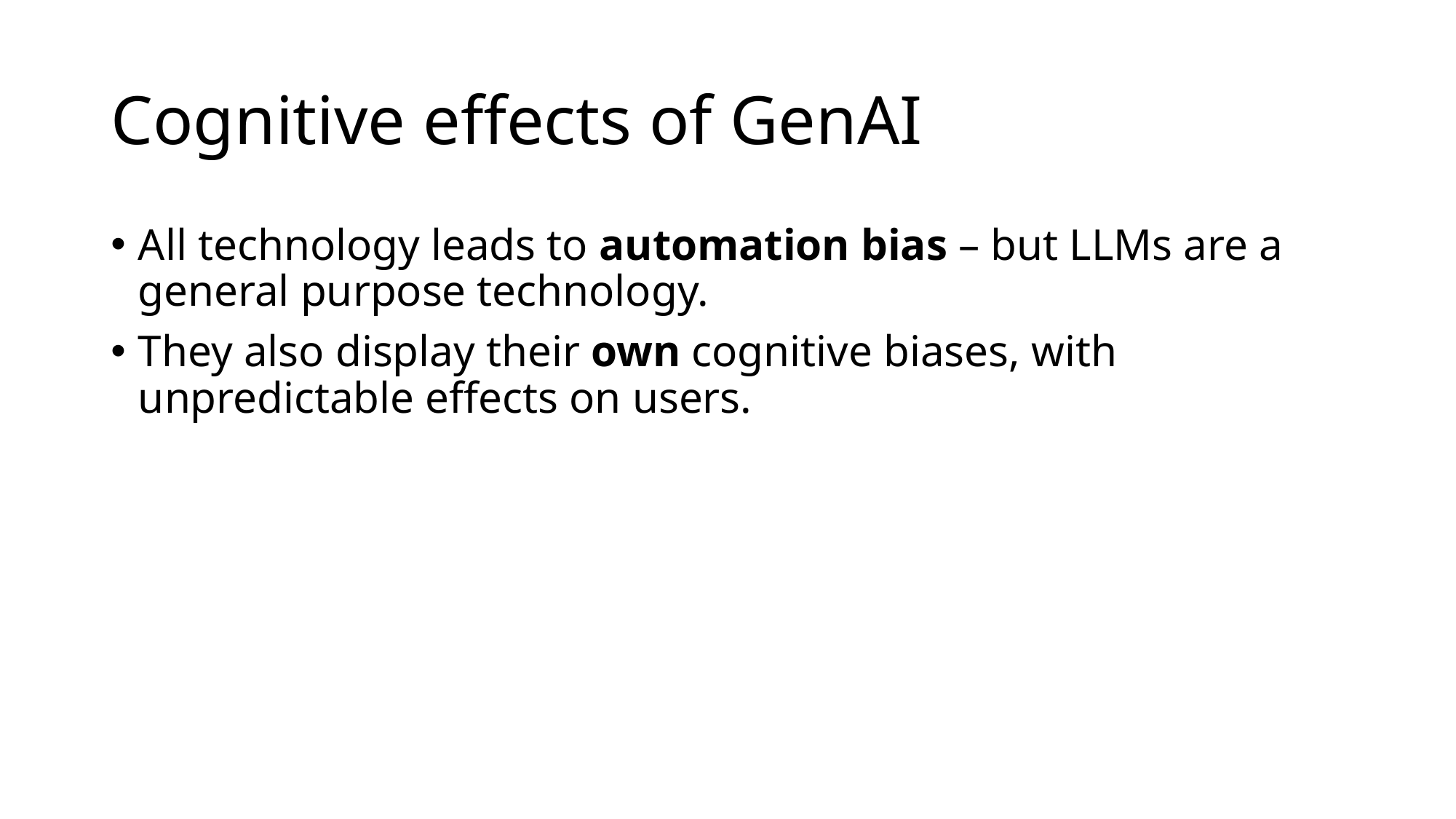

# Cognitive effects of GenAI
All technology leads to automation bias – but LLMs are a general purpose technology.
They also display their own cognitive biases, with unpredictable effects on users.

## Slide 21
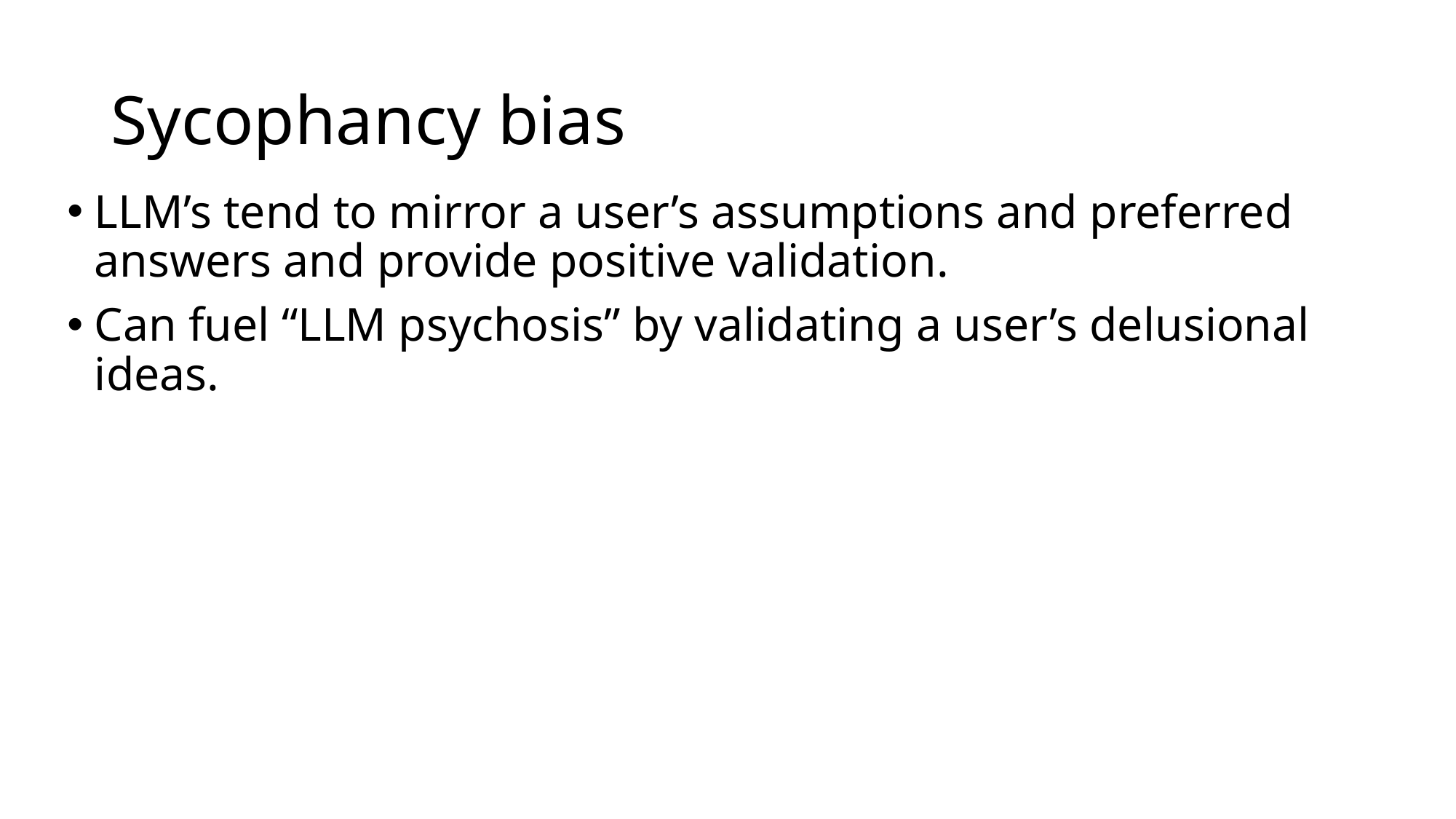

# Sycophancy bias
LLM’s tend to mirror a user’s assumptions and preferred answers and provide positive validation.
Can fuel “LLM psychosis” by validating a user’s delusional ideas.

## Slide 22
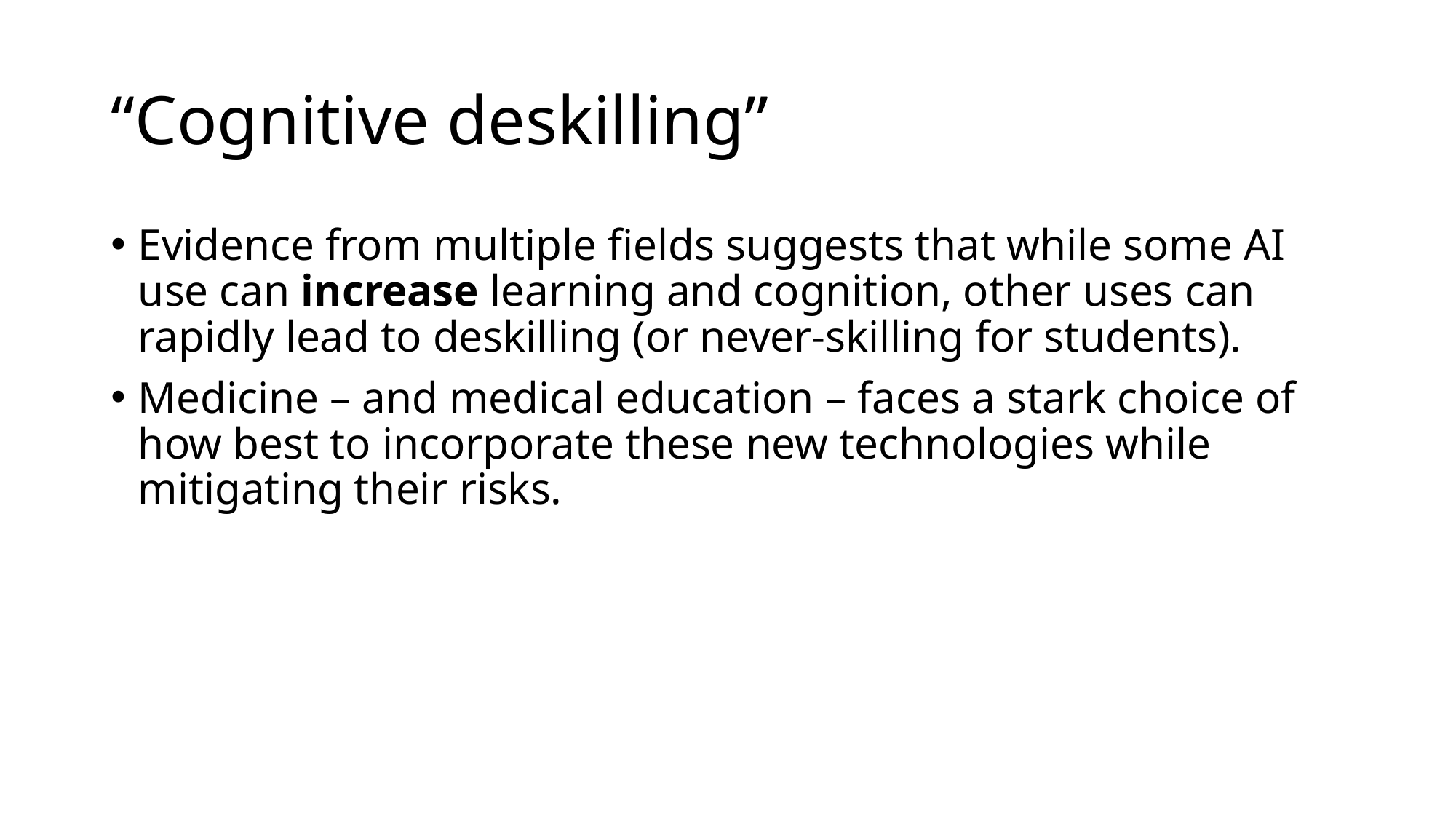

# “Cognitive deskilling”
Evidence from multiple fields suggests that while some AI use can increase learning and cognition, other uses can rapidly lead to deskilling (or never-skilling for students).
Medicine – and medical education – faces a stark choice of how best to incorporate these new technologies while mitigating their risks.

## Slide 23
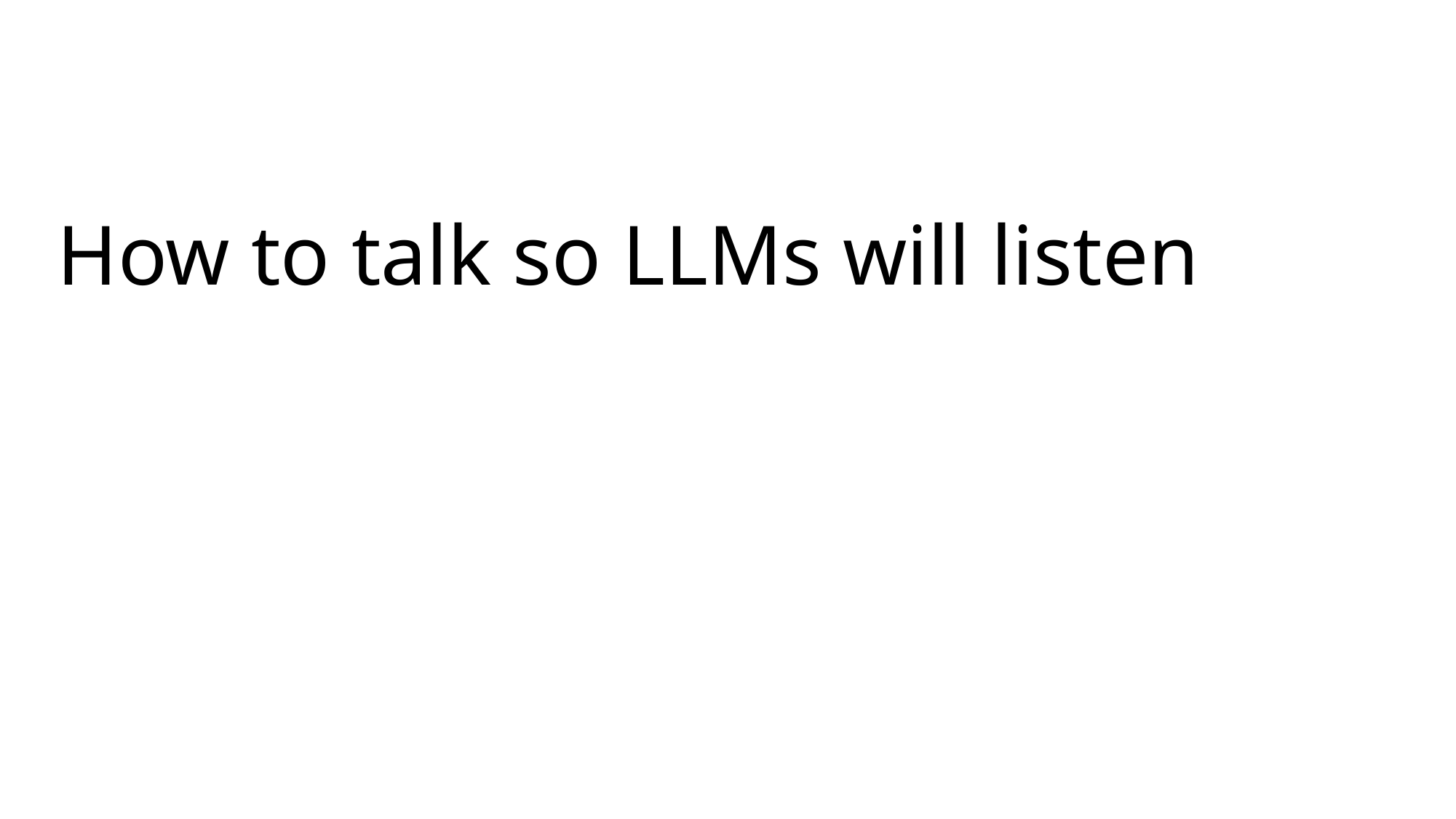

How to talk so LLMs will listen

## Slide 24
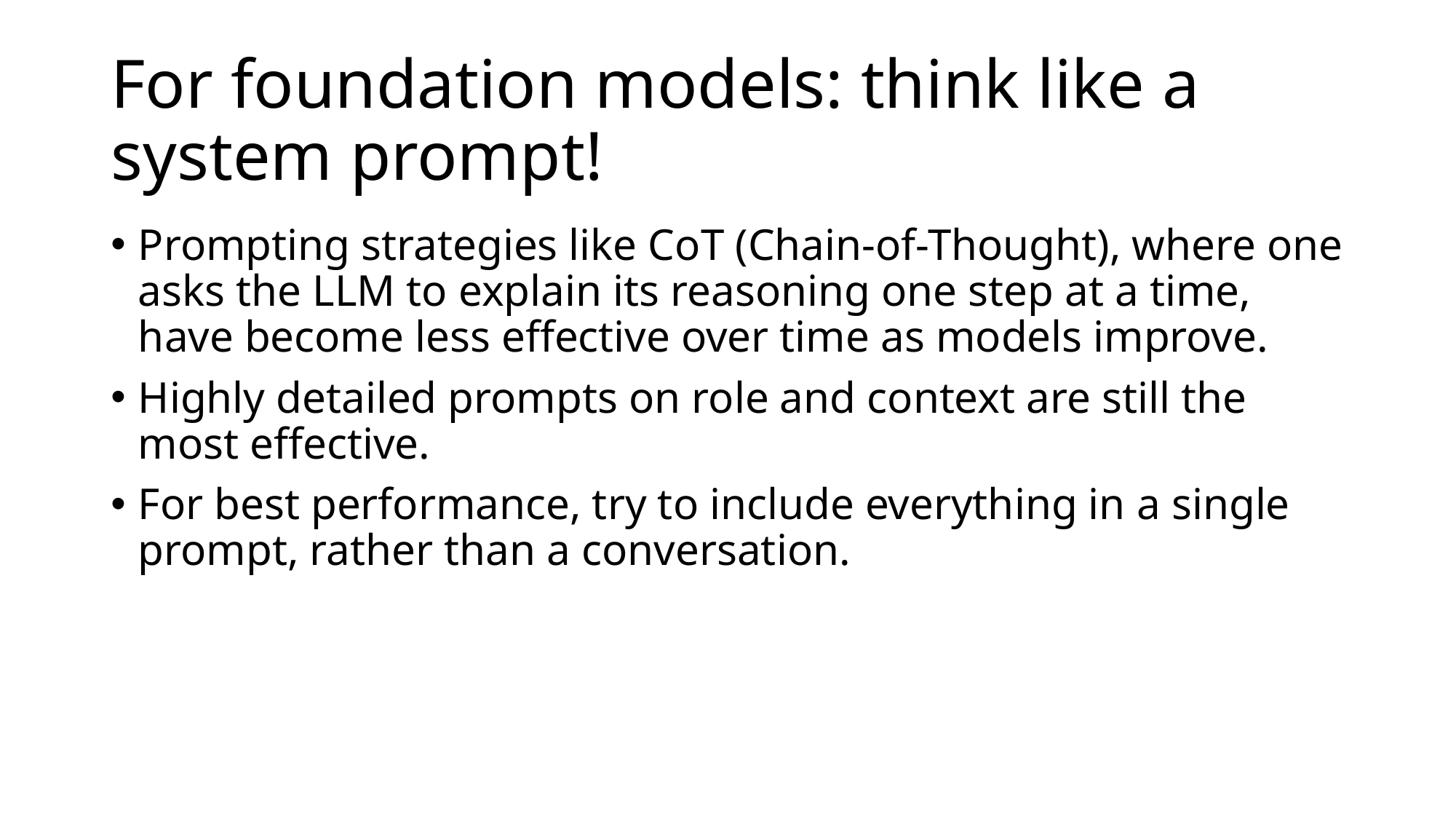

# For foundation models: think like a system prompt!
Prompting strategies like CoT (Chain-of-Thought), where one asks the LLM to explain its reasoning one step at a time, have become less effective over time as models improve.
Highly detailed prompts on role and context are still the most effective.
For best performance, try to include everything in a single prompt, rather than a conversation.

## Slide 25
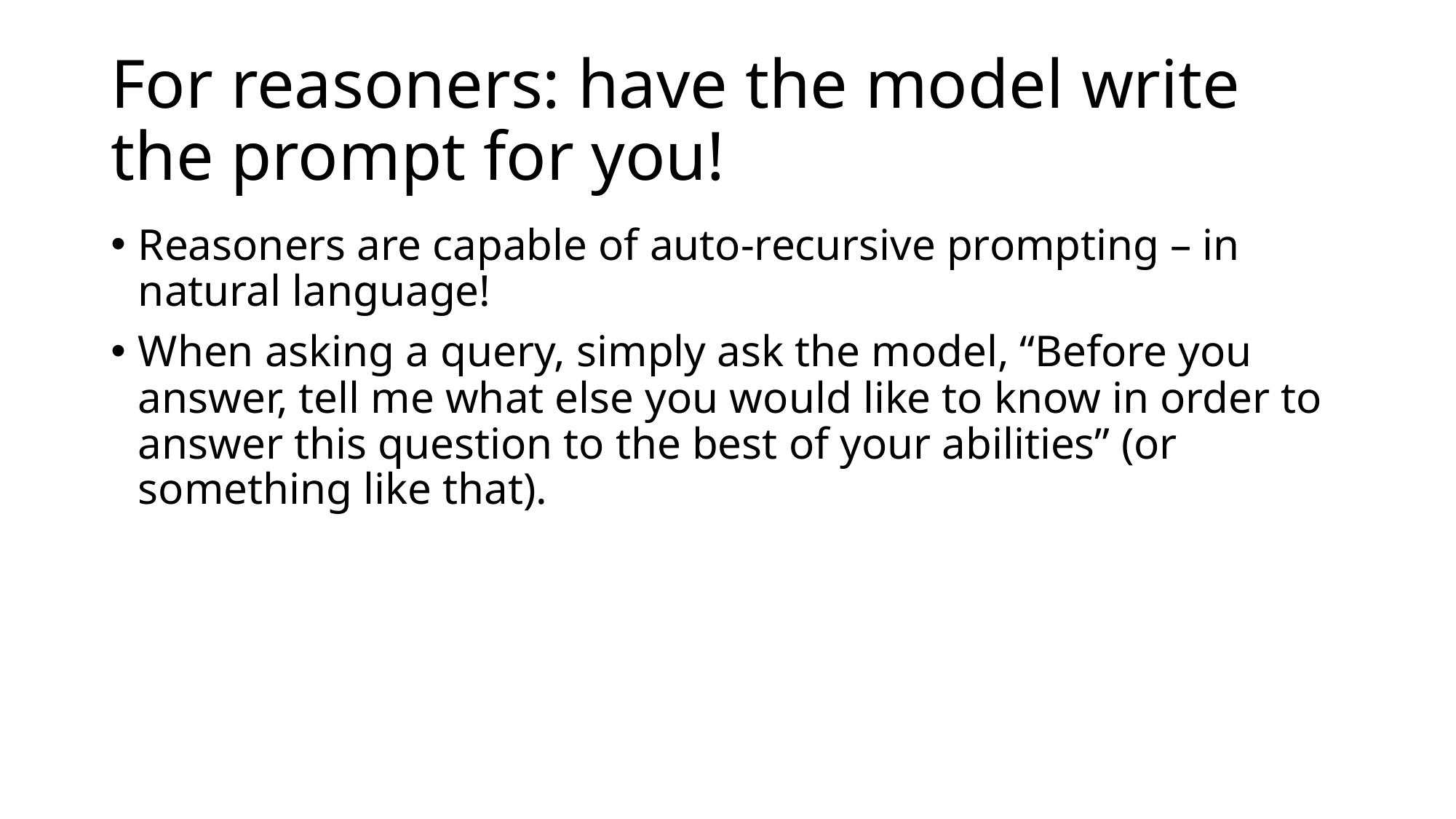

# For reasoners: have the model write the prompt for you!
Reasoners are capable of auto-recursive prompting – in natural language!
When asking a query, simply ask the model, “Before you answer, tell me what else you would like to know in order to answer this question to the best of your abilities” (or something like that).

## Slide 26
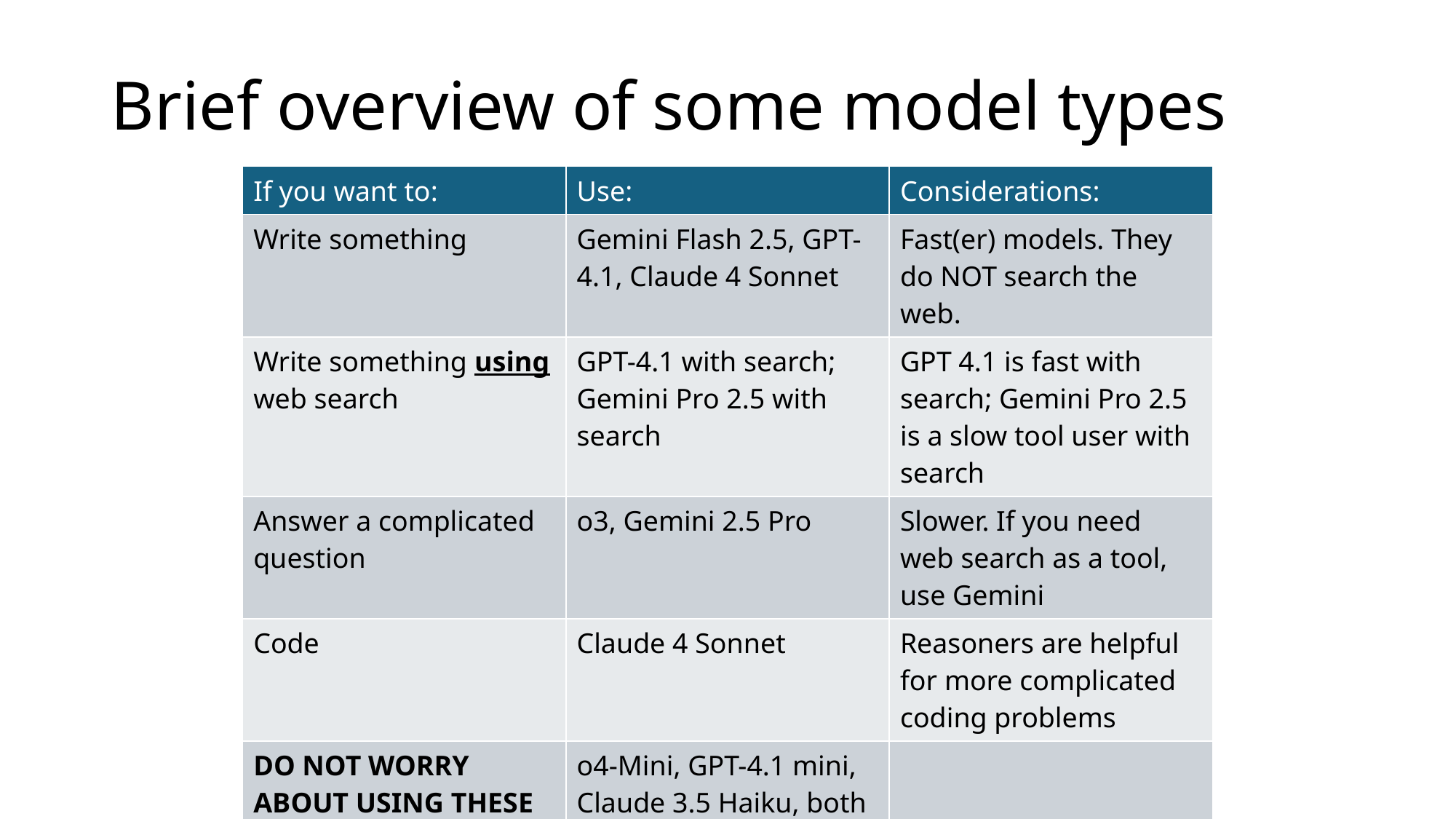

# Brief overview of some model types
| If you want to: | Use: | Considerations: |
| --- | --- | --- |
| Write something | Gemini Flash 2.5, GPT-4.1, Claude 4 Sonnet | Fast(er) models. They do NOT search the web. |
| Write something using web search | GPT-4.1 with search; Gemini Pro 2.5 with search | GPT 4.1 is fast with search; Gemini Pro 2.5 is a slow tool user with search |
| Answer a complicated question | o3, Gemini 2.5 Pro | Slower. If you need web search as a tool, use Gemini |
| Code | Claude 4 Sonnet | Reasoners are helpful for more complicated coding problems |
| DO NOT WORRY ABOUT USING THESE MODELS FOR MOST MEDICAL STUDENT USES | o4-Mini, GPT-4.1 mini, Claude 3.5 Haiku, both Llama models, Code Interpreter | |
